# Amazon Rainforest Hidden Volatiles—Part I: Unveiling New Compounds from *Acmella oleracea* (L.) R.K. Jansen Essential Oil

**DOI:** 10.3390/plants13121690

**Published:** 2024-06-19

**Authors:** Niko S. Radulović, Marko Z. Mladenović, Clarissa Silva Lima, Elza Caroline Alves Müller, Elizabeth Vianna Moraes da Costa, Rozilene Valadares Martins, Fabio Boylan

**Affiliations:** 1 Department of Chemistry, Faculty of Sciences and Mathematics, University of Niš, Višegradska 33, 18000 Niš, Serbia; markohem87@gmail.com; 2 Department of Biological Sciences and Health, Federal University of Amapá, Highway Juscelino Kubitschek, Km 02, Macapá 68903-197, Brazil; lima.clarissa@gmail.com (C.S.L.); elzacaroline@gmail.com (E.C.A.M.); elizabethviana@unifap.br (E.V.M.d.C.); 3 Postgraduate Program in Health Sciences, Federal University of Amapá, Highway Juscelino Kubitschek, Km 02, Macapá 68903-197, Brazil; lenefsm@gmail.com; 4 School of Pharmacy and Pharmaceutical Sciences, Panoz Institute, and Trinity Biomedical Sciences Institute, Trinity College Dublin, Dublin 2, D02 PN40 Dublin, Ireland

**Keywords:** *Acmella oleracea*, essential oil, esters, NMR, GC-MS, structure elucidation

## Abstract

Motivated by the culinary and ethnopharmacological use of *Acmella oleracea* (L.) R.K. Jansen, this study aimed to unveil new chemical compounds from its essential oil (EO). *Acmella oleracea*, known for its anesthetic and spicy properties, has been used in traditional medicine and cuisine, particularly in Northern Brazil. Through a detailed GC-MS analysis, 180 constituents were identified, including 12 tentatively identified long-chain α-keto esters of various acids. Additionally, 18 new esters were synthesized for structural verification. This research expands the known chemical diversity of *A. oleracea* EO, providing a basis for potential pharmacological applications. The identification of new natural products, including homologs and analogs of acmellonate, underscores the EO’s rich chemical profile and its potential for novel bioproduct development.

## 1. Introduction

The Amazon rainforest, known for its immense biodiversity, harbors countless plant species with significant pharmacological potential [1]. One of these species is *Acmella oleracea* (L.) R.K. Jansen, an annual herb found in the Amazon and other tropical regions, used for its anesthetic and spicy properties in Northern Brazilian cuisine and traditional medicine [2]. It has been widely cultivated for medicinal purposes besides horticultural and culinary. This plant’s pharmacological properties include but are not limited to, antinociceptive, anti-inflammatory, antioxidant, immunomodulatory, antimicrobial, antiviral, and diuretic activity [1,2,3]. The main biological activities of this plant are linked to (2*E*,6*Z*,8*E*)-*N*-isobutyl-2,6,8-decatrienamide (syn. spilanthol).

In the last decades, the essential oil (EO) of *A. oleracea* has been gaining more attention regarding its biological properties. Insecticidal activity against the southern house mosquito (*Culex quinquefasciatus*), the African cotton leafworm (*Spodoptera littoralis*), and the common housefly (*Musca domestica*) are of particular interest [3]. Despite several studies on its EO, the chemical composition of *A. oleracea* remains incompletely understood [3,4,5,6,7,8]. This study aims to reanalyze the EO composition of *A. oleracea* to discover new compounds and validate traditional uses. The identification of selected EO constituents required chromatographic fractionation of the full EO, followed by derivatization of the fractions by dimethyl disulfide, synthesis of certain major and minor components, and spectral (IR, MS, 1D-, and 2D-NMR) characterization of the EO fractions and synthesized constituents. By identifying novel chemical constituents, we aim to contribute to the development of new bioproducts with potential pharmacological applications.

## 2. Results and Discussion

### 2.1. Composition of A. oleracea Essential Oil and Essential Oil Fractions

**Figure 1:**
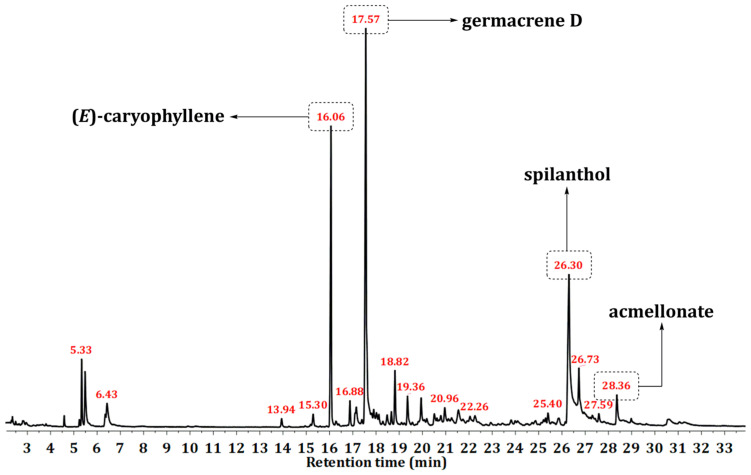
Typical TIC (total ion current) chromatogram of *A. oleracea* essential oil.

The EO of *A. oleracea* was subjected to GC-MS analysis (Figure 1), revealing 180 constituents, including new long-chain α-keto esters (Table 1). The identified constituents represented 97.0% of the total detected areas of the GC chromatogram of EO, with fatty-acid-related compounds (alkylamides and keto esters) and sesquiterpenoids as the most abundant compound classes. Spilanthol (28.9%), germacrene D (17.2%), (*E*)-caryophyllene (5.2%), pentadec-1-ene (4.8%), and acmellonate (4.7%) represented the major EO constituents (Table 1). A comparison of the identified constituents with the previously published data revealed that the herein presented composition (Table 1) was qualitatively and quantitatively different from those previously published [3,4,5,6,7,8].

One homologous series of constituents immediately caught our attention—long-chain keto esters of (iso)butanoic and isomeric (un)saturated pentanoic acids. Similar MS fragmentation patterns of four pairs of constituents (twelve in total) with linear changes (ca. 100 RI units) of the RI values suggested that these constituents represent homologous esters of long-chain saturated keto alcohols with different numbers of carbon atoms (pairs of constituents with RI values (1769, 1863, 1908, 1948), (1869, 1964, 2009, 2049), and (1969, 2065, 2110, 2150)). The reasoning that these constituents could be esters of long-chain saturated keto alcohols was based on the similarities between characteristic MS fragmentation patterns in the previously identified acmellonate (syn. (7*Z*,9*E*)-2-oxoundeca-7,9-dien-1-yl senecioate; RI = 1995) and the detected constituent at RI = 1948 (Figure S1). In some cases, the presence of intense ions at *m*/*z* 155, 169, or 183, which are indicative of C_10_H_19_O^+^, C_11_H_21_O^+^, and C_12_H_23_O^+^ moieties, respectively, and base ions at *m*/*z* 71, 85, or 83, which is typical mass fragmentation for (iso)butanoates (C_4_H_7_O^+^), isomeric pentanoates (C_5_H_9_O^+^), and isomeric 2-pentenoates (C_5_H_7_O^+^), respectively, provided us crucial information in their tentative identification.

**Figure 2:**
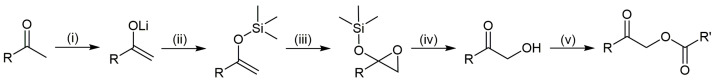
Synthesis of 2-oxoundecyl, 2-oxododecyl, and 2-oxotridecyl 2-methylpropanoates, 2-methylbutanoates, 3-methylbutanoates, angelates, tiglates, and senecioates (R = n-nonyl, n-decyl, and n-undecyl): (i) LDA, dry THF, −78 °C, 10 min; (ii) (CH_3_)_3_SiCl, dry THF, rt, 24 h; (iii) m-CPBA, CH_2_Cl_2_, 0 °C, 24 h; (iv) H_2_SO_4_, H_2_O, rt, 2 h; (v) R’COOH, DCC, DMAP, and CH_2_Cl_2_.

Unfortunately, these constituents could not be isolated from the EO sample due to their low relative abundance and the complexity of the EO. The ‘dry-flash’ chromatographic separation performed on SiO_2_ of an EO portion, aimed to obtain pure EO constituents, resulted in fractions rich with such constituents (Table 1 and Figure S2). Thus, we focused our attention on the first eluting ester-containing fraction (F5 from Table 1), as it contained numerous minor esters that were hardly detectable in the initial GC-MS analyses of the unfractionated EO. The specific keto alcohols (1-hydroxyundecan-2-one, 1-hydroxydodecan-2-one, and 1-hydroxytridecan-2-one) needed to prepare the synthetic samples of esters for a direct comparison were commercially unavailable. For that reason, we followed an approach that included two parts: the synthesis of keto alcohols and the preparation of a small synthetic library of 18 esters (2-methylpropanoates, 2-methylbutanoates, 3-methylbutanoates, angelates, tiglates, and senecioates) via the Steglich procedure (Figure 2). All synthesized compounds represented new compounds at the time of the investigation. The creation of a small synthetic library enabled the identification of 12 new natural products. Notably, the identified esters, such as 2-oxoundecyl and 2-oxotridecyl derivatives, have not been previously reported. These findings enhance our understanding of the EO’s chemical profile and suggest potential pharmacological applications, validating its traditional uses.

### 2.2. NMR Spectral Characterization of the New Esters

The obtained esters and starting keto alcohols were subjected, beside MS and IR measurements, to a battery of 1D- (^1^H and ^13^C, including ^1^H spectra with homonuclear and ^13^C spectra without heteronuclear decoupling, as well as DEPT90 and DEPT135) and 2D- (gradient NOESY, HSQC, and HMBC) NMR experiments. The spectral data and assignments are summarized in the Experimental Section and Supplementary Materials (Figures S3–S43), whereas a numbering scheme of C atoms is presented in Figure 3.

**Figure 3:**
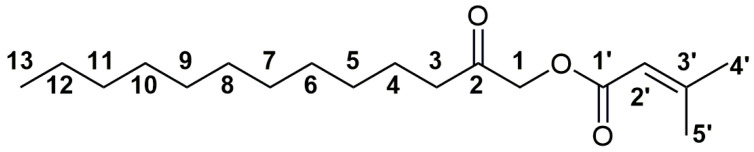
The structure of 2-oxotridecyl 3-methyl-2-butenoate (syn. 2-oxotridecyl senecioate) (**3f**) with the carbon atom numbering scheme.

The assignment of signals is discussed in detail for one of the selected new natural products with the highest relative amount in the EO—2-oxotridecyl senecioate (**3f**). Both ^1^H and ^13^C NMR spectra of the esters from the same subfamily of compounds (**1a**–**1f**) differed only in the signals of the atoms from the acid moieties, whereas the opposite was true when comparing the spectra of the esters of different alcohols (**1**–**3**) and the same acid (e.g., compounds **1f**, **2f**, and **3f**). The ^1^H and ^13^C NMR spectra of compound **3f** contained the expected number of signals (Supplementary Materials Figures S34 and S35). A singlet at 4.66 ppm was assigned to the methylene group at position 1 (Figure 3). The HSQC spectrum (Supplementary Materials Figure S40) enabled the assignation of the ^13^C NMR signal of the carbon atom from the same methylene group (C-1) at 67.36 ppm. The HMBC spectrum (Supplementary Materials Figure S41) showed a correlation between C-1 protons and two ^13^C NMR signals at 204.85 and 165.59 ppm that were assigned to C-1′ and C-2 carbon atoms from the keto and ester groups, respectively. Additional HMBC correlations of the C-2 carbon atom with a triplet at 2.43 ppm (*J* = 7.5 Hz), followed by analysis of the HSQC correlations, allowed the assignation of the signals for the C-3 and H-3 atoms at 38.87 ppm and 2.43 ppm, respectively. Using the same approach, based on HSQC, HMBC, DEPT90, and DEPT135 spectra, signals at 1.61 (quintet, *J* = 7.0 Hz), 1.33–1.21 (16H, overlapping peaks), and 0.87 (pseudo triplet, *J* = 7.0 Hz) were assigned to C-4, C-5–C-12, and C-13 protons, respectively. This approach only allowed additional assignations of the C-4 and C-13 carbon atoms (23.32 and 14.13, respectively) from the alkyl chain, whereas signals at 31.91, 29.60, 29.44, 29.35, 29.34, 29.17, and 22.40 ppm originated from C-5–C-12 atoms. Assignation of the acidic part in the 2-oxotridecyl senecioate and other synthesized esters was based on a previous analysis by Radulović et al. [9].

### 2.3. Identification of the Isomers/Homologs/Analogs of Acmellonate

**Figure 4:**
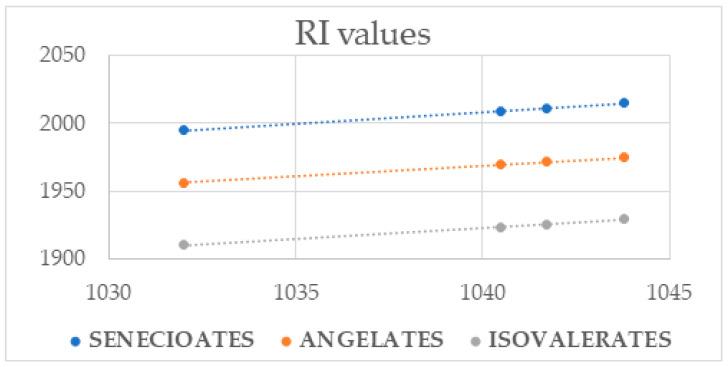
Correlation between RI data of stereoisomeric conjugated decadienes from the literature [11] and identified senecioates (y = 1.6778x + 263.39; R² = 0.9993), angelates (y = 1.5927x + 312.24; R² = 0.9991), and isovalerates (y = 1.5927x + 266.24; R² = 0.9991).

One of the identified major (4.3%) EO constituents was acmellonate (syn. (7*Z*,9*E*)-2-oxoundeca-7,9-dien-1-yl senecioate). The initial tentative GC-MS identification of this constituent was based solely on the matching of the corresponding retention indices (RI = 1995) and mass spectra with literature data [3,10]. The structure of the detected acmellonate was additionally corroborated by a comparison of the NMR spectral data (Figure S44) obtained for the EO fraction F5, which contained more than 30% of acmellonate, with the ones for acmellonate from the literature [10]. The MS fragmentation pattern of three detected constituents that eluted slightly later from the DB-5MS column compared to acmellonate (RI = 2009, 2011, and 2015) was almost identical to that of acmellonate. The observed differences in the retention index values were indicative of a relationship between geometric isomers, more specifically of the order of eluting of simpler dienic systems: (2*E*,4*Z*)-deca-2,4-diene, (2*E*,4*E*)-deca-2,4-diene, (2*Z*,4*E*)-deca-2,4-diene, and (2*Z*,4*Z*)-deca-2,4-diene, with RI values on a column of comparable polarity: 1032, 1040.5, 1041.7, and 1043.8, respectively [11]. With an excellent correlation of the RI values of the detected acmellonate isomers and of the isomeric deca-2,4-dienes from the literature [11], the detected isomers of the acmellonate at RI = 2009, 2011, and 2015 could be tentatively identified as (7*E*,9*E*)-2-oxoundeca-7,9-dien-1-yl, (7*E*,9*Z*)-2-oxoundeca-7,9-dien-1-yl, and (7*Z*,9*Z*)-2-oxoundeca-7,9-dien-1-yl senecioate, respectively (Figure 4).

**Figure 5:**
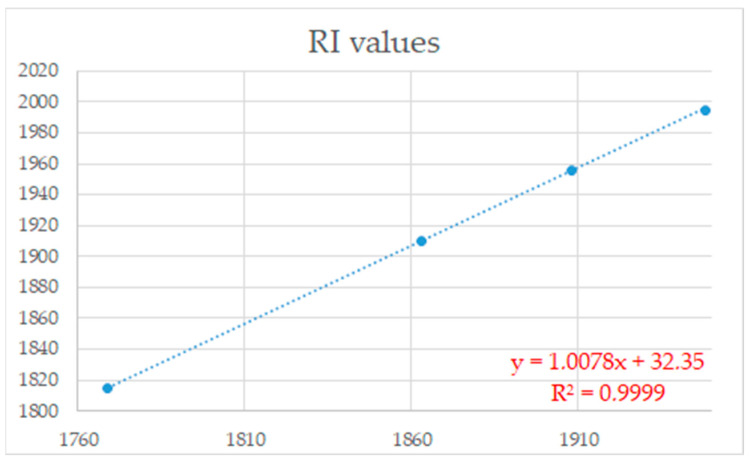
Correlation between RI data of the synthesized 2-oxoundecyl 2-methylpropanoate, 3-methylbutanoate, angelate, and senecioate with the identified 2-oxoundeca-7,9-dien-1-yl esters.

To more easily locate additional minor constituents with the MS fragmentation analogous to that of acmellonate, we generated a partial ion current (PIC) chromatogram of fraction F5 for the *m*/*z* 85, *m*/*z* 83, and *m*/*z* 71 ion currents (Figure S46). The generated PICs allowed us to detect nine different “acmellonate-like” compounds. MS fragmentation patterns suggested that these were also esters of the mentioned four different diastereoisomeric 2-oxoundeca-7,9-dien-1-ols, with a 2-pentenoic acid (tiglic or angelic acid), an isomeric pentanoic acid, and one with (iso)butanoic acid. The value of an analogously determined correlation coefficient between retention indices higher than 0.99 (Figure 4) further allowed us to propose that the detected constituents were also esters of the (7*Z*,9*E*)-, (7*E*,9*E*)-, (7*E*,9*Z*)-, and (7*Z*,9*Z*)-2-oxoundeca-7,9-dien-1-ol, respectively, whereas the correlation of the experimentally obtained RI values of the synthesized 2-oxoundecyl esters and the mentioned detected constituents (Figure 5) suggested that these were 3-methylbutanoates and angelates, rather than 2-methylbutanoates and tiglates.

**Figure 6:**
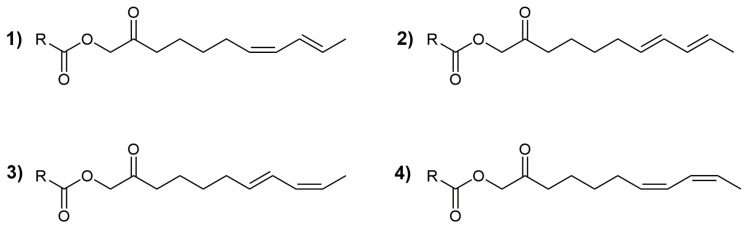
The structure of the identified esters (RCOO= isovalerate, angelate, and senecioate) of (7*Z*,9*E*)-, (7*E*,9*E*)-, (7*E*,9*Z*)-, or (7*Z*,9*Z*)-2-oxoundeca-7,9-dien-1-ol, **1**–**4**, respectively.

The observed correlation of RI data presented in Figure 5 also suggested that the remaining detected ester was (7*Z*,9*E*)-2-oxoundeca-7,9-dien-1-yl 2-methylpropanoate (RI = 1815). At the time of the investigation, except for acmellonate, all other identified esters of the diastereoisomeric 2-oxoundeca-7,9-dien-1-ols (12 compounds) represented new compounds and newly identified natural products (Figure 6).

A GC-MS analysis of the essential oil and essential-oil fraction ’rich’ with esters before and after derivatization with DMDS revealed the presence of an additional group of related constituents that eluted slightly faster compared to the n-chain saturated keto esters. Reaction with DMDS implied the existence of a non-conjugated double-bond somewhere in the keto alcohol moiety. For instance, the peak at 27.30 min with the molecular ion at *m*/*z* 266 and typical MS fragmentation of 2-pentenoates (Figure S47), eluting 8 RI units faster than 2-oxoundecyl senecioate, was tentatively identified as a 2-oxoundecen-1-yl senecioate. Additionally, the appearance of DMDS adducts in the chromatogram of the derivatized fraction F5 (Figure S48), having an appropriate molecular weight (at *m*/*z* 360, which comes from 266 + 94), confirmed our assumption. The MS of the DMDS adduct displayed a fragment ion at *m*/*z* 103 (CH_3_CH_2_CH_2_CHSCH_3_^+^) that implied the position of the double-bond at position 7 (Figure S48), i.e., 2-oxoundec-7-en-1-yl senecioate. Since other double-bond positions would give rise to a specific fragment ion at *m*/*z* CH_3_(CH_2_)_n_CHSCH_3_, we inspected PICs of the derivatized fractions for their presence (Figure S49) and found peaks of ten additional DMDS adducts of esters of 2-oxoundec-7-en-1-ol, 2-oxotridec-6-en-1-ol, and 2-oxotridec-7-en-1-ol. All of the mentioned esters represented new natural products as well as new compounds in general.

The presence of only one peak before and after DMDS derivatization pointed to a single specific isomer, either (*E*) or (*Z*) of the 2-oxoundec-7-en-1-yl, 2-oxotridec-6-en-1-yl, and 2-oxotridec-7-en-1-yl esters [12]. In analogy with the double-bond configuration of one of the major EO constituents, acmellonate, we expected *Z* configuration at position 7. The difference in the RI values for the internal alkenes with the different positions of the double-bond and the same configuration (e.g., (6*Z*)-tetradec-6-ene and (7*Z*)-tetradec-7-ene) was ca. 2 RI units, whereas the difference between alkenes with the same position of the double-bond but with the different configurations of the double-bond was higher than 5 units (e.g., difference in the RI data for (6*Z*)-tetradec-6-ene and (6*E*)-tetradec-6-ene is 7 units) [13]. The difference in the RI data for the detected 2-oxotridec-7-en-1-yl and 2-oxotridec-6-en-1-yl esters suggested different configurations of the double-bond in position 6 compared to the counterparts with the double-bond in position 7. However, the exact double-bond configuration requires additional research that includes synthesis and spectral characterization of stereoisomerically pure compounds.

An additional group of EO constituents that caught our attention was a series of compounds with a similar mass spectral fragmentation pattern, one of them an already well-known natural product, (2*E*,6*Z*,8*E*)-*N*-isobutyldeca-2,6,8-trienamide (syn. spilanthol) [3]. Chromatographic separation of the EO sample yielded a polar fraction (F7 from Table 1), containing more than 50% of spilanthol and related compounds. A comparison of the obtained NMR data of fraction F7 (Figures S51 and S52) with the literature data confirmed the identity of spilanthol [14,15]. The specific MS fragmentation pattern of the four additionally detected constituents (base peak at *m*/*z* 81 and intense peaks at 141, 126, and 98) indicated analogous unsaturated amides [3]. Unfortunately, based on the differences in their RI values and the mentioned similarity in the MS fragmentation pattern (Figure S50), we can only conclude that the detected constituents represent regio-isomers and/or stereoisomers of *N*-isobutyldeca-2,6,8-trienamide. Some of the identified isomers were already detected as constituents of *A. oleracea* ethanolic extract, however, without determining the configurations of the double-bonds [16].

The generated PIC chromatogram of fraction F7 that showed changes in the *m*/*z* 81 ion current allowed us to detect six spilanthol-related amides. The presence of the ion peak at *m*/*z* 155 (C_9_H_17_NO^+^), compared to *m*/*z* 141 (C_8_H_15_NO^+^) from spilanthol, suggested that the detected constituents could be isomeric *N*-pentyl amides of spilanthic acid (Figure S53). A comparison of the RI data of the detected constituents and ones for spilanthol suggested that one of the constituents, at RI = 2014 (Table 1), represents (2*E*,6*Z*,8*E*)-*N*-(2-methylbutyl)deca-2,6,8-trienamide (syn. homospilanthol) [17], whereas other constituents could represent regio-isomers and/or stereoisomers of homospilanthol. The identification of acmellonate analogs and spilanthol isomers further highlights the EO’s complexity and potential for developing new insecticidal and antimicrobial agents.

## 3. Materials and Methods

### 3.1. General Experimental Procedures

All solvents (*n*-hexane, diethyl ether (Et_2_O), tetrahydrofuran (THF), dichloromethane (DCM), and deuterated chloroform (CDCl_3_); HPLC grade), anhydrous MgSO_4_, sulfuric acid, diisopropylamine, *n*-butyllithium 2.5 M solution in hexanes (*n*-BuLi), trimethylsilyl chloride (TMSCl), *m*-chloroperoxybenzoic acid (m-CPBA), 2-undecanone, 2-dodecanone, 2-tridecanone, dimethyl disulfide (DMDS), iodide, 4-dimethylaminopyridine (DMAP), *N*,*N*’-dicyclohexylcarbodiimide (DCC), and corresponding acids (2-methylpropanoic, 2-methylbutanoic, 3-methylbutanoic, senecioic (3-methyl-2-butenoic), tiglic ((*E*)-2-methyl-2-butenoic), and angelic ((*Z*)-2-methyl-2-butenoic acid)), were purchased from Sigma-Aldrich (St Louis, MO, USA). Two hydrocarbon mixtures (Sigma-Aldrich (St Louis, MO, USA)), ranging from heptane to icosane and from heneicosane to tetracontane, were used to determine the retention indices.

Silica gel 60, particle size distribution 40–63 mm, was used for dry-flash chromatography, whereas precoated Al silica gel plates (Merck (Darmstadt, Germany), Kieselgel 60 F_254_, 0.2 mm) were used for analytical TLC analyses. The spots on TLC were visualized by UV light (254 nm) and by spraying with 50% (*v*/*v*) aq. H_2_SO_4_ or 10% (*w*/*v*) ethanolic solution of phosphomolybdic acid, followed by 10 min of heating at 110 °C. IR measurements (ATR-attenuated total reflectance) were carried out using a Thermo Nicolet model 6700 FTIR instrument (Waltham, MA, USA).

### 3.2. Plant Material

Leaves and inflorescences of *Acmella oleracea* (L.) R. K. Jansen were collected in April 2019 from the district of Fazendinha (S 0°02′30.40″/W 5106′37.5″), Macapá City, Amapá State, Brazil. A voucher specimen was deposited in the Herbarium of the Institute of Scientific and Technological Research of Amapá–IEPA under the identification number HAMAB-020058. The identity of the plant material was confirmed by a trained botanist, the custodian of the mentioned herbarium. The request for permission to access the material was registered with the Genetic Heritage Management Council (SISGEN) under the number A7C63F0.

### 3.3. Hydro-Distillation and Chromatographic Fractionation of A. oleracea Essential Oil

The fresh leaves and inflorescences (three batches, ca. 300 g each) were crushed and submitted to hydro-distillation in a modified Clevenger-type apparatus for 2 h with the addition of 5 mL of hexane. The obtained EO were separated by extraction and dried with anhydrous sodium sulfate. The solvent was evaporated under a gentle stream of nitrogen at room temperature and stored at around 8 °C before analysis by GC-MS. A portion of the EO (300 mg) was subjected to dry-flash chromatography, resulting in 7 different fractions in total (the mass of the fractions was 24, 41, 33, 1, 5, 26, and 30 mg). A gradient of hexane-diethyl ether, from 100:0 to 0:100 (*v*/*v*), was employed for the chromatography, and the mentioned fractions were immediately analyzed by GC and GC-MS upon solvent removal in vacuo.

### 3.4. Component Identification

Essential oil constituents were identified by comparison of their linear retention indices (relative to the mentioned homologous series of *n*-alkanes on a DB-5MS column) with literature values, their mass spectra with those of authentic standards, as well as those from Wiley 11, NIST17, MassFinder 2.3, and a homemade MS library, with the spectra corresponding to pure substances, NMR analysis of isolated compounds, and wherever possible, by co-injection with an authentic sample. Additionally, a sample of the selected chromatographic fraction was subjected to derivatization reactions with dimethyl disulfide (DMDS), described in detail below, and afterwards to additional GC-MS analyses.

### 3.5. Gas Chromatography–Mass Spectrometry (GC-MS) Analyses

The GC-MS analyses (three repetitions) of the obtained samples were carried out using a Hewlett–Packard 6890N gas chromatograph equipped with a fused-silica capillary column (DB-5MS (5% diphenylpolysiloxane and 95% dimethylpolysiloxane, 30 m × 0.25 mm, film thickness 0.25 μm) and coupled with a 5975B mass-selective detector from the same company. The injector and interface were operated at 250 °C and 300 °C or 320 °C, respectively. Two temperature programs were used. Program 1 was used for the EO sample and EO fractions: the oven temperature was raised from 70 °C to 290 °C at a heating rate of 5 °C/min, and the program ended with an isothermal period of 10 min. Program 2 was for DMDS derivatized samples: oven temperature was raised from 70 to 315 °C at a heating rate of 5 °C/min, and then isothermally held for 30 min. As a carrier, gas helium at 1.0 mL/min was used. The samples, 1.0 μL of the Et_2_O solutions of the esters, were injected in a pulsed split mode (the flow was 1.5 mL/min for the first 0.5 min and then set to 1.0 mL/min throughout the remainder of the analysis; split ratio 40:1). MS conditions were as follows: ionization voltage of 70 eV, acquisition mass range of 35–650, and scan time of 0.32 s. The linear retention indices were determined relative to the retention times of C_7_–C_33_ *n*-alkanes [18].

### 3.6. NMR Measurements

The ^1^H (including ^1^H NMR-selective homonuclear decoupling experiments), ^13^C (with and without heteronuclear decoupling) nuclear magnetic resonance (NMR) spectra, distortion less enhancement by polarization transfer (DEPT90 and DEPT135), and 2D (NOESY, and gradient ^1^H–^1^H COSY, HSQC, and HMBC) NMR spectra were recorded on a Bruker Avance III 400 MHz NMR spectrometer (Fällanden, Switzerland; ^1^H at 400 MHz, ^13^C at 101 MHz) equipped with a 5–11 mm dual ^13^C/^1^H probe head. All NMR spectra were measured at 25 °C in CDCl_3_ with tetramethylsilane (TMS) as an internal standard. Chemical shifts were reported in ppm (*δ*) and referenced to TMS (*δ*_H_ = 0 ppm) in ^1^H NMR spectra and/or to solvent protons (deuterated chloroform: *δ*_H_ = 7.26 ppm and *δ*_C_ = 77.16 ppm) in ^13^C and heteronuclear 2D spectra. The samples were dissolved in 1 mL of the solvent, and 0.7 mL of the solutions were transferred into a 5 mm Wilmad, 528-TR-7 NMR tube. The acquired NMR experiments, both 1D and 2D, were recorded using standard Bruker built-in pulse sequences.

### 3.7. Synthesis of 1-hydroxyundecan-2-one, 1-hydroxydodecan-2-one, and 1-hydroxytridecan-2-one

A solution of diisopropylamine (8.33 mmol; 1.1 eq) in freshly dried THF (10 mL) was vigorously stirred under nitrogen in the bath with the cooled acetone, and *n*-BuLi (3.60 mL) was added dropwise through the septum and left for 10 min at −78 °C and an additional 10 min at 0 °C. Then, a solution of the 2-tridecanone (7.57 mmol; 1.5 g) in the THF (10 mL) was added dropwise through the septum, and 10 min later, trimethylsilyl chloride (1.05 mL; 1.1 eq) and stirred at ambient temperature overnight. The solvent was evaporated, and the remaining slurry was diluted in CH_2_Cl_2_ (10 mL), and m-CPBA (2.05 g in 10 mL CH_2_Cl_2_) was added dropwise and stirred at ambient temperature overnight. A solution of the sulfuric acid at 15 mL (10%, *v*/*v*) was added and stirred for 2 h. The organic layers were separated, dried over anhydrous MgSO_4_, and concentrated under reduced pressure to yield a crude mixture that was further fractionated by dry-flash chromatography on SiO_2_, using mixtures of the increasing polarity of hexane and Et_2_O as the eluent to yield pure 1-hydroxytridecan-2-one (1.03 g). Using the same synthetic procedure, 2-undecanone and 2-dodecanone, 1-hydroxyundecan-2-one, and 1-hydroxydodecan-2-one were synthesized. The spectral data (NMR and MS) and assignments of ^1^H and ^13^C signals for the synthesized compounds are provided below and in the Supplementary Materials (Figures S3–S43).

1-Hydroxyundecan-2-one (**1**): Yield: 64%; RI = 1469 (DB-5MS column); ^1^H NMR (400 MHz, CDCl_3_) 4.25 (2H, singlet, H-1), 2.41 (2H, triplet, *J* = 7.5 Hz, H-3), 1.63 (2H, quintet, *J* = 7.2 Hz, H-4), 1.33–1.24 (12H, overlapping peaks, H-5–H-10), 0.87 (3H, pseudo triplet, *J* = 7.1 Hz, H-11); ^13^C NMR (101 MHz, CDCl_3_) 209.95 (C-2), 68.06 (C-1), 38.46 (C-3), 31.86, 29.38, 29.35, 29.26, 29.15, and 22.67 (C-5–C-10), 23.26 (C-4), 14.12 (C-11); MS (EI), *m*/*z* (%) 156 (11), 155 (100), 95 (21), 85 (29), 83 (5), 81 (16), 71 (47), 69 (12), 67 (8), 58 (4), 57 (48), 56 (7), 55 (30), 53 (4), 43 (67), 42 (11), 41 (47).

1-Hydroxydodecan-2-one (**2**): Yield: 68%; RI = 1570 (DB-5MS column); ^1^H NMR (400 MHz, CDCl_3_) 4.25 (2H, singlet, H-1), 2.41 (2H, triplet, *J* = 7.5 Hz, H-3), 1.63 (2H, quintet, *J* = 7.2 Hz, H-4), 1.34–1.22 (14H, overlapping peaks, H-5–H-11), 0.87 (3H, pseudo triplet, *J* = 7.0 Hz, H-12); ^13^C NMR (101 MHz, CDCl_3_) 210.00 (C-2), 68.06 (C-1), 38.46 (C-3), 31.89, 29.55, 29.43, 29.30, 29.21, and 22.69 (C-5–C-11), 23.75 (C-4), 14.12 (C-12); MS (EI), *m*/*z* (%) 170 (12), 169 (100), 109 (10), 97 (4), 95 (29), 85 (29), 83 (10), 81 (11), 71 (29), 69 (13), 67 (9), 58 (4), 57 (64), 56 (8), 55 (33), 43 (62), 42 (11), 41 (45).

1-Hydroxytridecan-2-one (**3**): Yield: 65%; RI = 1671 (DB-5MS column); ^1^H NMR (400 MHz, CDCl_3_) 4.25 (2H, singlet, H-1), 2.41 (2H, triplet, *J* = 7.5 Hz, H-3), 1.63 (2H, quintet, *J* = 7.1 Hz, H-4), 1.35–1.21 (16H, overlapping peaks, H-5–H-12), 0.87 (3H, pseudo triplet, *J* = 7.0 Hz, H-13); ^13^C NMR (101 MHz, CDCl_3_) 209.95 (C-2), 68.08 (C-1), 38.46 (C-3), 31.91, 29.59, 29.42, 29.33, 29.30, 29.22, and 22.69 (C-5–C-12), 23.75 (C-4), 14.12 (C-13); MS (EI), *m*/*z* (%) 184 (13), 183 (100), 109 (16), 97 (8), 95 (25), 85 (22), 83 (14), 81 (11), 71 (34), 69 (12), 67 (9), 58 (5), 57 (76), 56 (8), 55 (35), 43 (59), 42 (10), 41 (43).

### 3.8. Synthesis of Esters

A solution of the appropriate alcohol (1-hydroxyundecan-2-one (**1**), 1-hydroxydodecan-2-one (**2**), and 1-hydroxytridecan-2-one (**3**)), carboxylic acid (1.1 eq; 2-methylpropanoic (**a**), 2-methylbutanoic (**b**), 3-methylbutanoic (**c**), angelic acid (**d**), tiglic (**e**), and senecioic (**f**)), DMAP (0.3 eq), and DCC (1.1 eq) in 30 mL of dry CH_2_Cl_2_ was stirred overnight, at room temperature, in a round-bottom flask equipped with a CaCl_2_ guard tube [19,20]. The precipitated urea was filtered off and the filtrate was concentrated under a vacuum. The resulting residue was purified by dry-flash chromatography using mixtures of hexane and Et_2_O of increasing polarity for elution. Esters were washed from the column with 10% (*v*/*v*) Et_2_O in hexane. The purity of the ester fractions was checked by TLC and GC-MS. The yield of the esterification step, spectral data (NMR, MS, and IR), and assignments of ^1^H and ^13^C signals for the synthesized esters are presented below and in the Supplementary Materials.

2-Oxoundecyl isobutyrate (**1a**): Yield: 66%; RI = 1769 (DB-5MS column); IR (cm^−1^) 2923, 2853, 1731, 1468, 1415, 1386, 1252, 1188, 1156, 1086, 897, 825, 721; ^1^H NMR (400 MHz, CDCl_3_) 4.64 (2H, singlet, H-1), 2.68 (1H, heptet, *J* = 7.0 Hz, H-2′), 2.41 (2H, triplet, *J* = 7.4 Hz, H-3), 1.60 (2H, quintet, *J* = 7.4 Hz, H-4), 1.34–1.25 (12H, overlapping peaks, H-5–H-10), 1.23 (6H, doublet, *J* = 7.0 Hz, H-3′ and H-4′), 0.88 (3H, pseudo triplet, *J* = 6.8 Hz, H-10); ^13^C NMR (101 MHz, CDCl_3_) 204.24 (C-2), 176.40 (C-1′), 67.77 (C-1), 38.81 (C-3), 33.72 (C-2′), 31.84, 29.37, 29.34, 29.24, 29.14, and 22.66 (C-5–C-10), 23.25 (C-4), 18.93 (C-3′ and C-4′), 14.10 (C-11); MS (EI), *m*/*z* (%) 184 (6), 183 (42), 144 (21), 109 (11), 98 (5), 97 (6), 95 (17), 85 (17), 83 (10), 81 (7), 72 (5), 71 (100), 70 (5), 69 (9), 67 (6), 57 (47), 56 (6), 55 (20), 43 (86), 42 (10), 41 (33).

2-Oxoundecyl 2-methylbutanoate (**1b**): Yield: 68%; RI = 1860 (DB-5MS column); IR (cm^−1^) 2922, 2854, 1730, 1461, 1415, 1378, 1262, 1237, 1179, 1151, 1083, 1015, 871, 752, 721; ^1^H NMR (400 MHz, CDCl_3_) 4.65 (2H, singlet, H-1), 2.50 (1H, sextet, *J* = 7.0 Hz, H-2′), 2.41 (2H, triplet, *J* = 7.4 Hz, H-3), 1.75 (1H, pseudo doublet of quintets, *J* = −13.6, 7.4 Hz, H-3′a), 1.66–1.44 (3H, overlapping peaks, H-4 and H-3′b), 1.33–1.23 (12H, overlapping peaks, H-5–H-10), 1.21 (3H, doublet, *J* = 7.0 Hz, H-5′), 0.96 (3H, triplet, *J* = 7.4 Hz, H-4′), 0.87 (3H, pseudo triplet, *J* = 7.0 Hz, H-10); ^13^C NMR (101 MHz, CDCl_3_) 204.21 (C-2), 176.02 (C-1′), 67.71 (C-1), 40.79 (C-2′), 38.86 (C-3), 31.86, 29.38, 29.35, 29.26, 29.15, and 22.67 (C-5–C-10), 26.73 (C-3′), 23.26 (C-4), 16.61 (C-5′), 14.11 (C-11), 11.58 (C-4′); MS (EI), *m*/*z* (%) 158 (18), 156 (6), 155 (55), 95 (14), 86 (6), 85 (100), 81 (11), 71 (32), 69 (8), 58 (5), 57 (99), 56 (9), 55 (20), 43 (37), 42 (10), 41 (39).

2-Oxoundecyl 3-methylbutanoate (**1c**): Yield: 70%; RI = 1863 (DB-5MS column); IR (cm^−1^) 2923, 2853, 1731, 1468, 1415, 1370, 1293, 1255, 1184, 1165, 1119, 1029, 831, 721; ^1^H NMR (400 MHz, CDCl_3_) 4.65 (2H, singlet, H-1), 2.41 (2H, triplet, *J* = 7.4 Hz, H-3), 2.31 (2H, doublet, *J* = 7.1 Hz, H-2′), 2.21–2.11 (1H, multiplet, H-3′), 1.60 (2H, quintet, *J* = 7.4 Hz, H-4), 1.34–1.22 (12H, overlapping peaks, H-5–H-10), 1.00 (6H, doublet, *J* = 6.7 Hz, H-4′ and H-5′), 0.87 (3H, pseudo triplet, *J* = 7.0 Hz, H-11); ^13^C NMR (101 MHz, CDCl_3_) 204.11 (C-2), 172.36 (C-1′), 67.73 (C-1), 42.90 (C-2′), 38.86 (C-3), 31.86, 29.39, 29.34, 29.25, 29.15 and 22.67 (C-5–C-10), 25.71 (C-3′), 23.29 (C-4), 22.40 (C-4′ and C-5′), 14.11 (C-11); MS (EI), *m*/*z* (%) 158 (14), 156 (7), 155 (59), 95 (13), 86 (6), 85 (100), 81 (10), 71 (30), 69 (11), 58 (6), 57 (61), 56 (6), 55 (18), 43 (37), 42 (10), 41 (35).

2-Oxoundecyl (Z)-2-methyl-2-butenoate (syn. 2-oxoundecyl angelate) (**1d**): Yield: 41%; RI = 1908 (DB-5MS column); IR (cm^−1^) 2923, 2854, 1717, 1650, 1456, 1414, 1379, 1355, 1230, 1151, 1083, 1047, 846, 754; ^1^H NMR (400 MHz, CDCl_3_) 6.16 (1H, quartet of quartets, *J* = 7.3, 1.5 Hz, H-3′), 4.72 (2H, singlet, H-1), 2.44 (2H, triplet, *J* = 7.4 Hz, H-3), 2.02 (3H, doublet of quartets, *J* = 7.3, 1.5 Hz, H-4′), 1.95 (3H, pseudo quintet, *J* = 1.5 Hz, H-5′), 1.62 (2H, quintet, *J* = 7.4 Hz, H-4), 1.34–1.23 (12H, overlapping peaks, H-5–H-10), 0.87 (3H, pseudo triplet, *J* = 7.0 Hz, H-11); ^13^C NMR (101 MHz, CDCl_3_) 204.36 (C-2), 167.07 (C-1′), 139.55 (C-3′), 127.01 (C-2′), 67.74 (C-1), 38.90 (C-3), 31.86, 29.39, 29.36, 29.26, 29.17, and 22.67 (C-5–C-10), 23.28 (C-4), 20.51 (C-5′), 15.87 (C-4′), 14.11 (C-11); MS (EI), *m*/*z* (%) 156 (10), 155 (17), 95 (6), 85 (10), 83 (100), 82 (27), 71 (16), 57 (14), 55 (44), 53 (5), 43 (20), 41 (12).

2-Oxoundecyl (E)-2-methyl-2-butenoate (syn. 2-oxoundecyl tiglate) (**1e**): Yield: 69%; RI = 1954 (DB-5MS column); IR (cm^−1^) 2923, 2854, 1713, 1652, 1465, 1415, 1379, 1255, 1145, 1128, 1078, 732; ^1^H NMR (400 MHz, CDCl_3_) 6.98 (1H, quartet of quartets, *J* = 7.0, 1.4 Hz, H-3′), 4.70 (2H, singlet, H-1), 2.43 (2H, triplet, *J* = 7.4 Hz, H-3), 1.88 (3H, pseudo quintet, *J* = 1.4 Hz, H-5′), 1.83 (3H, doublet of quartets, *J* = 7.0, 1.4 Hz, H-4′), 1.62 (2H, quintet, *J* = 7.4 Hz, H-4), 1.33–1.21 (12H, overlapping peaks, H-5–H-10), 0.87 (3H, pseudo triplet, *J* = 7.0 Hz, H-11); ^13^C NMR (101 MHz, CDCl_3_) 204.74 (C-2), 167.24 (C-1′), 138.78 (C-3′), 127.79 (C-2′), 68.06 (C-1), 38.89 (C-3), 31.85, 29.39, 29.36, 29.25, 29.17, and 22.67 (C-5–C-10), 23.30 (C-4), 14.50 (C-4′), 14.11 (C-11), 12.05 (C-5′); MS (EI), *m*/*z* (%) 156 (16), 155 (14), 95 (6), 85 (10), 84 (6), 83 (100), 71 (15), 57 (13), 55 (39), 53 (5), 43 (18).

2-Oxoundecyl 3-methyl-2-butenoate (syn. 2-oxoundecyl senecioate) (**1f**): Yield: 70%; RI = 1948 (DB-5MS column); IR (cm^−1^) 2923, 2854, 1720, 1650, 1445, 1378, 1348, 1274, 1225, 1136, 1075, 1030, 849, 723; ^1^H NMR (400 MHz, CDCl_3_) 5.80 (1H, heptet, *J* = 1.3 Hz, H-2′), 4.66 (2H, singlet, H-1), 2.43 (2H, triplet, *J* = 7.5 Hz, H-3), 2.18 (3H, doublet, *J* = 1.3 Hz, H-5′), 1.93 (3H, doublet, *J* = 1.3 Hz, H-4′), 1.60 (2H, quintet, *J* = 7.5 Hz, H-4), 1.33–1.23 (12H, overlapping peaks, H-5–H-10), 0.87 (3H, pseudo triplet, *J* = 7.0 Hz, H-11); ^13^C NMR (101 MHz, CDCl_3_) 204.87 (C-2), 165.59 (C-1′), 158.98 (C-3′), 114.91 (C-2′), 67.35 (C-1), 38.87 (C-3), 31.86, 29.39, 29.34, 29.25, 29.15, and 22.67 (C-5–C-10), 27.54 (C-4′), 23.30 (C-4), 20.41 (C-5′), 14.12 (C-11); MS (EI), *m*/*z* (%) 156 (7), 155 (5), 84 (6), 83 (100), 71 (5), 57 (5), 55 (13), 43 (9).

2-Oxododecyl isobutyrate (**2a**): Yield: 68%; RI = 1869 (DB-5MS column); IR (cm^−1^) 2922, 2853, 1731, 1468, 1415, 1386, 1252, 1188, 1157, 1081, 897, 842, 756, 721; ^1^H NMR (400 MHz, CDCl_3_) 4.64 (2H, singlet, H-1), 2.68 (1H, heptet, *J* = 7.0 Hz, H-2′), 2.41 (2H, triplet, *J* = 7.4 Hz, H-3), 1.60 (2H, quintet, *J* = 7.4 Hz, H-4), 1.33–1.24 (14H, overlapping peaks, H-5–H-11), 1.23 (6H, doublet, *J* = 7.0 Hz, H-3′ and H-4′), 0.88 (3H, pseudo triplet, *J* = 6.8 Hz, H-12); ^13^C NMR (101 MHz, CDCl_3_) 204.26 (C-2), 176.42 (C-1′), 67.78 (C-1), 38.83 (C-3), 33.73 (C-2′), 31.89, 29.55, 29.42, 29.35, 29.30, 29.15, and 22.68 (C-5–C-11), 23.26 (C-4), 18.94 (C-3′ and C-4′), 14.12 (C-12); MS (EI), *m*/*z* (%) 200 (3), 170 (8), 169 (62), 144 (24), 109 (8), 95 (24), 85 (25), 83 (8), 81 (9), 72 (5), 71 (100), 69 (9), 57 (41), 55 (19), 43 (88), 42 (11), 41 (36).

2-Oxododecyl 2-methylbutanoate (**2b**): Yield: 67%; RI = 1961 (DB-5MS column); IR (cm^−1^) 2923, 2854, 1731, 1461, 1415, 1378, 1262, 1237, 1179, 1151, 1085, 1029, 845, 754, 721; ^1^H NMR (400 MHz, CDCl_3_) 4.65 (2H, singlet, H-1), 2.50 (1H, sextet, *J* = 7.0 Hz, H-2′), 2.41 (2H, triplet, *J* = 7.5 Hz, H-3), 1.75 (1H, pseudo doublet of quintets, *J* = −13.6, 7.4 Hz, H-3′a), 1.64–1.46 (3H, overlapping peaks, H-4 and H-3′b), 1.33–1.23 (14H, overlapping peaks, H-5–H-11), 1.21 (3H, doublet, *J* = 7.0 Hz, H-5′), 0.96 (3H, triplet, *J* = 7.4 Hz, H-4′), 0.88 (3H, pseudo triplet, *J* = 7.0 Hz, H-12); ^13^C NMR (101 MHz, CDCl_3_) 204.23 (C-2), 176.03 (C-1′), 67.71 (C-1), 40.79 (C-2′), 38.86 (C-3), 31.89, 29.55, 29.43, 29.35, 29.30, 29.16, and 22.68 (C-5–C-11), 26.73 (C-3′), 23.26 (C-4), 16.61 (C-5′), 14.12 (C-12), 11.56 (C-4′); MS (EI), *m*/*z* (%) 170 (6), 169 (51), 158 (20), 109 (7), 95 (19), 86 (6), 85 (100), 83 (7), 81 (7), 71 (19), 69 (8), 58 (5), 57 (100), 56 (9), 55 (19), 43 (30), 42 (8), 41 (35).

2-Oxododecyl 3-methylbutanoate (**2c**): Yield: 71%; RI = 1964 (DB-5MS column); IR (cm^−1^) 2955, 2923, 2853, 1732, 1466, 1415, 1370, 1293, 1250, 1184, 1166, 1119, 1029, 831, 721; ^1^H NMR (400 MHz, CDCl_3_) 4.65 (2H, singlet, H-1), 2.41 (2H, triplet, *J* = 7.4 Hz, H-3), 2.31 (2H, doublet, *J* = 7.0 Hz, H-2′), 2.24–2.07 (1H, multiplet, H-3′), 1.60 (2H, quintet, *J* = 7.4 Hz, H-4), 1.35–1.22 (14H, overlapping peaks, H-5–H-11), 1.00 (6H, doublet, *J* = 6.4 Hz, H-4′ and H-5′), 0.88 (3H, pseudo triplet, *J* = 7.0 Hz, H-12); ^13^C NMR (101 MHz, CDCl_3_) 204.23 (C-2), 172.34 (C-1′), 67.71 (C-1), 42.90 (C-2′), 38.86 (C-3), 31.89, 29.55, 29.43, 29.35, 29.30, 29.16, and 22.68 (C-5–C-11), 25.71 (C-3′), 23.26 (C-4), 22.40 (C-4′ and C-5′), 14.12 (C-12); MS (EI), *m*/*z* (%) 200 (5), 170 (7), 169 (58), 158 (17), 109 (7), 95 (18), 86 (6), 85 (100), 83 (7), 81 (6), 71 (18), 69 (11), 57 (66), 56 (6), 55 (18), 43 (34), 42 (9), 41 (32).

2-Oxododecyl (Z)-2-methyl-2-butenoate (syn. 2-oxododecyl angelate) (**2d**): Yield: 41%; RI = 2009 (DB-5MS column); IR (cm^−1^) 2923, 2854, 1718, 1654, 1456, 1414, 1379, 1356, 1231, 1151, 1083, 1048, 846, 751; ^1^H NMR (400 MHz, CDCl_3_) 6.16 (1H, quartet of quartets, *J* = 7.2, 1.5 Hz, H-3′), 4.72 (2H, singlet, H-1), 2.41 (2H, triplet, *J* = 7.5 Hz, H-3), 2.02 (3H, doublet of quartets, *J* = 7.2, 1.5 Hz, H-4′), 1.95 (3H, pseudo quintet, *J* = 1.5 Hz, H-5′), 1.62 (2H, quintet, *J* = 7.5 Hz, H-4), 1.38–1.21 (14H, overlapping peaks, H-5–H-11), 0.87 (3H, pseudo triplet, *J* = 7.0 Hz, H-11); ^13^C NMR (101 MHz, CDCl_3_) 204.38 (C-2), 167.07 (C-1′), 139.56 (C-3′), 127.00 (C-2′), 67.74 (C-1), 38.91 (C-3), 31.89, 29.55, 29.43, 29.35, 29.31, 29.16, and 22.69 (C-5–C-11), 23.27 (C-4), 20.51 (C-5′), 15.87 (C-4′), 14.12 (C-12); MS (EI), *m*/*z* (%) 169 (18), 156 (13), 95 (9), 85 (10), 83 (100), 82 (26), 71 (10), 57 (17), 55 (41), 43 (18), 41 (15), 39 (6).

2-Oxododecyl (E)-2-methyl-2-butenoate (syn. 2-oxododecyl tiglate) (**2e**): Yield: 69%; RI = 2055 (DB-5MS column); IR (cm^−1^) 2923, 2854, 1712, 1654, 1464, 1415, 1379, 1255, 1145, 1129, 1079, 731; ^1^H NMR (400 MHz, CDCl_3_) 6.98 (1H, quartet of quartets, *J* = 7.1, 1.4 Hz, H-3′), 4.70 (2H, singlet, H-1), 2.43 (2H, triplet, *J* = 7.4 Hz, H-3), 1.88 (3H, pseudo quintet, *J* = 1.4 Hz, H-5′), 1.83 (3H, doublet of quartets, *J* = 7.1, 1.4 Hz, H-4′), 1.61 (2H, quintet, *J* = 7.4 Hz, H-4), 1.34–1.21 (14H, overlapping peaks, H-5–H-11), 0.87 (3H, pseudo triplet, *J* = 7.0 Hz, H-12); ^13^C NMR (101 MHz, CDCl_3_) 204.72 (C-2), 167.23 (C-1′), 138.77 (C-3′), 127.79 (C-2′), 68.06 (C-1), 38.88 (C-3), 31.89, 29.55, 29.44, 29.36, 29.31, 29.17, and 22.69 (C-5–C-11), 23.30 (C-4), 14.50 (C-4′), 14.12 (C-12), 12.05 (C-5′); MS (EI), *m*/*z* (%) 169 (14), 156 (17), 95 (8), 85 (9), 84 (6), 83 (100), 71 (9), 57 (16), 55 (34), 43 (15), 41 (11), 39 (5).

2-Oxododecyl 3-methyl-2-butenoate (syn. 2-oxododecyl senecioate) (**2f**): Yield: 71%; RI = 2049 (DB-5MS column); IR (cm^−1^) 2923, 2854, 1718, 1654, 1444, 1378, 1348, 1274, 1225, 1136, 1074, 1032, 851, 723; ^1^H NMR (400 MHz, CDCl_3_) 5.80 (1H, heptet, *J* = 1.4 Hz, H-2′), 4.66 (2H, singlet, H-1), 2.43 (2H, triplet, *J* = 7.5 Hz, H-3), 2.18 (3H, doublet, *J* = 1.4 Hz, H-5′), 1.93 (3H, doublet, *J* = 1.4 Hz, H-4′), 1.61 (2H, quintet, *J* = 7.5 Hz, H-4), 1.35–1.21 (14H, overlapping peaks, H-5–H-11), 0.88 (3H, pseudo triplet, *J* = 6.8 Hz, H-12); ^13^C NMR (101 MHz, CDCl_3_) 204.88 (C-2), 165.60 (C-1′), 158.98 (C-3′), 114.91 (C-2′), 67.36 (C-1), 38.87 (C-3), 31.90, 29.56, 29.44, 29.35, 29.31, 29.16, and 22.69 (C-5–C-11), 27.55 (C-4′), 23.31 (C-4), 20.42 (C-5′), 14.13 (C-12); MS (EI), *m*/*z* (%) 169 (5), 156 (8), 84 (6), 83 (100), 57 (6), 55 (13), 43 (8).

2-Oxotridecyl isobutyrate (**3a**): Yield: 65%; RI = 1969 (DB-5MS column); IR (cm^−1^) 2923, 2853, 1731, 1468, 1415, 1386, 1252, 1188, 1156, 1086, 897, 825, 720; ^1^H NMR (400 MHz, CDCl_3_) 4.64 (2H, singlet, H-1), 2.68 (1H, heptet, *J* = 7.0 Hz, H-2′), 2.41 (2H, triplet, *J* = 7.4 Hz, H-3), 1.60 (2H, quintet, *J* = 7.4 Hz, H-4), 1.32–1.23 (16H, overlapping peaks, H-5–H-12), 1.23 (6H, doublet, *J* = 7.0 Hz, H-3′ and H-4′), 0.87 (3H, pseudo triplet, *J* = 7.0 Hz, H-13); ^13^C NMR (101 MHz, CDCl_3_) 204.26 (C-2), 176.43 (C-1′), 67.78 (C-1), 38.83 (C-3), 33.73 (C-2′), 31.91, 29.60, 29.42, 29.35, 29.33, 29.15, and 22.69 (C-5–C-12), 23.26 (C-4), 18.95 (C-3′ and C-4′), 14.13 (C-13); MS (EI), *m*/*z* (%) 183 (42), 144 (21), 109 (11), 97 (8), 97 (6), 95 (18), 85 (17), 83 (10), 81 (9), 71 (100), 69 (10), 57 (47), 55 (23), 43 (91), 41 (38).

2-Oxotridecyl 2-methylbutanoate (**3b**): Yield: 67%; RI = 2062 (DB-5MS column); IR (cm^−1^) 2923, 2853, 1731, 1461, 1415, 1378, 1262, 1236, 1178, 1150, 1087, 1029, 868, 720; ^1^H NMR (400 MHz, CDCl_3_) 4.65 (2H, singlet, H-1), 2.50 (1H, sextet, *J* = 7.0 Hz, H-2′), 2.41 (2H, triplet, *J* = 7.4 Hz, H-3), 1.75 (1H, pseudo doublet of quintets, *J* = −13.5, 7.4 Hz, H-3′a), 1.58–1.46 (3H, overlapping peaks, H-4 and H-3′b), 1.33–1.23 (16H, overlapping peaks, H-5–H-12), 1.21 (3H, doublet, *J* = 7.0 Hz, H-5′), 0.96 (3H, triplet, *J* = 7.5 Hz, H-4′), 0.87 (3H, pseudo triplet, *J* = 7.0 Hz, H-13); ^13^C NMR (101 MHz, CDCl_3_) 204.22 (C-2), 176.03 (C-1′), 67.71 (C-1), 40.79 (C-2′), 38.86 (C-3), 31.91, 29.60, 29.43, 29.35, 29.34, 29.16, and 22.70 (C-5–C-12), 26.73 (C-3′), 23.26 (C-4), 16.62 (C-5′), 14.13 (C-13), 11.58 (C-4′); MS (EI), *m*/*z* (%) 184 (4), 183 (34), 158 (17), 109 (9), 97 (6), 95 (14), 86 (5), 85 (89), 83 (9), 81 (7), 71 (20), 69 (8), 58 (6), 57 (100), 56 (9), 55 (21), 43 (31), (10), 41 (34).

2-Oxotridecyl 3-methylbutanoate (**3c**): Yield: 69%; RI = 2065 (DB-5MS column); IR (cm^−1^) 2955, 2915, 2849, 1723, 1471, 1417, 1333, 1295, 1183, 1126, 1099, 1076, 1004, 986, 877, 828, 718; ^1^H NMR (400 MHz, CDCl_3_) 4.65 (2H, singlet, H-1), 2.41 (2H, triplet, *J* = 7.4 Hz, H-3), 2.31 (2H, doublet, *J* = 7.1 Hz, H-2′), 2.24–2.06 (1H, multiplet, H-3′), 1.60 (2H, quintet, *J* = 7.4 Hz, H-4), 1.35–1.20 (16H, overlapping peaks, H-5–H-12), 1.00 (6H, doublet, *J* = 6.4 Hz, H-4′ and H-5′), 0.87 (3H, pseudo triplet, *J* = 7.0 Hz, H-13); ^13^C NMR (101 MHz, CDCl_3_) 204.09 (C-2), 172.34 (C-1′), 67.74 (C-1), 42.90 (C-2′), 38.39 (C-3), 31.92, 29.60, 29.43, 29.34, 29.16, and 22.69 (C-5–C-12), 25.71 (C-3′), 23.30 (C-4), 22.40 (C-4′ and C-5′), 14.12 (C-13); MS (EI), *m*/*z* (%) 214 (5%), 184 (6), 183 (49), 158 (18), 109 (10), 97 (5), 95 (15), 86 (6), 85 (100), 83 (10), 71 (19), 69 (10), 57 (71), 56 (7), 55 (19), 43 (33), 42 (8), 41 (31).

2-Oxotridecyl (Z)-2-methyl-2-butenoate (syn. 2-oxotridecyl angelate) (**3d**): Yield: 39%; RI = 2110 (DB-5MS column); IR (cm^−1^) 2953, 2916, 2850, 1714, 1464, 1408, 1384, 1360, 1232, 1157, 1128, 1100, 1086, 1045, 1003, 974, 855, 842, 757, 718; ^1^H NMR (400 MHz, CDCl_3_) 6.16 (1H, quartet of quartets, *J* = 7.3, 1.5 Hz, H-3′), 4.72 (2H, singlet, H-1), 2.44 (2H, triplet, *J* = 7.4 Hz, H-3), 2.02 (3H, doublet of quartets, *J* = 7.3, 1.5 Hz, H-4′), 1.95 (3H, pseudo quintet, *J* = 1.5 Hz, H-5′), 1.62 (2H, quintet, *J* = 7.4 Hz, H-4), 1.33–1.23 (16H, overlapping peaks, H-5–H-12), 0.87 (3H, pseudo triplet, *J* = 7.0 Hz, H-13); ^13^C NMR (101 MHz, CDCl_3_) 204.33 (C-2), 167.06 (C-1′), 139.52 (C-3′), 127.04 (C-2′), 67.75 (C-1), 38.90 (C-3), 31.93, 29.62, 29.45, 29.37, 29.35, 29.19, and 22.70 (C-5–C-12), 23.29 (C-4), 20.51 (C-5′), 15.86 (C-4′), 14.13 (C-13); MS (EI), *m*/*z* (%) 183 (12), 156 (11), 95 (7), 85 (7), 83 (100), 82 (26), 71 (11), 57 (21), 55 (40), 43 (19), 41 (15).

2-Oxotridecyl (E)-2-methyl-2-butenoate (syn. 2-oxotridecyl tiglate) (**3e**): Yield: 72%; RI = 2156 (DB-5MS column); IR (cm^−1^) 2922, 2853, 1716, 1652, 1465, 1415, 1378, 1255, 1128, 1078, 731; ^1^H NMR (400 MHz, CDCl_3_) 6.98 (1H, quartet of quartets, *J* = 7.0, 1.4 Hz, H-3′), 4.70 (2H, singlet, H-1), 2.43 (2H, triplet, *J* = 7.4 Hz, H-3), 1.88 (3H, pseudo quintet, *J* = 1.4 Hz, H-5′), 1.83 (3H, doublet of quartets, *J* = 7.0, 1.4 Hz, H-4′), 1.62 (2H, quintet, *J* = 7.4 Hz, H-4), 1.33–1.21 (16H, overlapping peaks, H-5–H-12), 0.87 (3H, pseudo triplet, *J* = 7.0 Hz, H-13); ^13^C NMR (101 MHz, CDCl_3_) 204.71 (C-2), 167.24 (C-1′), 138.75 (C-3′), 127.81 (C-2′), 68.07 (C-1), 38.89 (C-3), 31.91, 29.60, 29.44, 29.36, 29.34, 29.18, and 22.69 (C-5–C-12), 23.31 (C-4), 14.49 (C-4′), 14.12 (C-13), 12.05 (C-5′); MS (EI), *m*/*z* (%) 183 (10), 156 (16), 95 (7), 85 (8), 83 (100), 71 (11), 57 (20), 55 (36), 43 (19), 41 (16).

2-Oxotridecyl 3-methyl-2-butenoate (syn. 2-oxotridecyl senecioate) (**3f**): Yield: 73%; RI = 2150 (DB-5MS column); IR (cm^−1^) 2913, 2849, 1716, 1651, 1466, 1445, 1409, 1384, 1225, 1153, 1129, 1061, 1008, 976, 851, 768, 721; ^1^H NMR (400 MHz, CDCl_3_) 5.80 (1H, heptet, *J* = 1.3 Hz, H-2′), 4.66 (2H, singlet, H-1), 2.43 (2H, triplet, *J* = 7.5 Hz, H-3), 2.18 (3H, doublet, *J* = 1.3 Hz, H-5′), 1.93 (3H, doublet, *J* = 1.3 Hz, H-4′), 1.61 (2H, quintet, *J* = 7.5 Hz, H-4), 1.33–1.21 (16H, overlapping peaks, H-5–H-12), 0.87 (3H, pseudo triplet, *J* = 7.0 Hz, H-13); ^13^C NMR (101 MHz, CDCl_3_) 204.85 (C-2), 165.59 (C-1′), 158.93 (C-3′), 114.93 (C-2′), 67.36 (C-1), 38.87 (C-3), 31.91, 29.60, 29.44, 29.35, 29.34, 29.17, and 22.40 (C-5–C-12), 27.53 (C-4′), 23.32 (C-4), 20.41 (C-5′), 14.13 (C-13); MS (EI), *m*/*z* (%) 183 (4), 156 (8), 84 (6), 83 (100), 71 (4), 55 (13), 43 (8).

### 3.9. Dimethyl Disulfide (DMDS) Derivatization

A portion of the EO fraction was dissolved in DMDS (0.25 mL per mg of the sample), and a solution (0.05 mL per mg of the sample) of iodine in diethyl ether (60 mg/mL) was added. The mixture was stirred at room temperature overnight. Then, diethyl ether was added, and the obtained mixture was washed with 10% aq. Na_2_S_2_O_3_, dried over anhydrous MgSO_4_, and evaporated to dryness. The residue was taken up in Et_2_O and directly analyzed by GC-MS.

## 4. Conclusions

This study successfully identified 180 constituents in the EO of *Acmella oleracea*, including 12 new natural products and various acmellonate analogs. These findings not only enhanced our understanding of the chemical diversity of *A. oleracea* EO but also underscored its potential for pharmacological applications. The identification of new esters and spilanthol isomers suggests potential for developing novel bioproducts, aligning with traditional uses and paving the way for future research in drug development.

## Figures and Tables

**Table 1:** Chemical composition of the essential oil and essential oil fractions of *Acmella oleracea* (L.) R.K. Jansen from Pará-Brazil.

RI ^a^	Constituents ^b^	Samples ^c^								ID ^d^
		EO	F1	F2	F3	F4	F5	F6	F7	
786	Hexan-2-ol	tr						0.1	tr	MS, RI, CoI
885	2-Butylfuran	tr				tr	tr	0.1		MS, RI
928	α-Pinene	tr	0.1	tr						MS, RI, CoI
961	Sabinene	tr	tr	0.1						MS, RI
968	β-Pinene	0.2	0.8	0.2						MS, RI, CoI
979	β-Myrcene	0.5	0.9	1.7						MS, RI
995	*p*-Mentha-1(7),8-diene	tr	tr	tr						MS, RI
1000	Decane	tr	tr							MS, RI, CoI
1015	*p*-Cymene	tr	tr	tr						MS, RI, CoI
1019	Limonene	0.1	0.1							MS, RI, CoI
1021	β-Phellandrene	0.1	0.3	0.1						MS, RI
1025	(*Z*)-β-Ocimene	0.5	0.8							MS, RI
1028	Phenylacetaldehyde	tr						0.1	tr	MS, RI, CoI
1038	(*E*)-β-Ocimene	tr	tr							MS, RI
1048	γ-Terpinene	tr	tr							MS, RI
1089	Linalool	tr						tr		MS, RI, CoI
1120	2-Phenylethan-1-ol	tr							tr	MS, RI, CoI
1132	*trans*-Pinocarveol	tr					tr	tr	tr	MS, RI
1155	Pinocarvone	tr			tr	tr	tr			MS, RI, CoI
1158	Rosefurane epoxide	tr							tr	MS, RI
1167	Terpinen-4-ol	0.5						1.0		MS, RI, CoI
1177	Cryptone	tr						0.1	tr	MS, RI
1180	α-Terpineol	0.1							0.2	MS, RI
1187	Myrtenol	tr						tr	tr	MS, RI
1200	Dodecane	tr	tr							MS, RI, CoI
1278	Isobornyl acetate	tr			tr					MS, RI
1287	Undecan-2-one	tr				tr				MS, RI, CoI
1300	Tridecane	tr	tr							MS, RI, CoI
1321	Silphiperfol-5-ene	tr	tr							MS, RI
1329	δ-Elemene	0.3	1.4	0.9						MS, RI
1343	α-Cubebene	tr	tr	0.1						MS, RI
1371	α-Copaene	tr	0.4	0.1						MS, RI
1374	(*E*)-β-Damascenone	tr				0.1				MS, RI
1379	Modheph-2-ene	tr	tr							MS, RI
1380	β-Bourbonene	0.1	1.0	0.1						MS, RI
1389	Dodecan-2-one	tr				0.1	tr			MS, RI, CoI
1395	β-Elemene	0.3		1.3	1.6					MS, RI
1397	Cyperene	tr	0.3							MS, RI
1400	Tetradecane	tr	0.2							MS, RI, CoI
1403	(*Z*)-Caryophyllene	tr		0.1	tr					MS, RI
1407	α-Gurjunene	tr	tr							MS, RI
1411	β-Ylangene	tr			tr					MS, RI
1415	(*E*)-Caryophyllene	5.2	6.9	34.9						MS, RI, CoI
1426	Isogermacrene D	0.1	0.5							MS, RI
1427	γ-Elemene	tr			0.3					MS, RI
1430	β-Copaene	tr	tr	0.7						MS, RI
1439	Neryl acetone	tr				0.1	0.1			MS, RI, CoI
1447	(*E*)-β-Farnesene	tr			tr					MS, RI
1451	α-Humulene	0.5		1.1	1.3					MS, RI, CoI
1474	γ-Muurolene	0.2	0.5							MS, RI
1479	(*E*)-β-Ionone	tr				0.1	0.5	0.3		MS, RI
1482	Germacrene D	17.2		42.6						MS, RI, CoI
1491	Tridecan-2-one	1.4			4.4	12.2	4.6	0.1		MS, RI, CoI
1494	Pentadec-1-ene	4.8	45.9							MS, RI
1495	Bicyclogermacrene	tr	tr	tr	1.1					MS, RI
1496	α-Muurolene	tr	0.8							MS, RI
1498	(*E*,*E*)-α-Farnesene	0.6			1.3					MS, RI
1500	Pentadecane	tr	0.9							MS, RI, CoI
1511	γ-Cadinene	0.2	0.2	1.1						MS, RI
1519	δ-Cadinene	0.5	0.4	1.9						MS, RI
1527	Isokessane	0.4			15.3	1.8				MS, RI
1530	*trans*-Cadina-1,4-diene	tr		tr						MS, RI
1535	Kessane	1.5			48.0	12.8	0.1			MS, RI
1540	α-Calacorene	0.1			0.2	tr	tr			MS, RI
1546	Elemol	0.1							0.4	MS, RI
1553	Isocaryophyllene oxide	0.2				1.6	1.2	0.1		MS, RI
1558	Germacrene B	0.9		1.4						MS, RI
1558	(*E*)-Nerolidol	tr						1.0		MS, RI
1562	β-Calacorene	tr				tr	tr			MS, RI
1566	1,5-Epoxysalvial-4(14)-ene	0.2			0.6	1.2	tr			MS, RI
1578	Spathulenol	0.3						0.4		MS, RI
1578	Germacrene D-4-ol	tr					0.1	0.4		MS, RI
1587	Caryophyllene oxide	1.2				14.5	15.0	2.3		MS, RI, CoI
1589	Viridiflorol	tr						1.0	tr	MS, RI
1593	Tetradecan-2-one	tr				1.0				MS, RI, CoI
1594	Hexadec-1-ene	tr	0.3							MS, RI
1596	Salvial-4(14)-en-1-one	0.4			2.1	2.1	0.8	0.3		MS, RI
1600	Humulene epoxide I	tr				0.4	0.3			MS, RI
1600	Hexadecane	tr	0.2							MS, RI, CoI
1606	Tetradecanal	tr				0.3				MS, RI
1610	β-Oplopenone	0.8			5.2	9.4	3.2	0.4		MS, RI
1611	Humulene epoxide II	tr					tr			MS, RI
1617	10-*epi*-γ-Eudesmol	0.1						0.6		MS, RI
1624	Junenol	0.5				2.1	1.6			MS, RI
1630	1-*epi*-Cubenol	tr						0.4		MS, RI
1632	Guaia-6,10(14)-dien-4β-ol	1.6							1.9	MS, RI
1643	*epi*-α-Murrolol	0.1						1.0		MS, RI
1645	Cubenol	tr			tr	0.9				MS, RI
1646	α-Muurolol	0.3							0.6	MS, RI
1658	α-Cadinol	2.2							3.3	MS, RI
1672	Tetradecan-1-ol	0.8							1.3	MS, RI, CoI
1689	Germacra-4(15),5,10(14)-trien-1α-ol	0.6							0.7	MS, RI
1694	Heptadec-1-ene	0.4	6.3							MS, RI
1695	Pentadecan-2-one	tr			tr	1.0	0.4			MS, RI, CoI
1700	Heptadecane	tr	tr							MS, RI, CoI
1710	Pentadecanal	tr			2.9	1.0	0.5	0.3		MS, RI, CoI
1744	Mint sulfide	0.1			2.1					MS, RI
1762	2-Oxoundec-7-en-1-yl isobutyrate *	tr					0.4			**NEW**
1769	2-Oxoundecyl isobutyrate	tr					0.3			**NEW**
1779	14-Oxy-α-muurolene	tr			0.6	1.3	0.5	0.4		MS, RI
1800	Octadecane	tr	tr							MS, RI, CoI
1815	(7*Z*,9*E*)-2-Oxoundeca-7,9-dien-1-yl isobutyrate ^#^	0.8					0.9			**NEW**
1842	Spilanthol isomer ^e^	tr							1.7	MS
1856	2-Oxoundec-7-en-1-yl isovalerate *	0.2					1.3	0.9		**NEW**
1863	2-Oxoundecyl isovalerate	tr					tr			**NEW**
1869	2-Oxododecyl isobutyrate	tr					tr			**NEW**
1876	Hexadecan-1-ol	0.3							0.6	MS, RI, CoI
1891	(2*E*,6*Z*,8*E*)-*N*-Isobutyldeca-2,6,8-trienamide (syn. spilanthol)	28.9							56.0	MS, RI
1894	Nonadec-1-ene	tr	0.1							MS, RI
1898	Heptadecan-2-one	tr				tr	tr			MS, RI, CoI
1900	Nonadecane	tr	0.2							MS, RI, CoI
1900	2-Oxoundec-7-en-1-yl angelate *	0.4				0.7	0.5			**NEW**
1908	2-Oxoundecyl angelate	tr				tr	0.1			**NEW**
1909	Spilanthol isomer ^f^	1.2							2.9	MS
1910	(7*Z*,9*E*)-2-Oxoundeca-7,9-dien-1-yl isovalerate ^#^	2.4					6.6	18.0		**NEW**
1912	Spilanthol isomer ^g^	1.1							1.8	MS
1916	(5*E*,9*E*)-Farnesyl acetone	tr				0.3	tr			MS, RI
1917	Spilanthol isomer ^h^	1.4							2.1	MS
1923	(7*E*,9*E*)-2-Oxoundeca-7,9-dien-1-yl isovalerate ^#^	tr					0.8	2.0		**NEW**
1925	(7*E*,9*Z*)-2-Oxoundeca-7,9-dien-1-yl isovalerate ^#^	tr					0.4	0.9		**NEW**
1929	(7*Z*,9*Z*)-2-Oxoundeca-7,9-dien-1-yl isovalerate ^#^	tr					0.4	1.3		**NEW**
1940	2-Oxoundec-7-en-1-yl senecioate *	0.4				0.6	3.8	3.2		**NEW**
1948	2-Oxoundecyl senecioate	0.2				0.7	1.9	1.3		**NEW**
1952	2-Oxotridec-7-en-1-yl isobutyrate *	tr					tr			**NEW**
1956	(7*Z*,9*E*)-2-Oxoundeca-7,9-dien-1-yl angelate ^#^	0.8				1.9	8.1	3.7		**NEW**
1964	2-Oxododecyl isovalerate	tr				tr	tr	tr		**NEW**
1966	*N*-(2-Methylbutyl)deca-2,6,8-trienamide isomer ^i^	tr							0.1	MS
1969	(7*E*,9*E*)-2-Oxoundeca-7,9-dien-1-yl angelate ^#^	tr				0.6	1.5	0.9		**NEW**
1969	2-Oxotridecyl isobutyrate	tr				0.3	1.2	tr		**NEW**
1971	(7*E*,9*Z*)-2-Oxoundeca-7,9-dien-1-yl angelate ^#^	tr					tr	tr		**NEW**
1975	(7*Z*,9*Z*)-2-Oxoundeca-7,9-dien-1-yl angelate ^#^	tr				0.3	0.8	0.4		**NEW**
1979	Hexadecanoic acid	tr							6.1	MS, RI, CoI
1995	(7*Z*,9*E*)-2-Oxoundeca-7,9-dien-1-yl senecioate (syn. acmellonate)	4.7				0.1	5.8	30.8		MS, RI
2000	Eicosane	tr	0.7							MS, RI, CoI
2009	(7*E*,9*E*)-2-Oxoundeca-7,9-dien-1-yl senecioate ^#^	0.3					1.5	4.1		**NEW**
2009	2-Oxododecyl angelate	0.8				0.1	tr			**NEW**
2011	(7*E*,9*Z*)-2-Oxoundeca-7,9-dien-1-yl senecioate ^#^	0.2					tr	1.8		**NEW**
2014	*N*-(2-Methylbutyl)deca-2,6,8-trienamide isomer ^j^	tr							3.5	MS
2015	(7Z,9Z)-2-Oxoundeca-7,9-dien-1-yl senecioate ^#^	0.3					0.8	2.5		**NEW**
2023	*N*-(2-Methylbutyl)deca-2,6,8-trienamide isomer ^k^	tr							0.2	MS
2026	*N*-(2-Methylbutyl)deca-2,6,8-trienamide isomer ^l^	tr							0.2	MS
2030	*N*-(2-Methylbutyl)deca-2,6,8-trienamide isomer ^m^	tr							0.2	MS
2035	*N*-(2-Methylbutyl)deca-2,6,8-trienamide isomer ^n^	tr							0.2	MS
2031	2-Oxotridec-6-en-1-yl isovalerate *	tr				0.6	1.5	tr		**NEW**
2047	2-Oxotridec-7-en-1-yl isovalerate *	tr					tr	tr		**NEW**
2049	2-Oxododecyl senecioate	0.3				0.7	1.8	0.4		**NEW**
2065	2-Oxotridecyl isovalerate	0.1				2.1	2.2	0.3		**NEW**
2078	2-Oxotridec-6-en-1-yl angelate *	0.1				0.9	0.3	tr		**NEW**
2092	2-Oxotridec-7-en-1-yl angelate *	tr				0.3	0.1			**NEW**
2100	Heneicosane	tr	1.1							MS, RI, CoI
2110	2-Oxotridecyl angelate	tr				0.9	tr			**NEW**
2113	(*E*)-Phytol	2.3						1.3	2.6	MS, RI
2119	2-Oxotridec-6-en-1-yl senecioate *	0.8				2.8	6.3	2.8	tr	**NEW**
2133	2-Oxotridec-7-en-1-yl senecioate *	0.1				0.8	1.2	0.6		**NEW**
2146	(9*Z*,12*Z*)-Octadeca-9,12-dienoic acid	tr							1.1	MS, RI, CoI
2150	2-Oxotridecyl senecioate	0.8				10.1	13.2	2.8		**NEW**
2151	(9*Z*,12*Z*,15*Z*)-Octadeca-9,12,15-trienoic acid	tr							1.4	MS, RI
2200	Docosane	tr	1.5	tr	tr	tr				MS, RI, CoI
2300	Tricosane	0.1	2.8	tr	tr	tr				MS, RI, CoI
2400	Tetracosane	tr	2.1	0.3	0.3					MS, RI, CoI
2430	Docosanal	tr			tr					MS, RI
2462	2-Methyltetracosane	tr	0.1							MS, RI
2474	3-Methyltetracosane	tr	0.1							MS, RI
2500	Pentacosane	0.5	6.4	tr	tr					MS, RI, CoI
2600	Hexacosane	tr	1.2	0.4	0.5	0.1				MS, RI, CoI
2663	2-Methylhexacosane	tr	0.2							MS, RI
2674	3-Methylhexacosane	tr	0.1							MS, RI
2700	Heptacosane	0.1	2.8	0.5	0.5	0.1				MS, RI, CoI
2763	2-Methylheptacosane	tr	0.1							MS, RI
2774	3-Methylheptacosane	tr	0.1							MS, RI
2800	Octacosane	tr	0.8	0.2	0.3	0.1				MS, RI, CoI
2824	Supraene	tr			1.5					MS, RI, CoI
2864	2-Methyloctacosane	tr	0.1							MS, RI
2900	Nonacosane	0.1	3.3	0.5	0.5	tr				MS, RI, CoI
2964	2-Methylnonacosane	tr	tr							MS, RI
2974	3-Methylnonacosane	tr	0.1							MS, RI
3000	Triacontane	tr	0.4	0.1	0.2	tr				MS, RI, CoI
3100	Hentriacontane	0.1	1.5	tr	0.3	tr				MS, RI, CoI
3200	Dotriacontane	tr	tr							MS, RI, CoI
3300	Tritriacontane	tr	tr							MS, RI, CoI
	Total identified (%)	97.0	95.2	90.4	91.2	89.5	92.7	90.9	89.2	

^a^ RI = retention indices determined relative to a homologous series of n-alkanes (C_7_–C_33_) on a DB-5MS column. ^b^ *syn*. = synonym. ^c^ The essential oil (sample EO) and dry-flash chromatographic fractions (samples F1–F7) of *A. oleracea* leaves and inflorescences collected in April 2019 from the district of Fazendinha, Macapá City, Amapá State, Brazil; tr = trace amounts (<0.05%). ^d^ ID = identification method; MS = constituent identified by mass-spectra comparison with those listed in the Wiley 11, NIST17, MassFinder 2.3, and a homemade mass spectral library; RI = constituent identified by retention index matching with literature data; CoI = constituent identity confirmed by GC co-injection of an authentic sample; NEW = new natural products and entirely new compounds. ^e^ MS (EI), *m*/*z*(%) 152(9), 151(35), 141(100), 126(34), 123(10), 115(6), 110(5), 109(8), 107(7), 99(7), 98(23), 95(11), 93(6), 91(8), 85(16), 84(10), 83(17), 82(7), 81(47), 79(14), 77(10), 72(6), 69(26), 68(34), 67(34), 66(4), 65(5), 57(13), 56(10), 55(65), 53(12), 43(25), 41(57). ^f^ MS (EI), *m*/*z*(%) 221(4), 141(34), 126(14), 98(10), 91(5), 82(7), 81(100), 79(24), 68(13), 53(13), 41(23). ^g^ MS (EI), *m*/*z*(%) 221(4), 142(6), 141(58), 126(25), 98(19), 91(8), 85(9), 81(100), 79(34), 68(19), 53(18), 41(34). ^h^ MS (EI), *m*/*z*(%) 221(4), 193(12), 175(11), 159(5), 149(9), 141(46), 133(8), 123(27), 115(7), 105(17), 91(27), 83(9), 81(100), 79(47), 69(22), 55(28), 41(52). ^i^ MS (EI), *m*/*z*(%) 236(5), 156(6), 155(48), 152(8), 151(26), 141(24), 126(18), 121(12), 115(12), 112(9), 81(100), 80(10), 79(36), 77(19), 69(58), 68(38), 67(45), 66(6), 65(10), 57(31), 55(12), 53(21), 43(68), 41(33). ^j^ MS (EI), *m*/*z*(%) 235(8), 156(8), 155(76), 140(15), 126(20), 121(4), 114(6), 112(5), 99(12), 98(19), 93(9), 91(6), 86(18), 85(8), 84(6), 82(7), 81(100), 80(6), 79(37), 77(10), 71(5), 69(15), 68(23), 67(5), 66(4), 65(5), 57(6), 55(12), 53(20), 43(11), 41(33). ^k^ MS (EI), *m*/*z*(%) 235(6), 179(5), 156(10), 155(100), 140(13), 114(12), 112(51), 99(44), 98(20), 93(5), 91(5), 84(16), 82(5), 81(79), 80(7), 79(48), 77(9), 69(36), 68(22), 67(5), 66(5), 65(5), 55(21), 53(23), 43(16), 41(39). ^l^ MS (EI), *m*/*z*(%) 235(5), 155(36), 140(5), 126(10), 121(4), 99(7), 98(6), 86(6), 82(7), 81(100), 79(30), 69(5), 68(15), 55(8), 53(12), 41(17). ^m^ MS (EI), *m*/*z*(%) 235(9), 156(11), 155(82), 143(5), 141(7), 127(5), 126(11), 107(8), 99(10), 92(6), 91(5), 87(5), 86(13), 85(6), 82(9), 81(100), 79(28), 68(16), 65(4), 53(13), 43(18), 41(12). ^n^ MS (EI), *m*/*z*(%) 155(58), 149(4), 126(13), 123(5), 119(5), 114(5), 112(5), 109(5), 107(9), 99(14), 98(25), 94(7), 93(26), 92(5), 91(10), 85(6), 81(100), 80(10), 79(39), 77(10), 70(9), 69(44), 68(29), 67(18), 66(4), 65(5), 56(6), 55(17), 53(17), 43(16), 41(29). * Exact configuration of the double-bond was not determined. ^#^ Exact position and configuration of the double-bonds were determined only tentatively.

## Data Availability

Data are contained within the article and supplementary materials.

## Supplementary Materials

The following supporting information can be downloaded at: https://www.mdpi.com/article/10.3390/plants13121690/s1.
