# Supplementary material for: Amazon Rainforest Hidden Volatiles—Part I: Unveiling New Compounds from Acmella oleracea (L.) R.K. Jansen Essential Oil
Source: Plants (Basel). 2024 Jun 19;13(12):1690. doi: 10.3390/plants13121690 (PMC11207513; doi:10.3390/plants13121690)
Supplement: Supplementary file 1 [file plants-13-01690-s001.zip › plants-3011907-supplementary.pdf]

Supplementary material

Amazon rainforest hidden volatiles. Part I: Unveiling new compounds from *Acmella oleracea* (L.) R.K. Jansen essential oil

Niko S. Radulović [1,*], Marko Z. Mladenović [1], Clarissa Silva Lima [2], Elza Caroline Alves Müller [2], Elizabeth Vianna Moraes da Costa [2], Rozilene Valadares Martins [3], Fabio Boylan [4]

[1] Department of Chemistry, Faculty of Sciences and Mathematics, University of Niš, Višegradska 33, 18000 Niš, Serbia
[2] Department of Biological Sciences and Health of the Federal University of Amapá, Macapá, Highway Juscelino Kubitschek, Km 02, Macapá 68903-197, Brazil
[3] Postgraduate Program in Health Sciences, Federal University of Amapá, Highway Juscelino Kubitschek, Km 02, Macapá 68903-197, Brazil
[4] School of Pharmacy and Pharmaceutical Sciences, Panoz Institute, and Trinity Biomedical Sciences Institute, Trinity College Dublin, Dublin 2, D02 PN40 Dublin, Ireland
* Correspondence: nikoradulovic@yahoo.com (N.S.R.); fabio.boylan@tcd.ie (F.B.); Tel.: +381-18-533-015 (N.S.R.); +353-1-896-4154 (F.B.); Fax: +381-18-533-014 (N.S.R.)

Content:

Figure S1. From top to bottom: Part of the GC chromatogram, the mass spectrum of the acmellonate, and the mass spectrum of the 2-oxoundecyl senecioate
Figure S2. GC-MS chromatograms of the essential oil chromatographic fractions
Figure S3. Mass spectrum of 1-hydroxyundecan-2-one (**1**)
Figure S4. Mass spectrum of 2-oxoundecyl isobutyrate (**1a**)
Figure S5. Mass spectrum of 2-oxoundecyl 2-methylbutanoate (**1b**)
Figure S6. Mass spectrum of 2-oxoundecyl 3-methylbutanoate (**1c**)
Figure S7. Mass spectrum of 2-oxoundecyl angelate (**1d**)
Figure S8. Mass spectrum of 2-oxoundecyl tiglate (**1e**)
Figure S9. Mass spectrum of 2-oxoundecyl senecioate (**1f**)
Figure S10. Mass spectrum of 1-hydroxydodecan-2-one (**2**)
Figure S11. Mass spectrum of 2-oxododecyl isobutyrate (**2a**)
Figure S12. Mass spectrum of 2-oxododecyl 2-methylbutanoate (**2b**)
Figure S13. Mass spectrum of 2-oxododecyl 3-methylbutanoate (**2c**)
Figure S14. Mass spectrum of 2-oxododecyl angelate (**2d**)
Figure S15. Mass spectrum of 2-oxododecyl tiglate (**2e**)
Figure S16. Mass spectrum of 2-oxododecyl senecioate (**2f**)
Figure S17. Mass spectrum of 1-hydroxytridecan-2-one (**3**)
Figure S18. ^{1}H NMR spectrum of 1-hydroxytridecan-2-one (**3**)
Figure S19. ^{13}C NMR spectrum of 1-hydroxytridecan-2-one (**3**)
Figure S20. Mass spectrum of 2-oxotridecyl isobutyrate (**3a**)
Figure S21. ^{1}H NMR spectrum of 2-oxotridecyl isobutyrate (**3a**) recorded in $CDCl_3$
Figure S22. ^{13}C NMR spectrum of 2-oxotridecyl isobutyrate (**3a**) recorded in $CDCl_3$
Figure S23. Mass spectrum of 2-oxotridecyl 2-methylbutanoate (**3b**)

Figure S24. ^{1}H NMR spectrum of 2-oxotridecyl 2-methylbutanoate (**3b**) recorded in $CDCl_3$
Figure S25. ^{13}C NMR spectrum of 2-oxotridecyl 2-methylbutanoate (**3b**) recorded in $CDCl_3$
Figure S26. Mass spectrum of 2-oxotridecyl 3-methylbutanoate (**3c**)
Figure S27. ^{1}H NMR spectrum of 2-oxotridecyl 3-methylbutanoate (**3c**) recorded in $CDCl_3$
Figure S28. ^{13}C NMR spectrum of 2-oxotridecyl 3-methylbutanoate (**3c**) recorded in $CDCl_3$
Figure S29. Mass spectrum of 2-oxotridecyl angelate (**3d**)
Figure S30. ^{1}H NMR spectrum of 2-oxotridecyl angelate (**3d**) recorded in $CDCl_3$
Figure S31. ^{13}C NMR spectrum of 2-oxotridecyl angelate (**3d**) recorded in $CDCl_3$
Figure S32. Mass spectrum of 2-oxotridecyl tiglate (**3e**)
Figure S33. ^{1}H NMR spectrum of 2-oxotridecyl tiglate (**3e**) recorded in $CDCl_3$
Figure S34. ^{13}C NMR spectrum of 2-oxotridecyl tiglate (**3e**) recorded in $CDCl_3$
Figure S35. Mass spectrum of 2-oxotridecyl senecioate (**3f**)
Figure S36. ^{1}H NMR spectrum of 2-oxotridecyl senecioate (**3f**) recorded in $CDCl_3$
Figure S37. ^{13}C NMR spectrum of 2-oxotridecyl senecioate (**3f**) recorded in $CDCl_3$
Figure S38. ^{1}H – ^{1}H COSY spectrum of 2-oxotridecyl senecioate (**3f**)
Figure S39. DEPT 90 spectrum of 2-oxotridecyl senecioate (**3f**)
Figure S40. DEPT 135 spectrum of 2-oxotridecyl senecioate (**3f**)
Figure S41. Proton-coupled ^{13}C NMR spectrum of 2-oxotridecyl senecioate (**3f**)
Figure S42. grHSQC spectrum of 2-oxotridecyl senecioate (**3f**)
Figure S43. grHMBC spectrum of 2-oxotridecyl senecioate (**3f**)
Figure S44. ^{1}H NMR spectrum of the essential oil fraction F5
Figure S45. GC chromatogram before (A) and after (B) derivatization of the essential oil fraction F5 with dimethyl disulfide
Figure S46. Part of the patrial ion current chromatogram (ca. 24 – 32 min) (PIC, ions at *m*/*z* 71 (marked yellow), *m*/*z* 83 (red), and *m*/*z* 85 (blue) of the chromatographic fraction F5 of *A. oleracea* essential oil
Figure S47. Part of the chromatogram (A) and mass spectrum of the detected 2-oxoundec-7-en-1-yl senecioate (B)
Figure S48. Part of the GC chromatogram after derivatization of the essential oil fraction F5 with dimethyl disulfide and mass spectrum of the dimethyl disulfide adduct of the 2-oxoundec-7-en-1-yl senecioate
Figure S49. Part of the GC chromatogram before (**A**) and after derivatization of the essential oil fraction F5 with dimethyl disulfide (**B**) and mass spectrum of the dimethyl disulfide adduct of the 2-oxotridec-6-en-1-yl senecioate (**C**) and 2-oxotridec-7-en-1-yl senecioate (**D**)
Figure S50. Part of the GC chromatogram of the essential oil fraction F7, mass spectrum of the spilanthol (**A**), and MS of the spilanthol diastereoisomers (**B**, **C**, and **D**)
Figure S51. ^{1}H NMR spectrum of the essential oil fraction F7
Figure S52. ^{13}C NMR spectrum of the essential oil fraction F7 (**A**), DEPT-90 (**B**), DEPT-135 (**C**), and ^{13}C proton coupled NMR spectrum (**D**)
Figure S53. Part of the patrial ion current chromatogram (ca. 27 – 29 min) (PIC, ions at *m*/*z* 81 (marked blue) and 155 (red) of the chromatographic fraction F7 of *A. oleracea* essential oil and mass spectrum of one of the detected isomeric *N*-(2-methylbutyl)decatrienamide

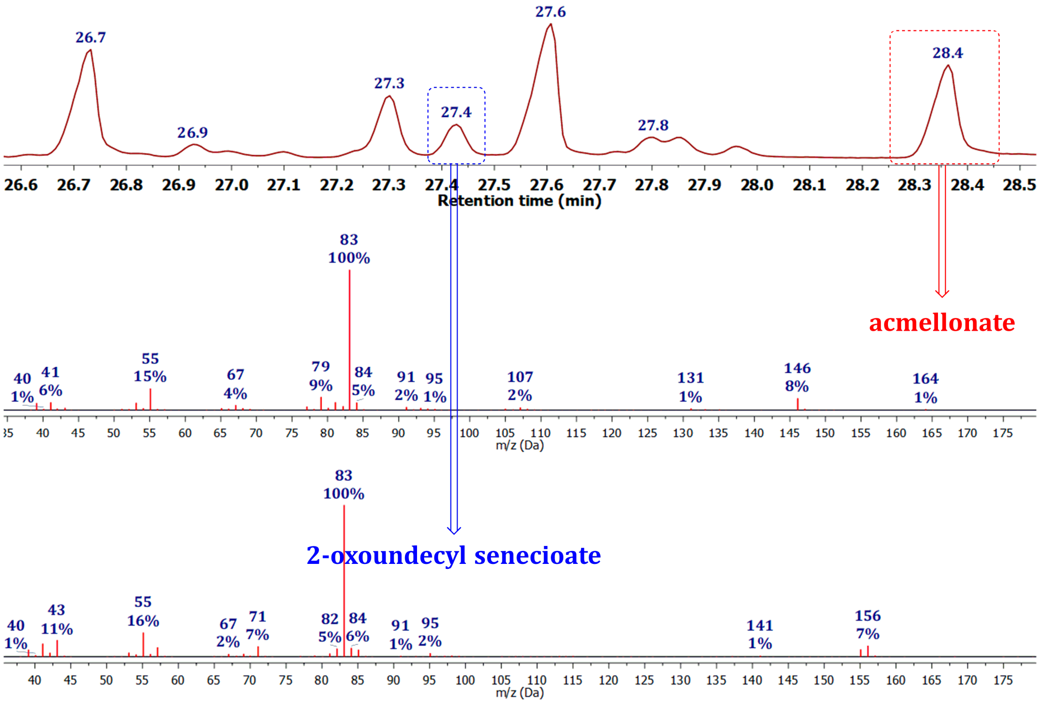

Figure S1. From top to bottom: Part of the GC chromatogram, the mass spectrum of the acmellonate, and the mass spectrum of the 2-oxoundecyl senecioate

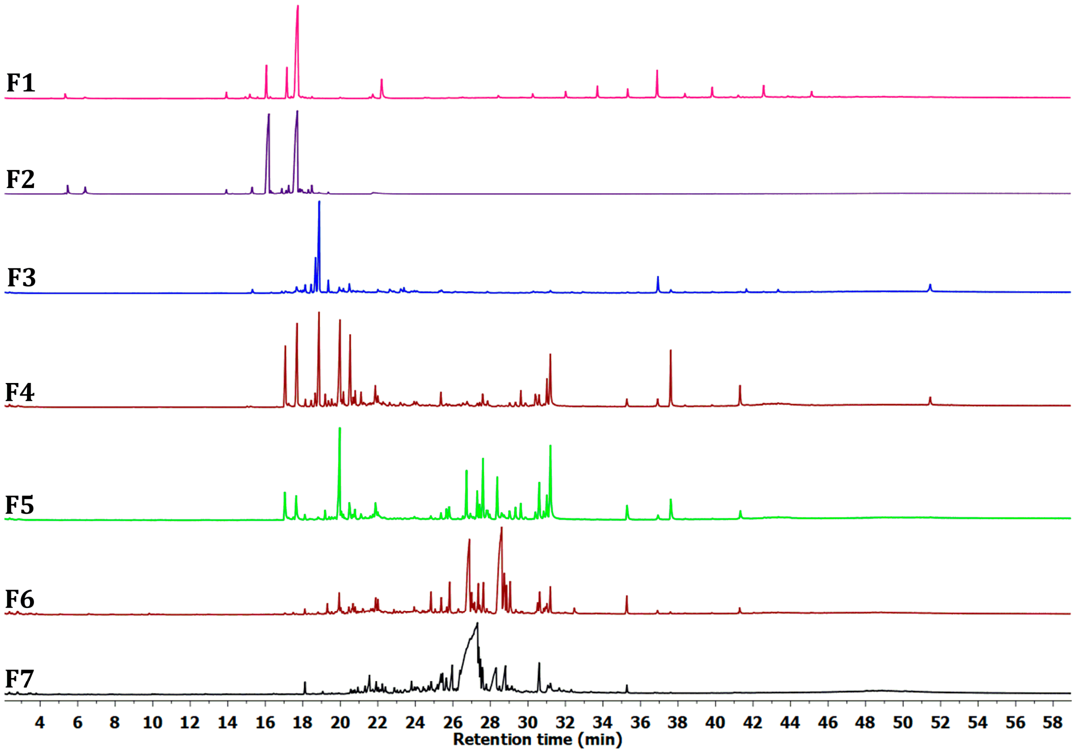

Figure S2. GC-MS chromatograms of the essential oil chromatographic fractions

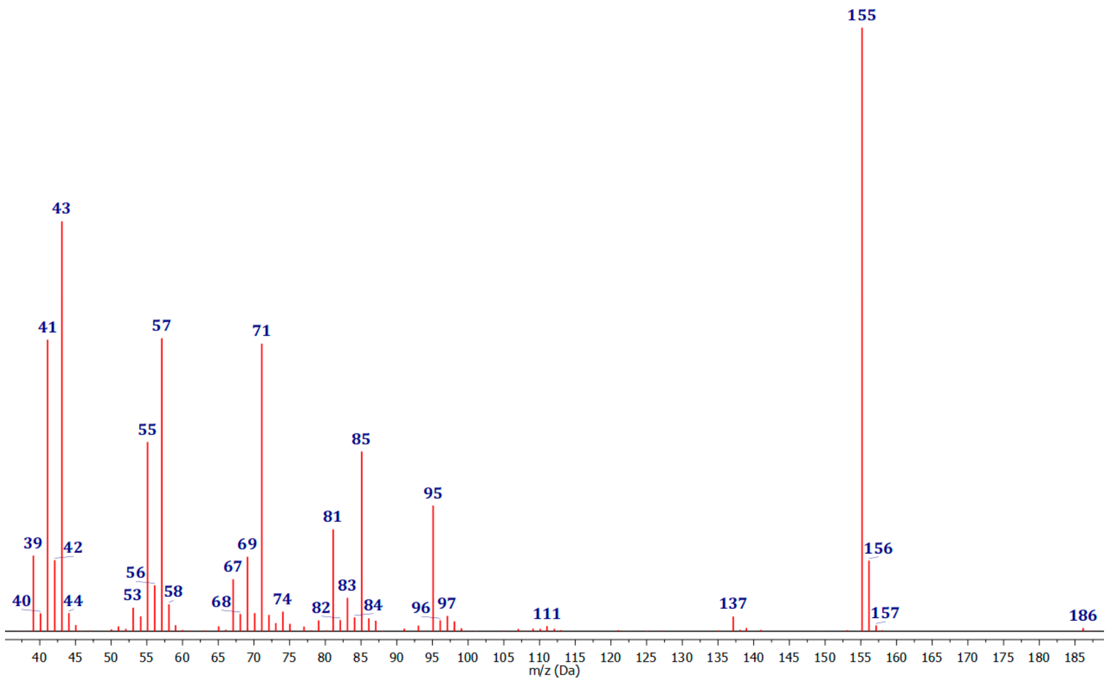

Figure S3. Mass spectrum of 1-hydroxyundecan-2-one (**1**)

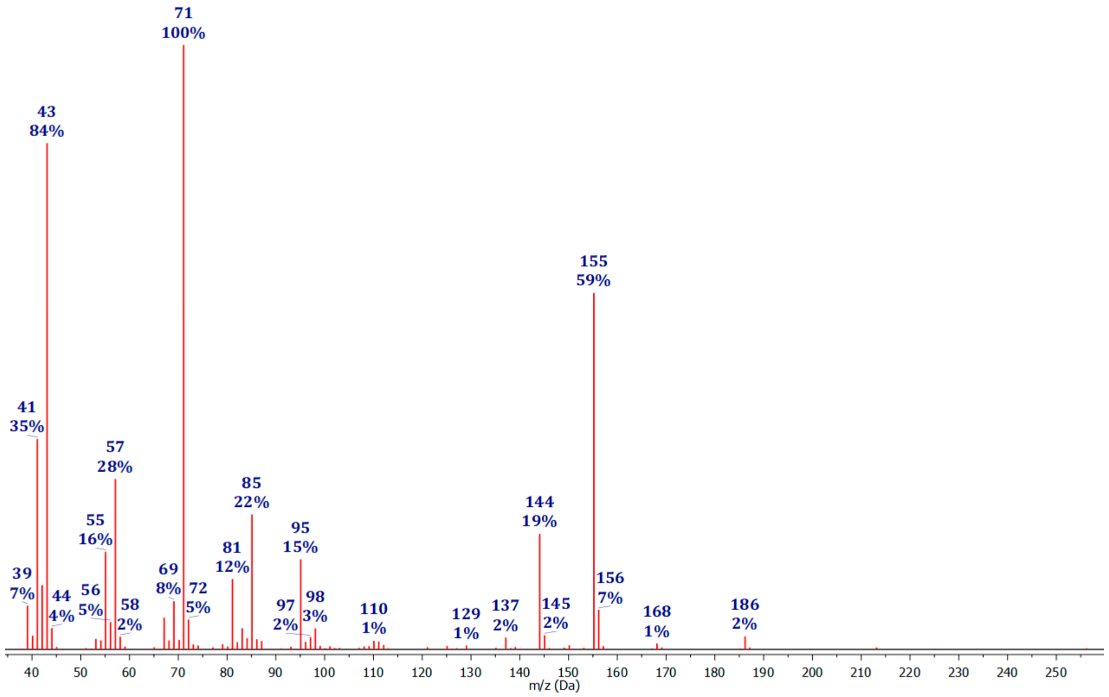

Figure S4. Mass spectrum of 2-oxoundecyl isobutyrate (**1a**)

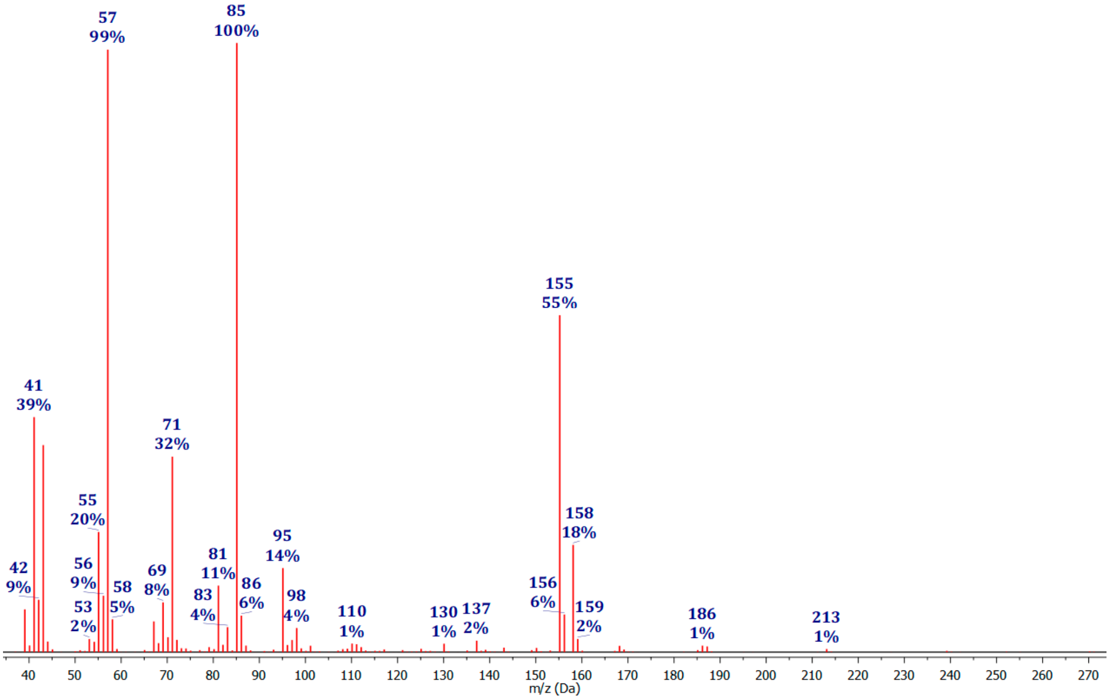

Figure S5. Mass spectrum of 2-oxoundecyl 2-methylbutanoate (**1b**)

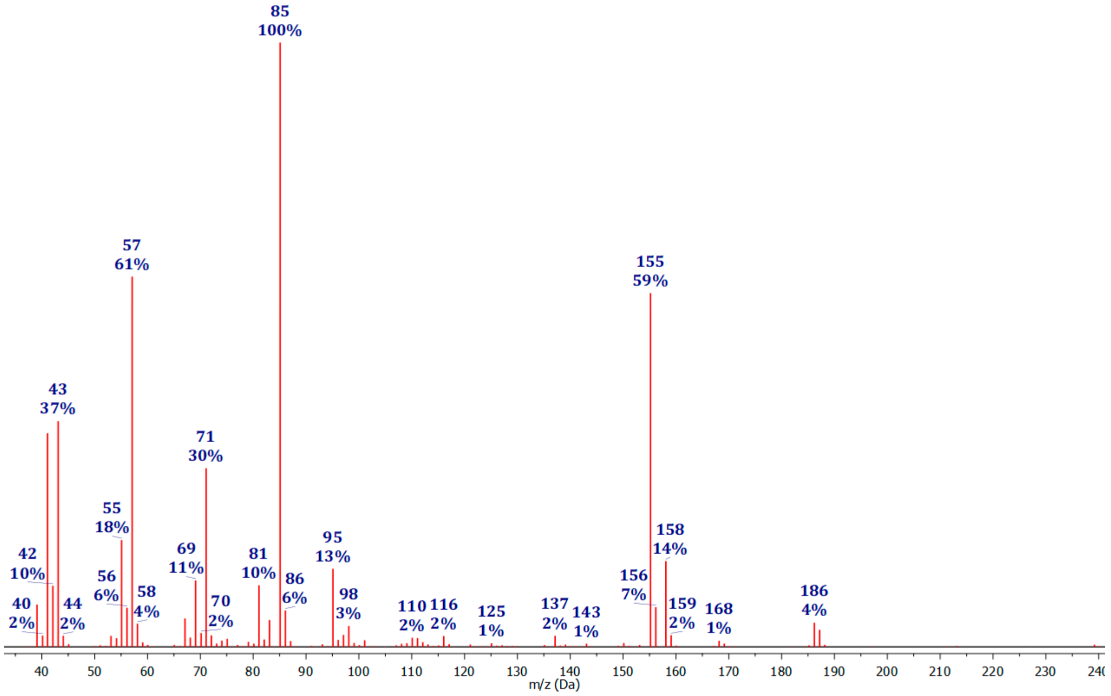

Figure S6. Mass spectrum of 2-oxoundecyl 3-methylbutanoate (**1c**)

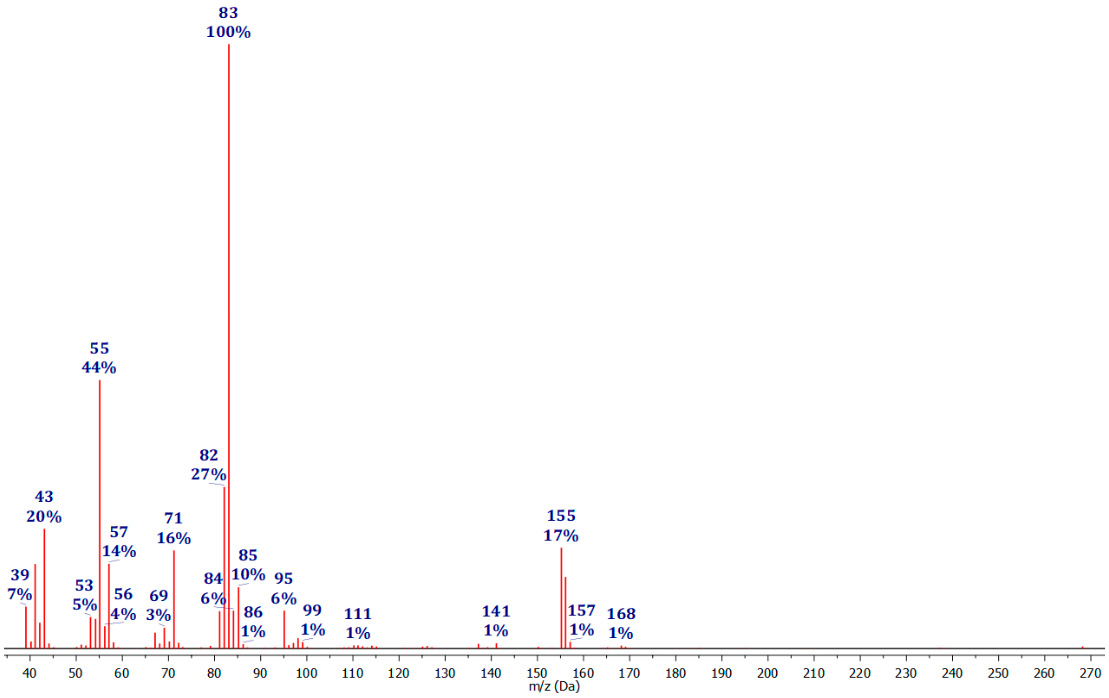

Figure S7. Mass spectrum of 2-oxoundecyl angelate (**1d**)

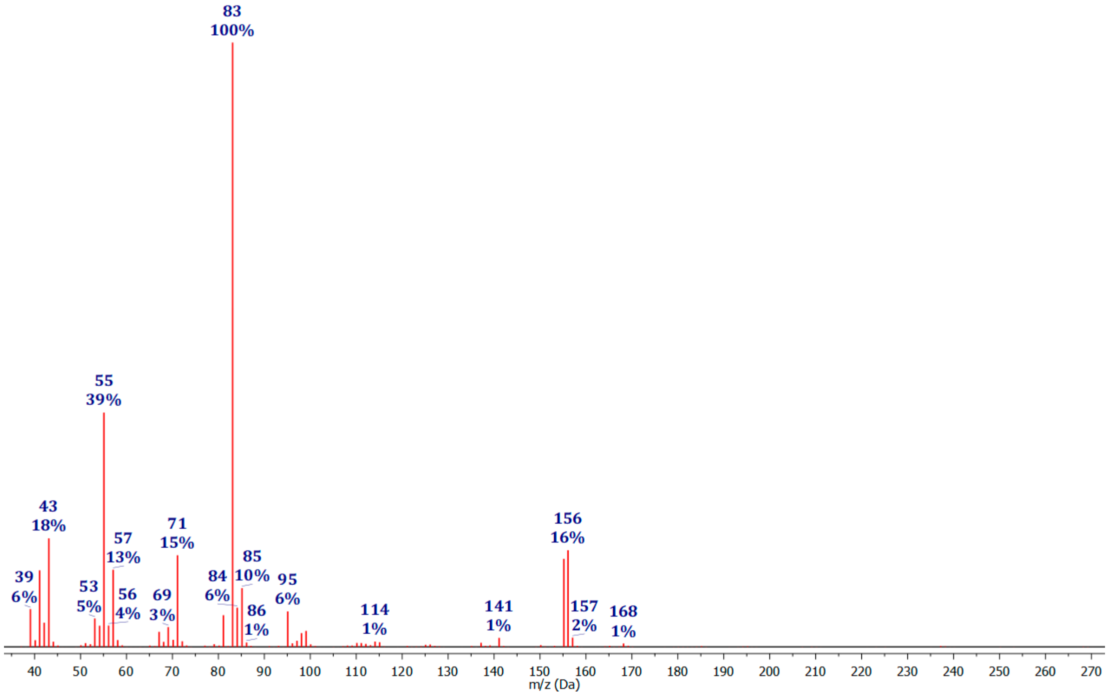

Figure S8. Mass spectrum of 2-oxoundecyl tiglate (**1e**)

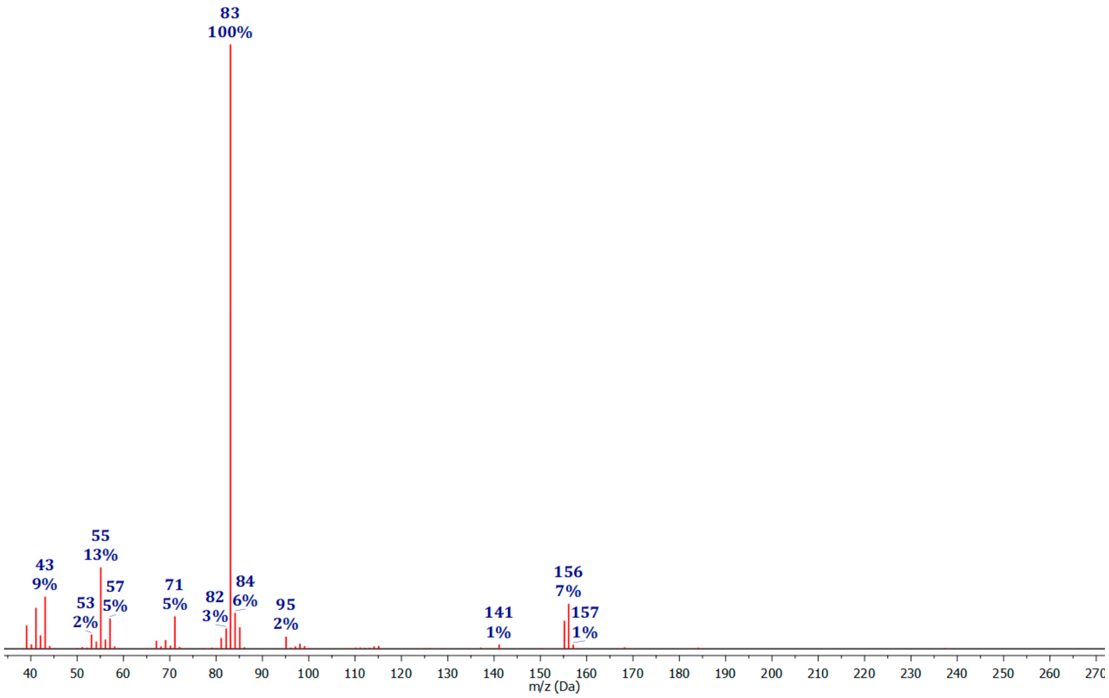

Figure S9. Mass spectrum of 2-oxoundecyl senecioate (**1f**)

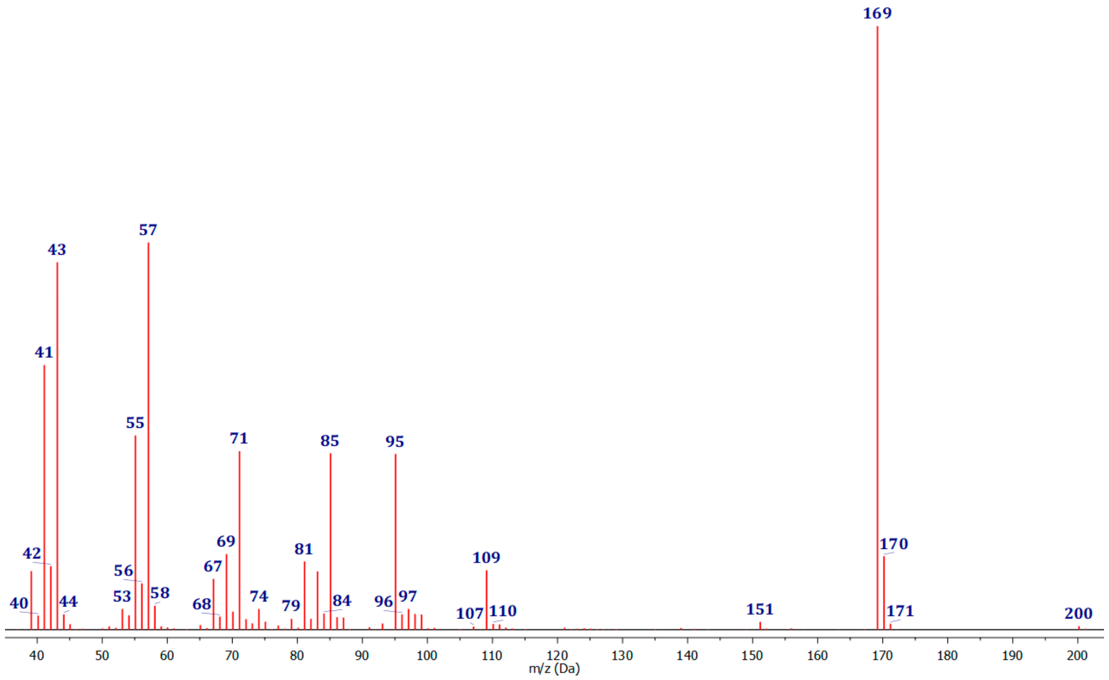

Figure S10. Mass spectrum of 1-hydroxydodecan-2-one (**2**)

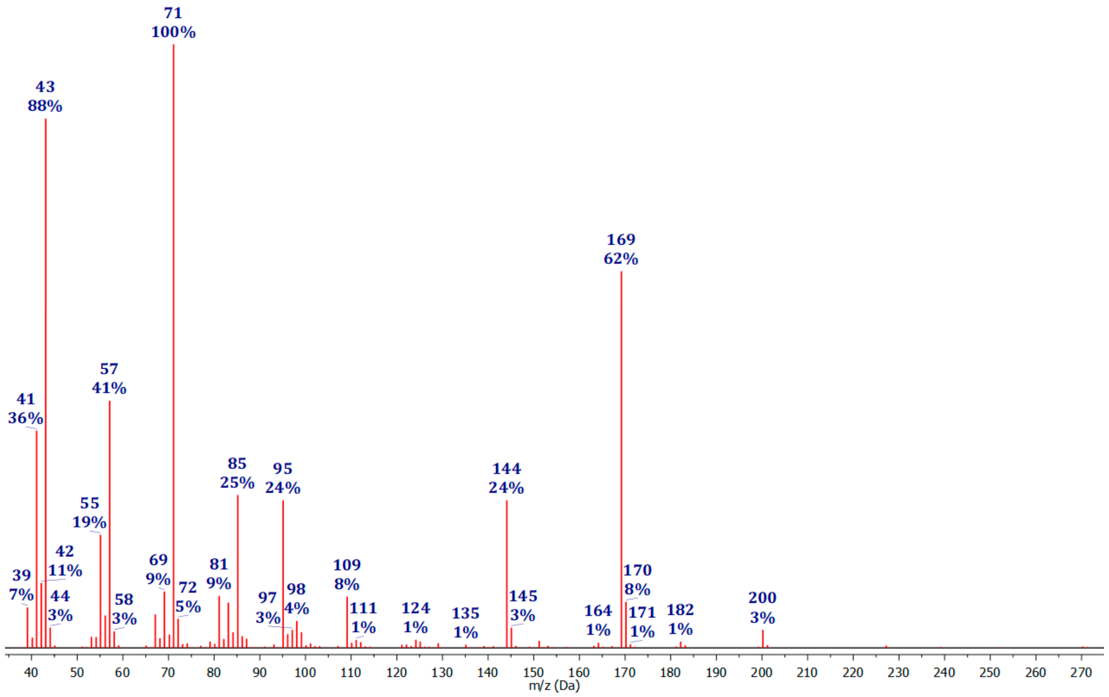

Figure S11. Mass spectrum of 2-oxododecyl isobutyrate (**2a**)

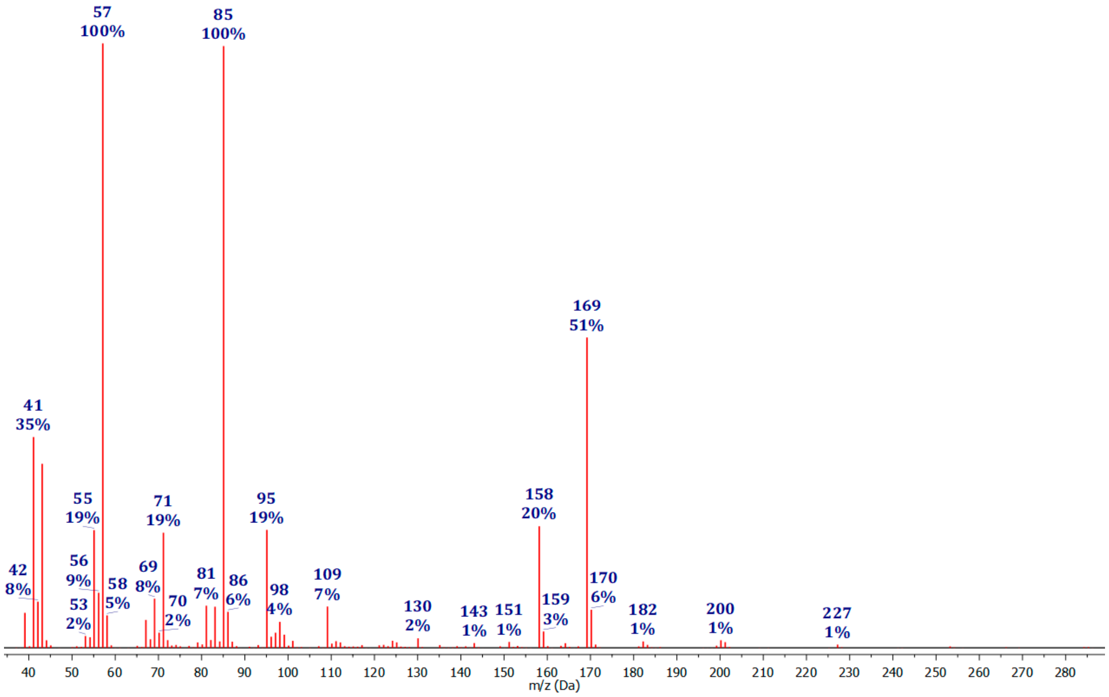

Figure S12. Mass spectrum of 2-oxododecyl 2-methylbutanoate (**2b**)

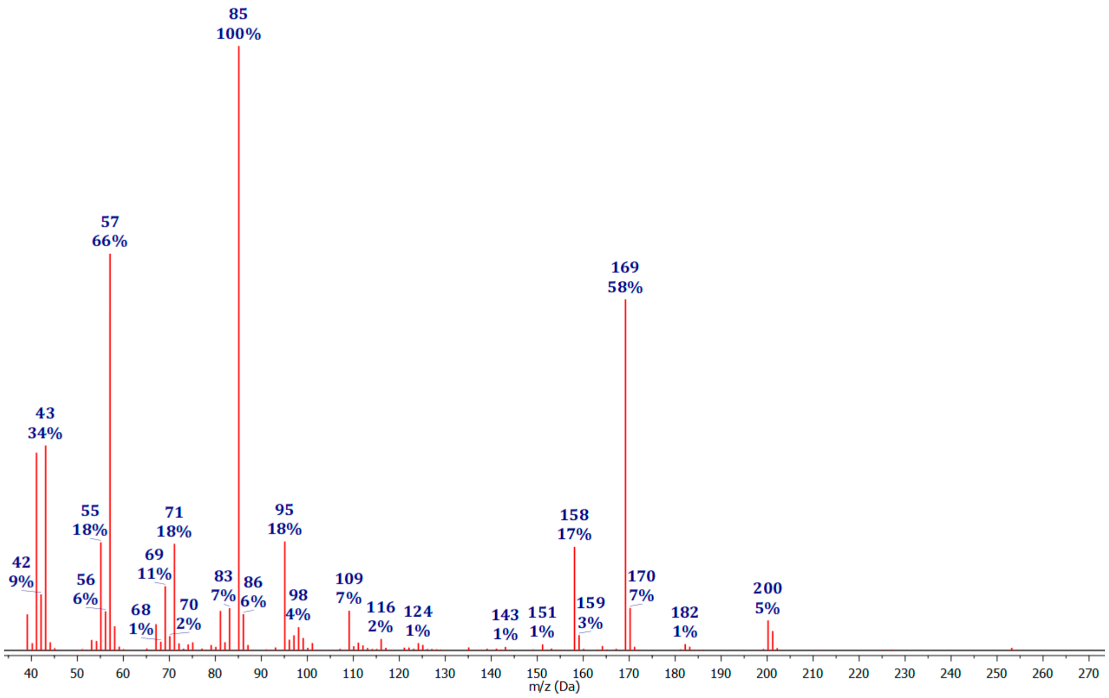

Figure S13. Mass spectrum of 2-oxododecyl 3-methylbutanoate (**2c**)

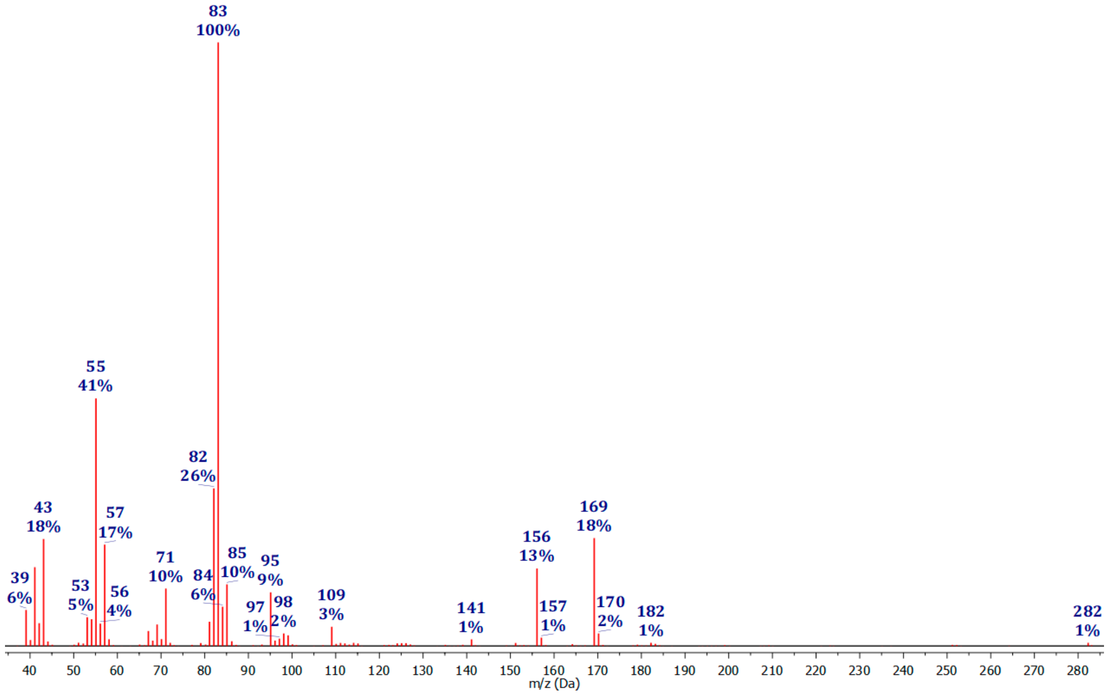

Figure S14. Mass spectrum of 2-oxododecyl angelate (**2d**)

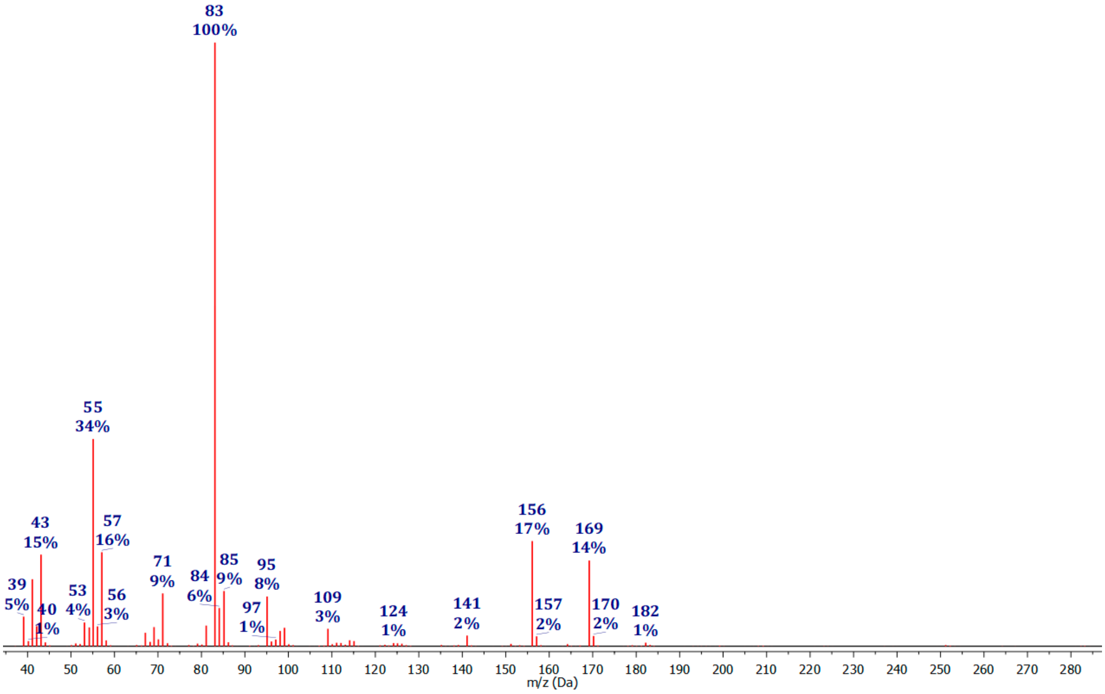

Figure S15. Mass spectrum of 2-oxododecyl tiglate (**2e**)

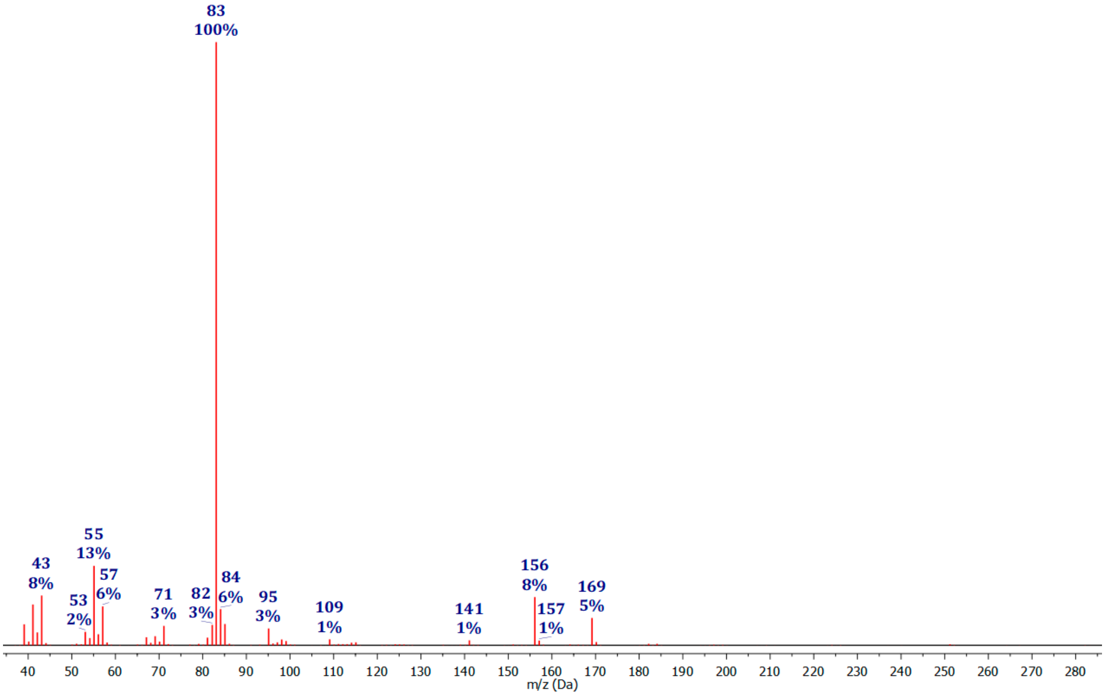

Figure S16. Mass spectrum of 2-oxododecyl senecioate (**2f**)

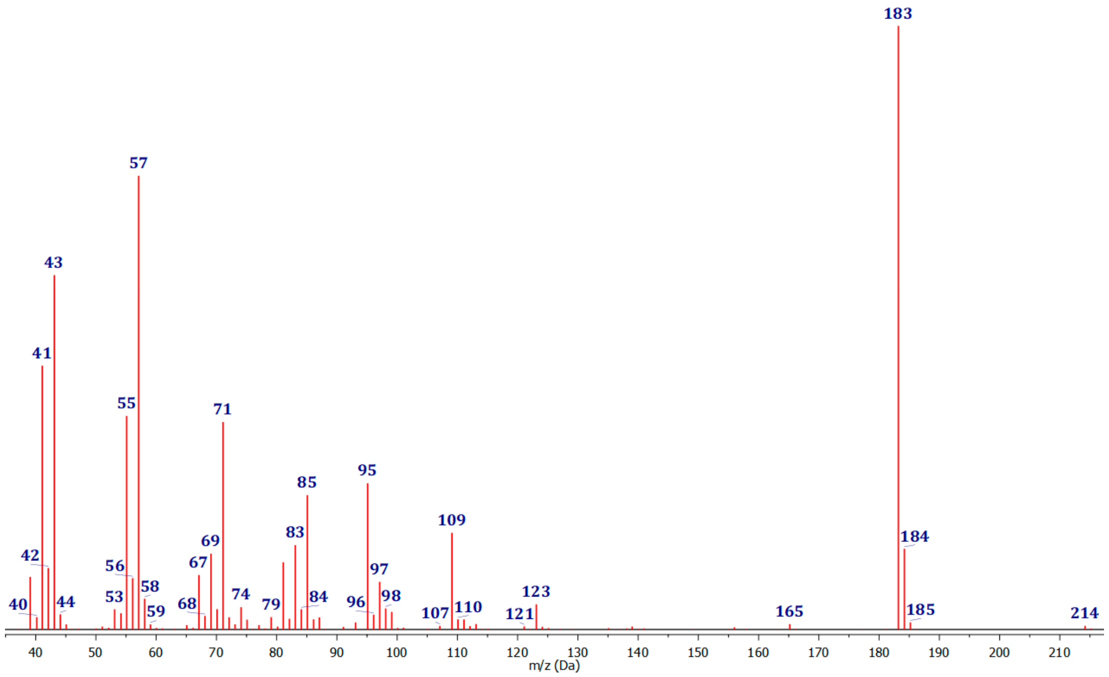

Figure S17. Mass spectrum of 1-hydroxytridecan-2-one (**3**)

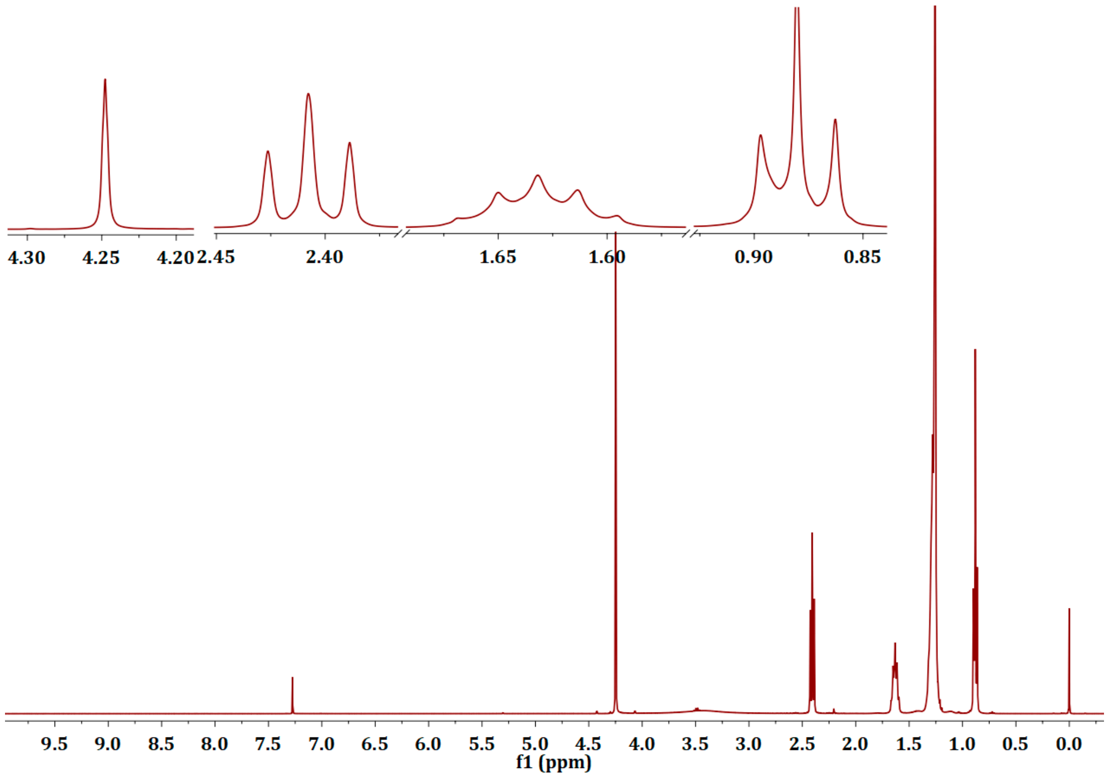

Figure S18. ^{1}H NMR spectrum of 1-hydroxytridecan-2-one (**3**)

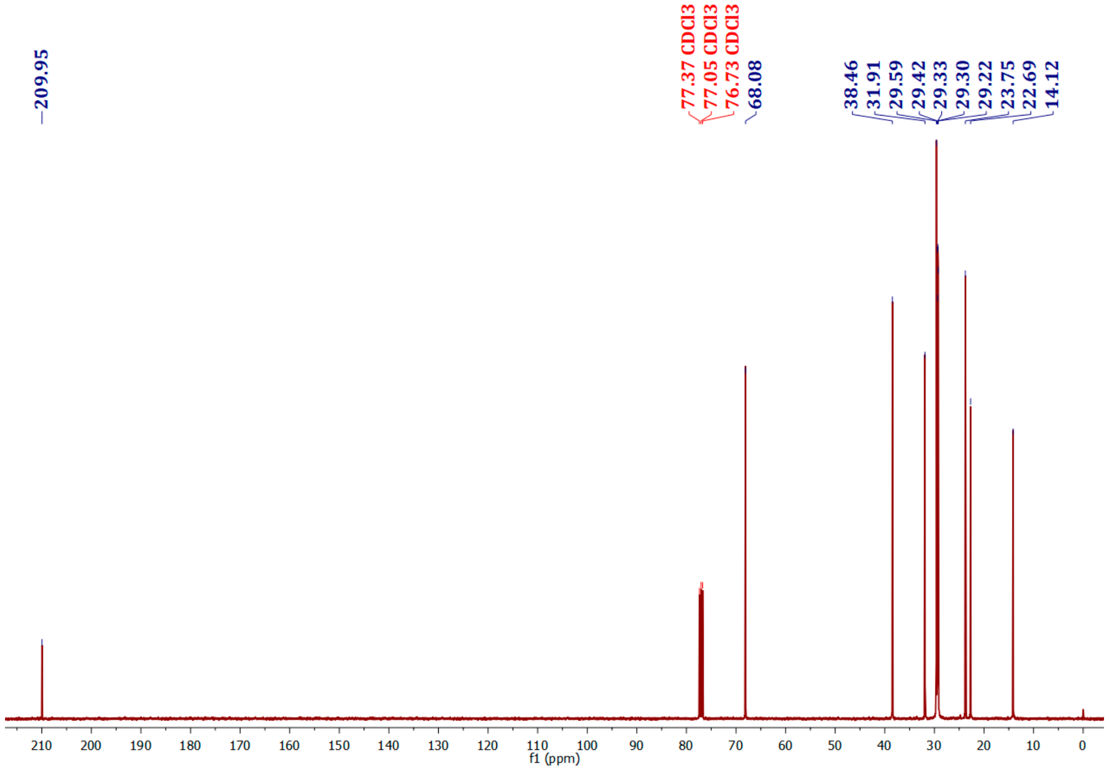

Figure S19. ^{13}C NMR spectrum of 1-hydroxytridecan-2-one (**3**)

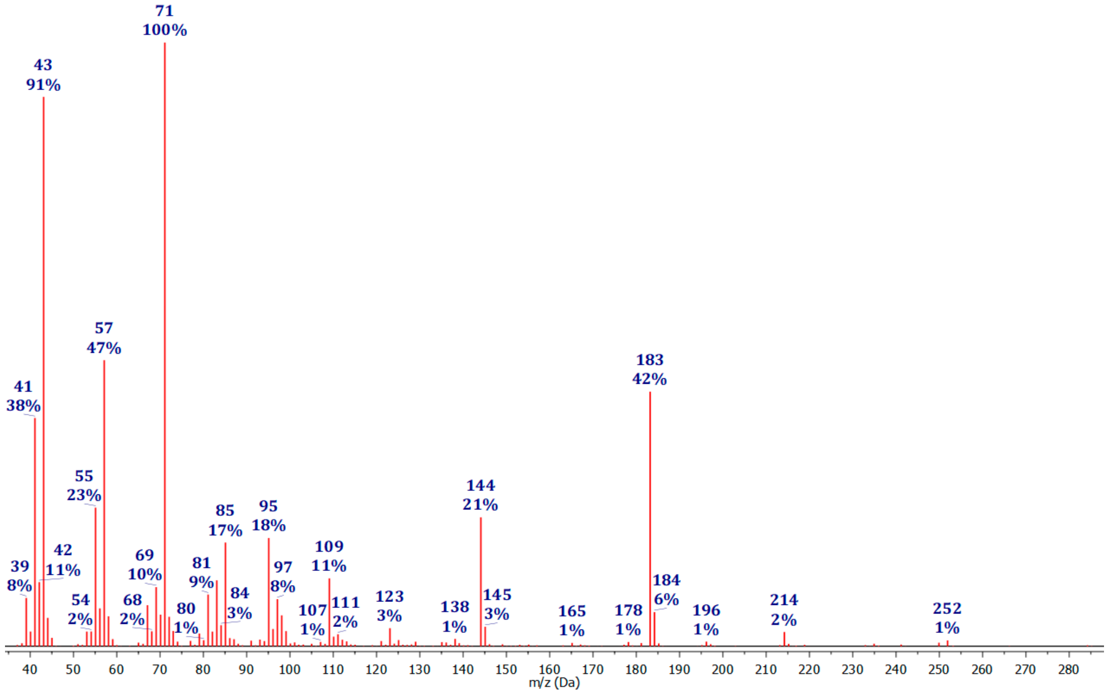

Figure S20. Mass spectrum of 2-oxotridecyl isobutyrate (**3a**)

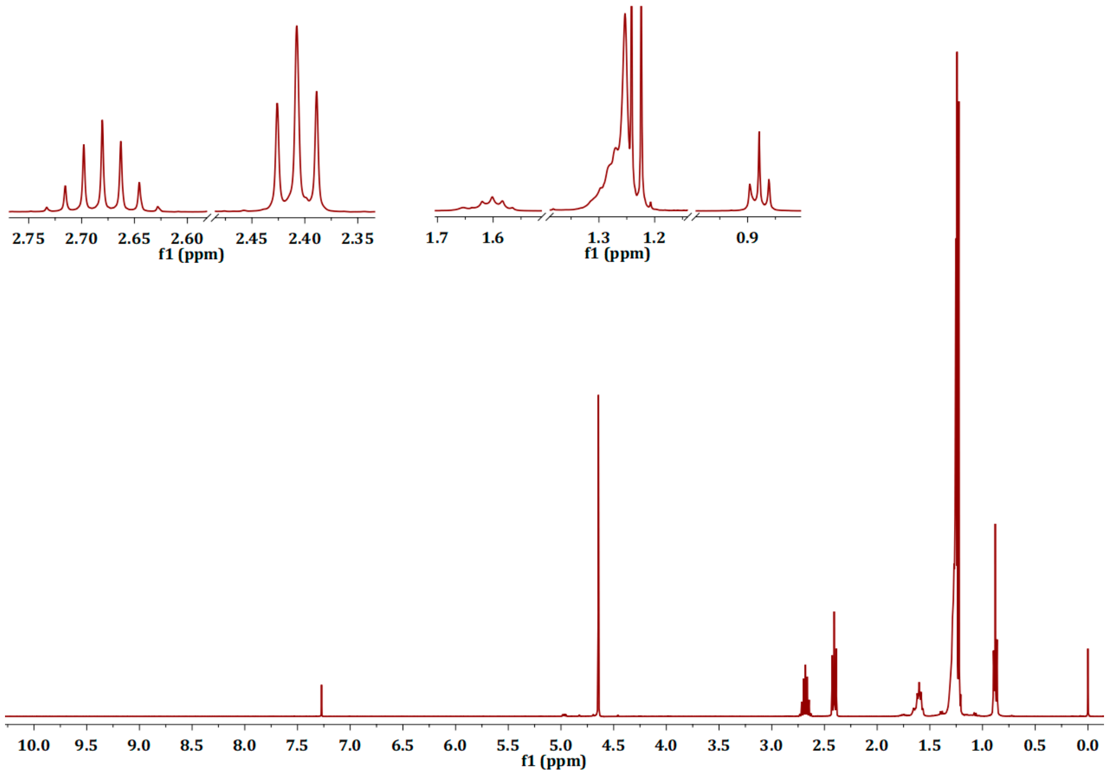

Figure S21. ^{1}H NMR spectrum of 2-oxotridecyl isobutyrate (**3a**) recorded in $CDCl_3$

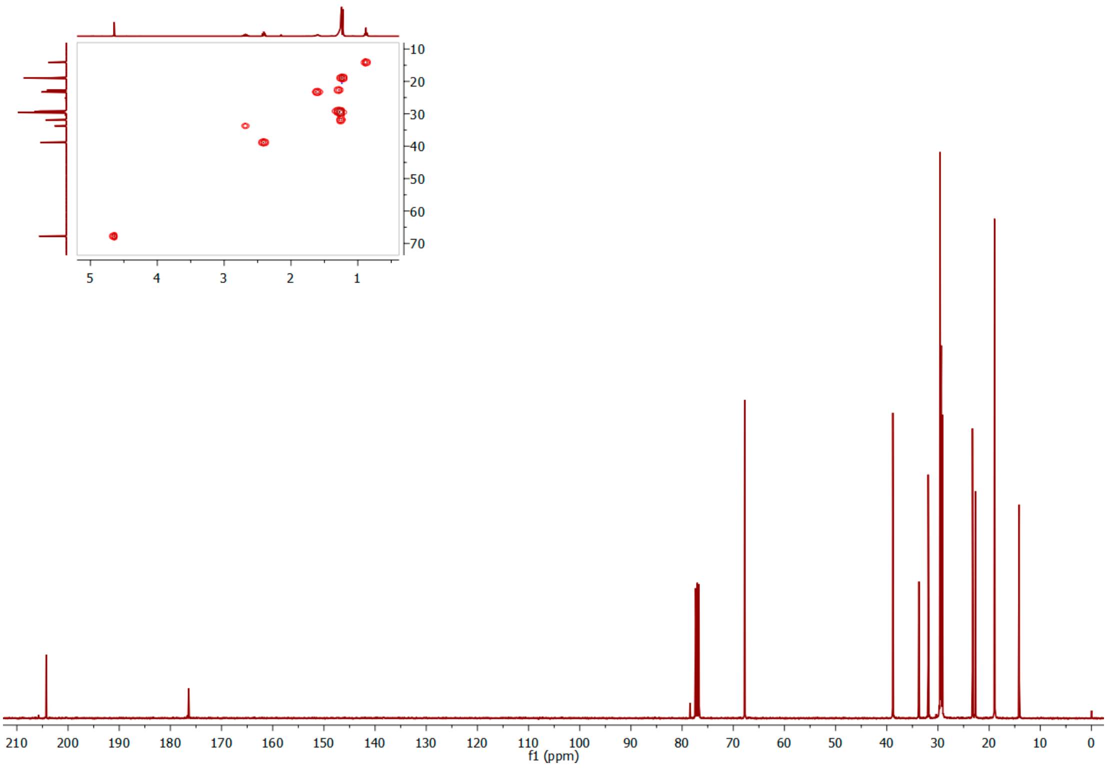

Figure S22. ^{13}C NMR spectrum of 2-oxotridecyl isobutyrate (**3a**) recorded in $CDCl_3$

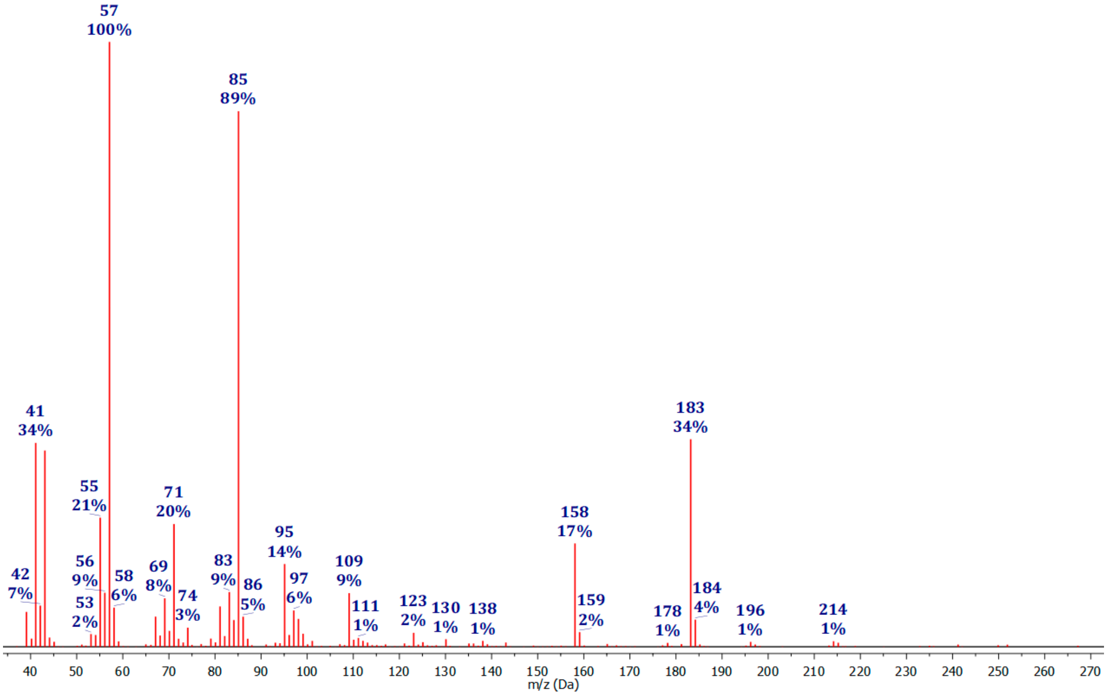

Figure S23. Mass spectrum of 2-oxotridecyl 2-methylbutanoate (**3b**)

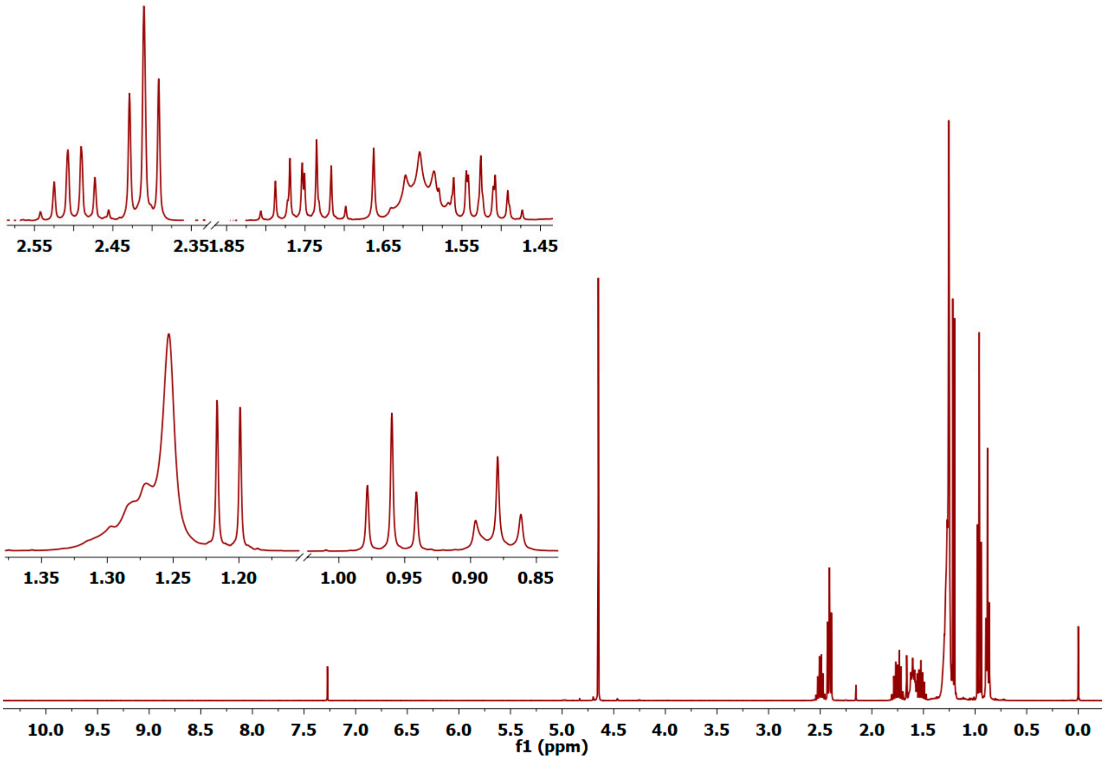

Figure S24. ^{1}H NMR spectrum of 2-oxotridecyl 2-methylbutanoate (**3b**) recorded in $CDCl_3$

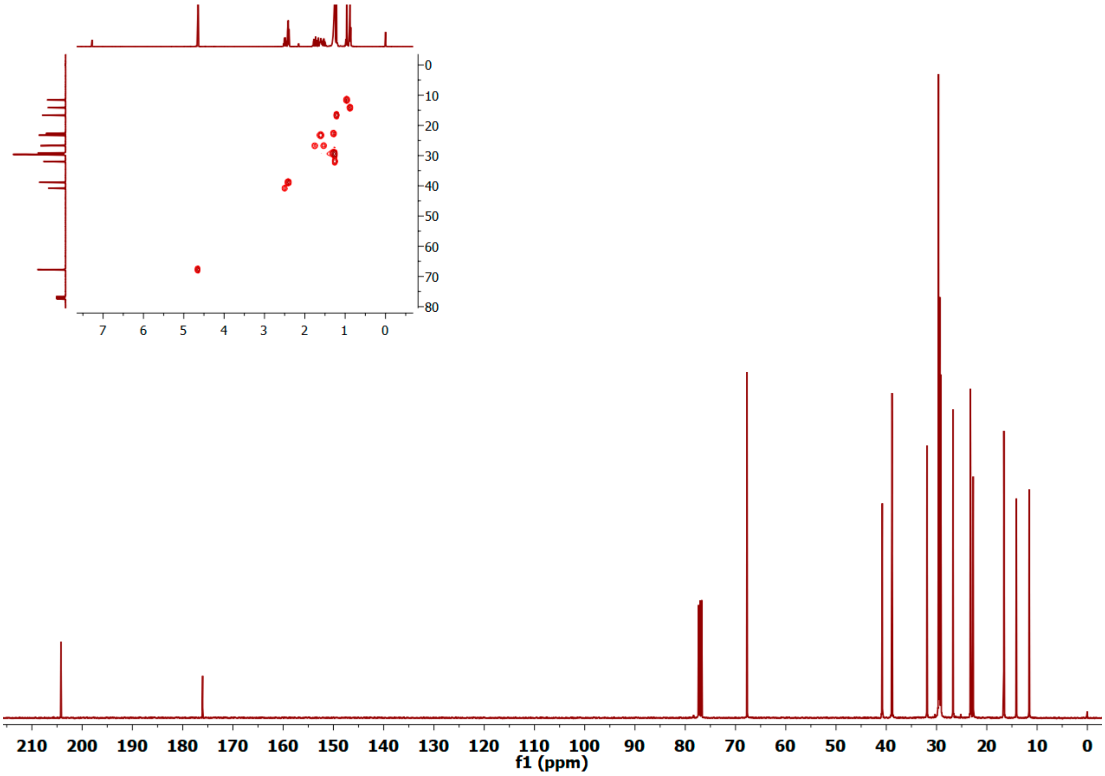

Figure S25. ^{13}C NMR spectrum of 2-oxotridecyl 2-methylbutanoate (**3b**) recorded in $CDCl_3$

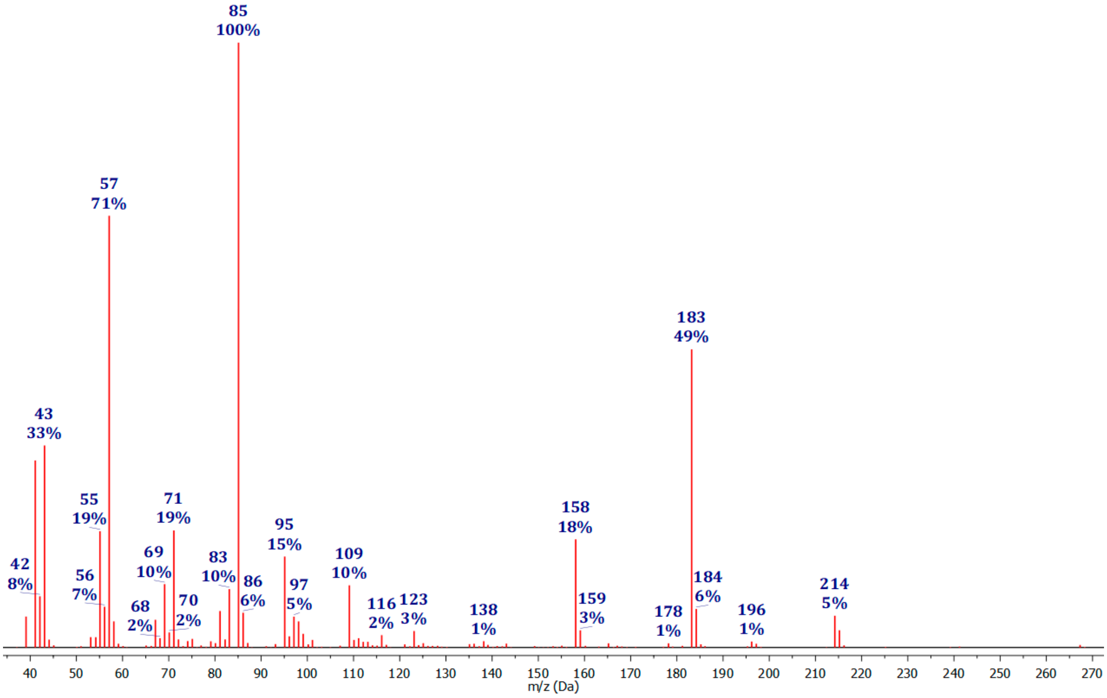

Figure S26. Mass spectrum of 2-oxotridecyl 3-methylbutanoate (**3c**)

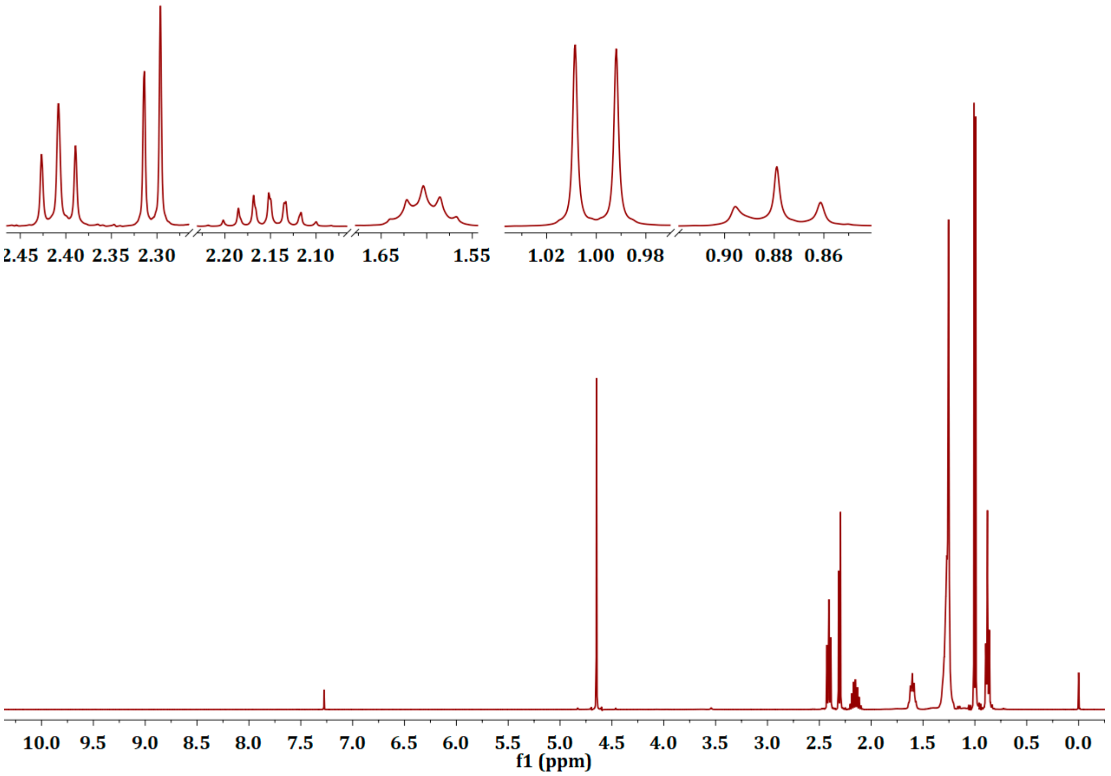

Figure S27. ^{1}H NMR spectrum of 2-oxotridecyl 3-methylbutanoate (**3c**) recorded in $CDCl_3$

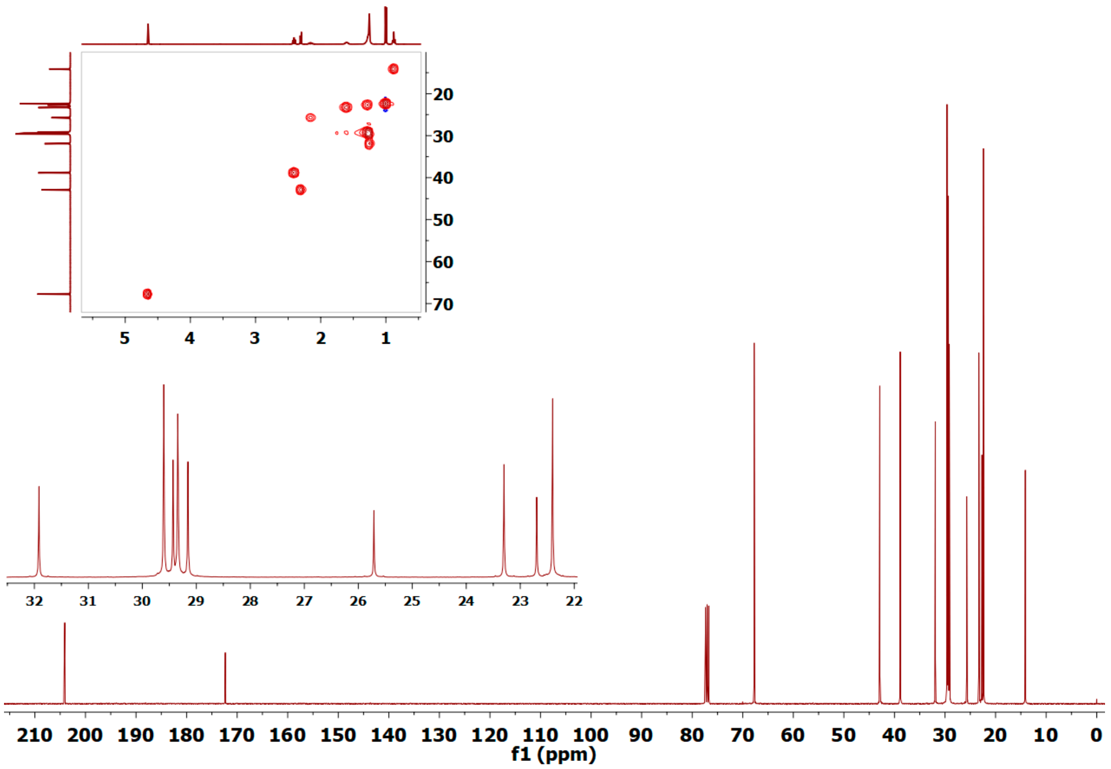

Figure S28. ^{13}C NMR spectrum of 2-oxotridecyl 3-methylbutanoate (**3c**) recorded in $CDCl_3$

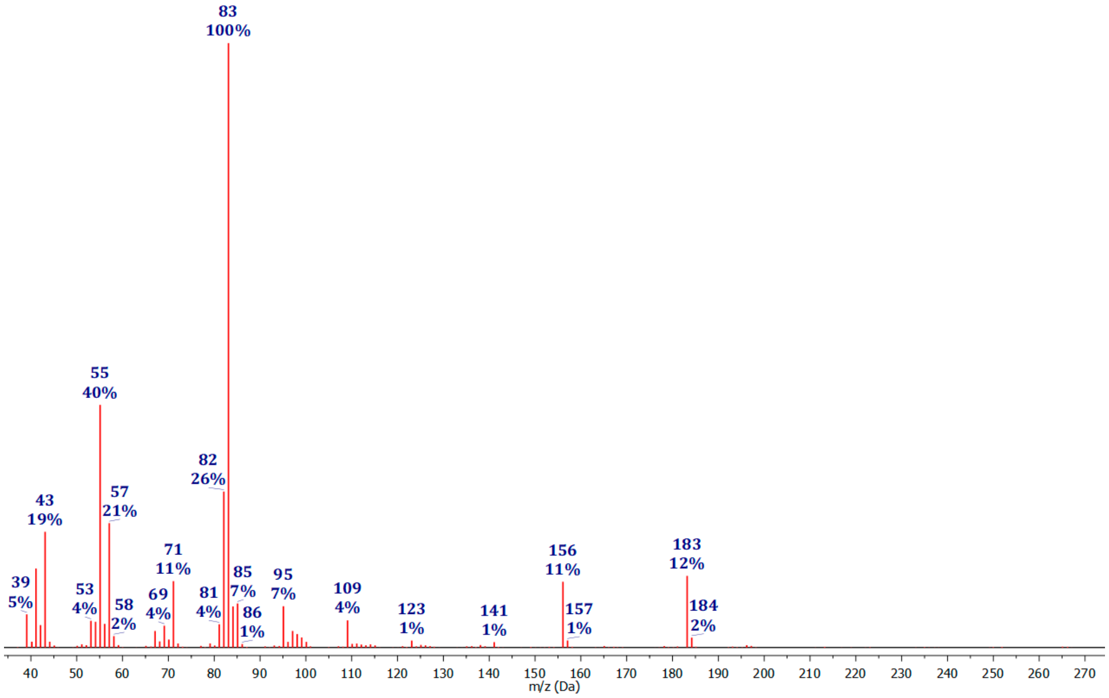

Figure S29. Mass spectrum of 2-oxotridecyl angelate (**3d**)

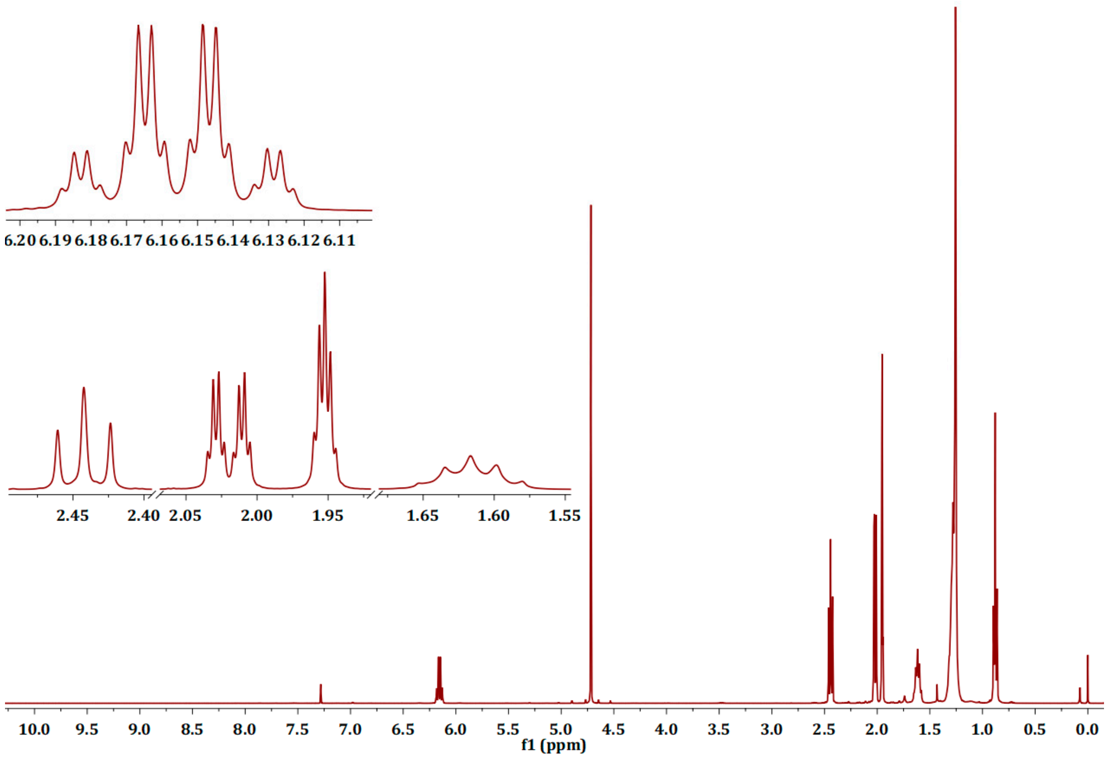

Figure S30. ^{1}H NMR spectrum of 2-oxotridecyl angelate (**3d**) recorded in $CDCl_3$

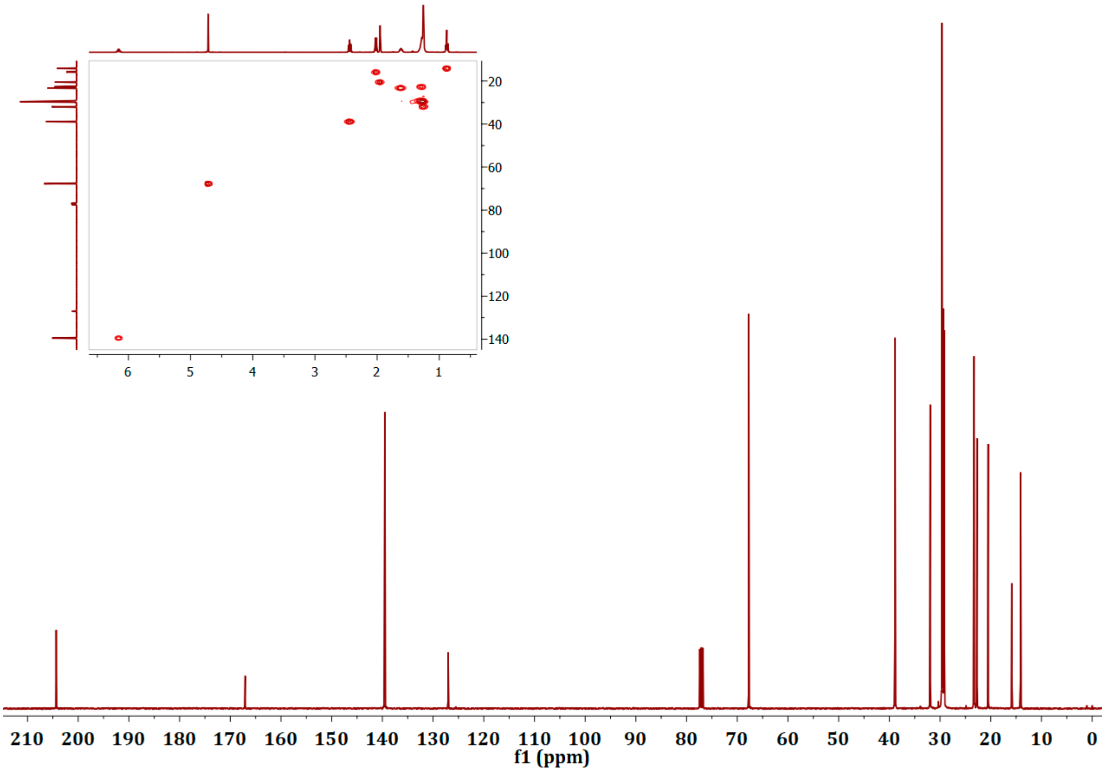

Figure S31. ^{13}C NMR spectrum of 2-oxotridecyl angelate (**3d**) recorded in $CDCl_3$

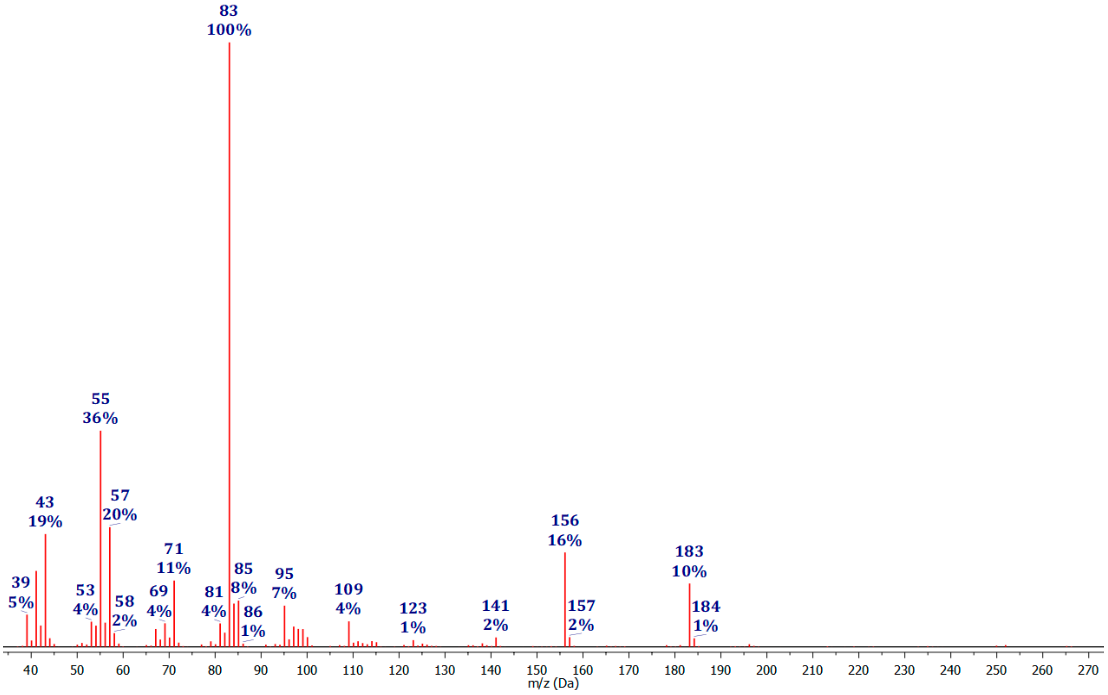

Figure S32. Mass spectrum of 2-oxotridecyl tiglate (**3e**)

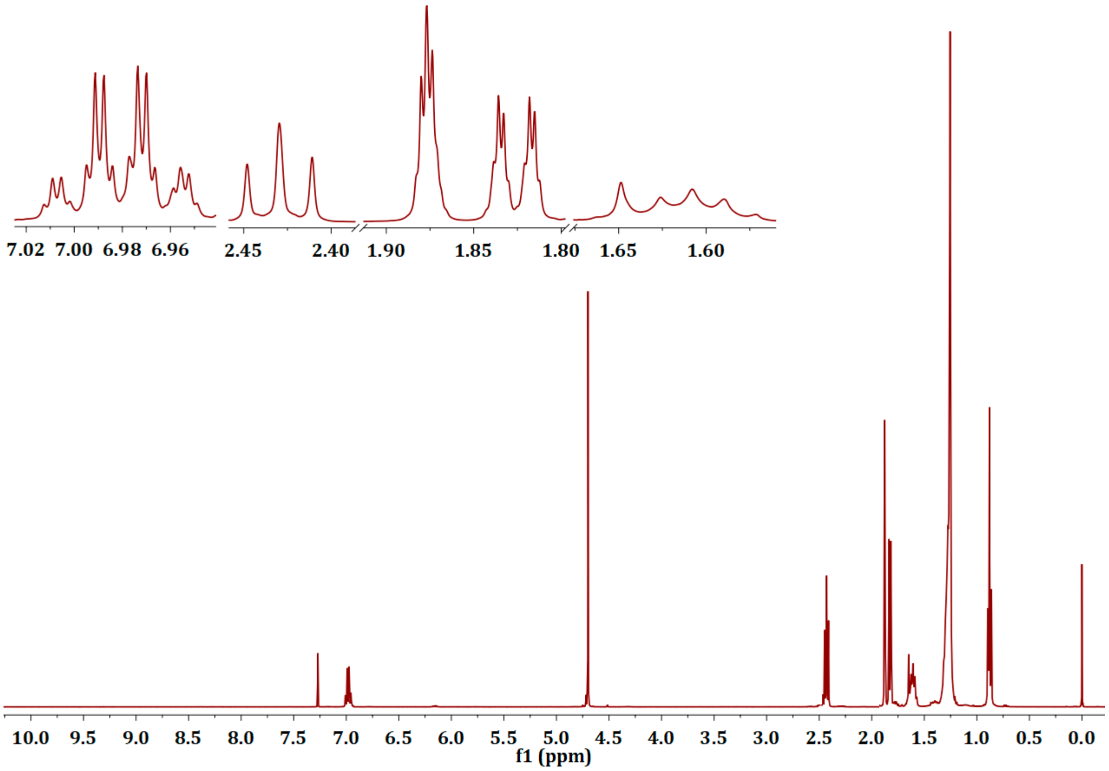

Figure S33. ^{1}H NMR spectrum of 2-oxotridecyl tiglate (**3e**) recorded in $CDCl_3$

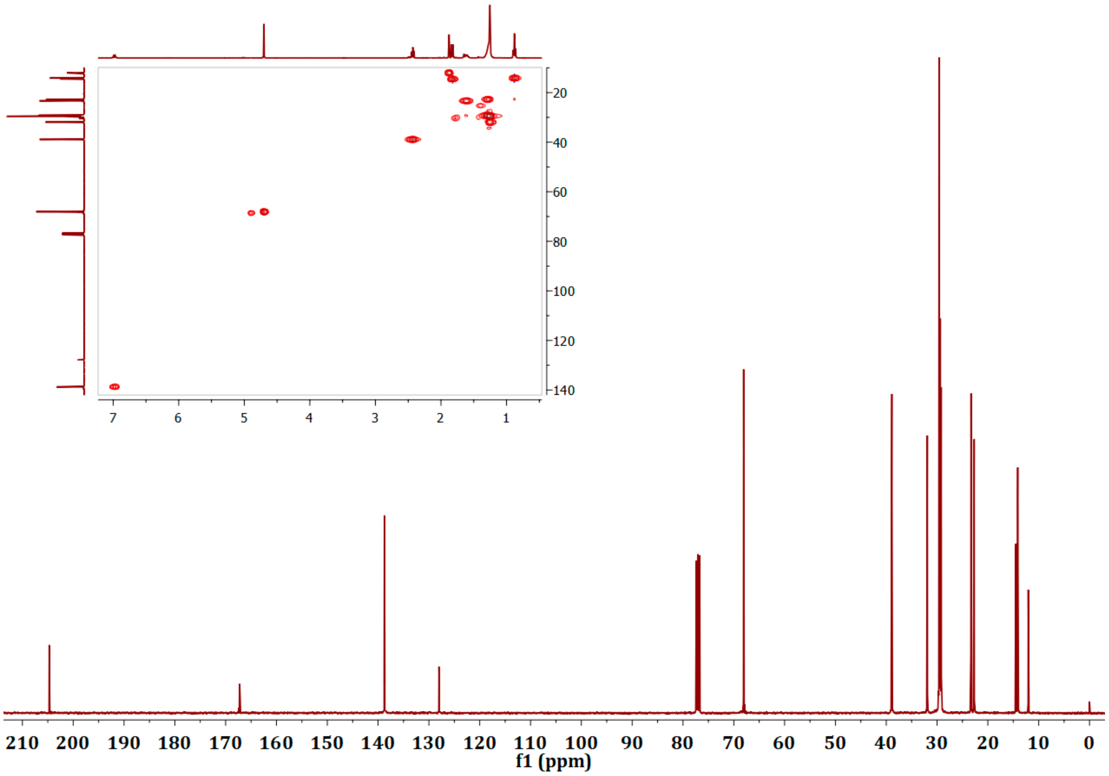

Figure S34. ^{13}C NMR spectrum of 2-oxotridecyl tiglate (**3e**) recorded in $CDCl_3$

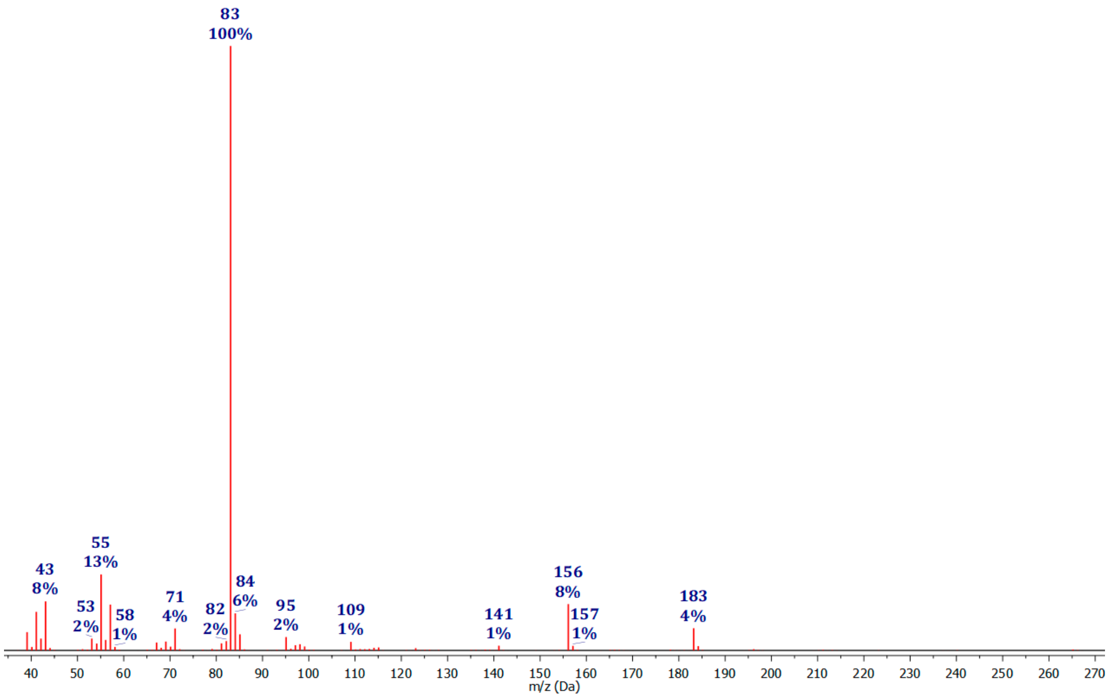

Figure S35. Mass spectrum of 2-oxotridecyl senecioate (**3f**)

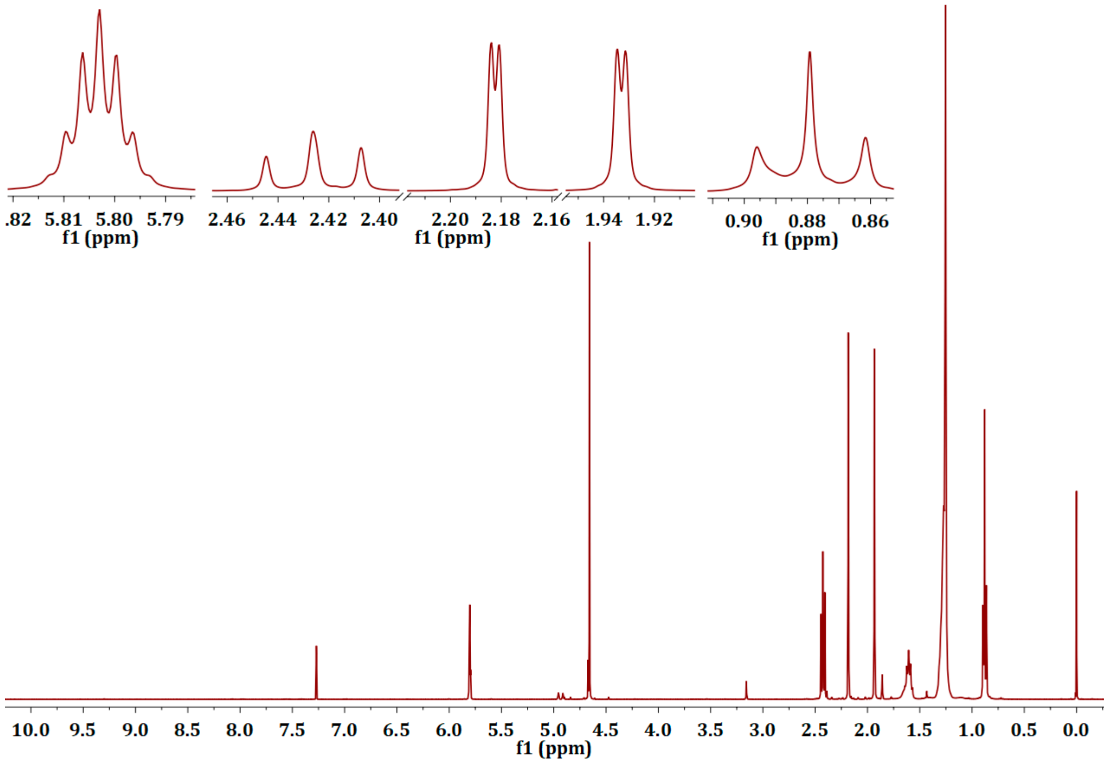

Figure S36. ^{1}H NMR spectrum of 2-oxotridecyl senecioate (**3f**) recorded in $CDCl_3$

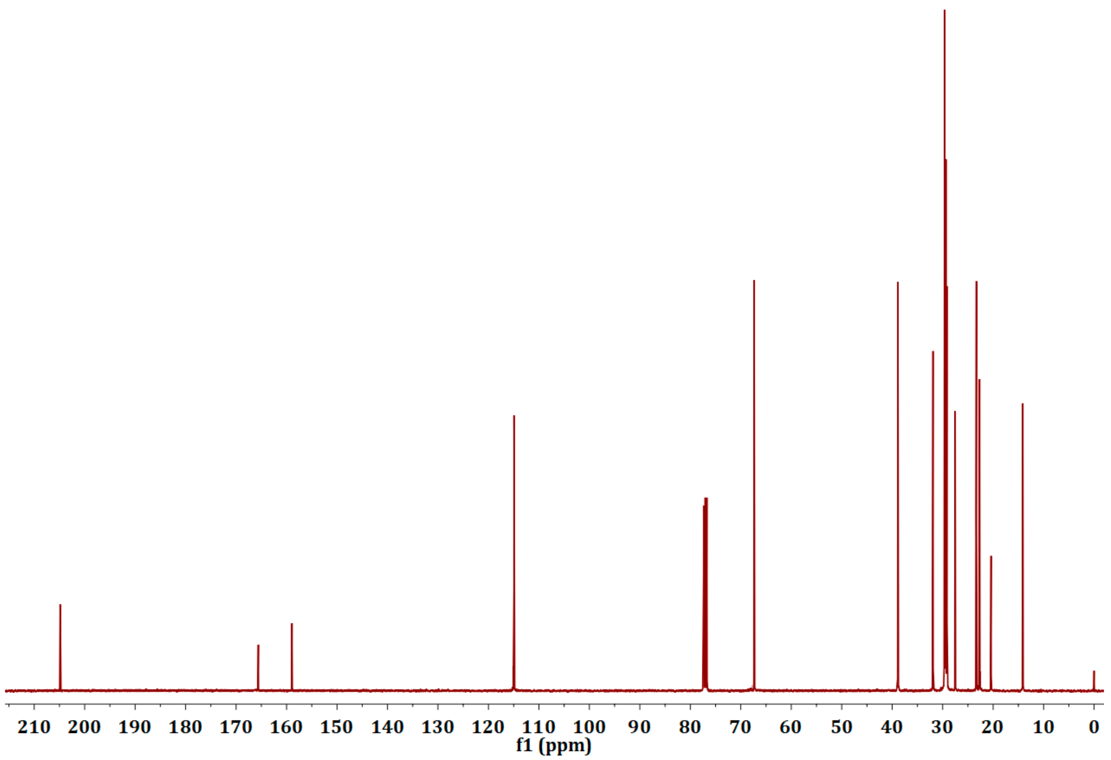

Figure S37. ^{13}C NMR spectrum of 2-oxotridecyl senecioate (**3f**) recorded in $CDCl_3$

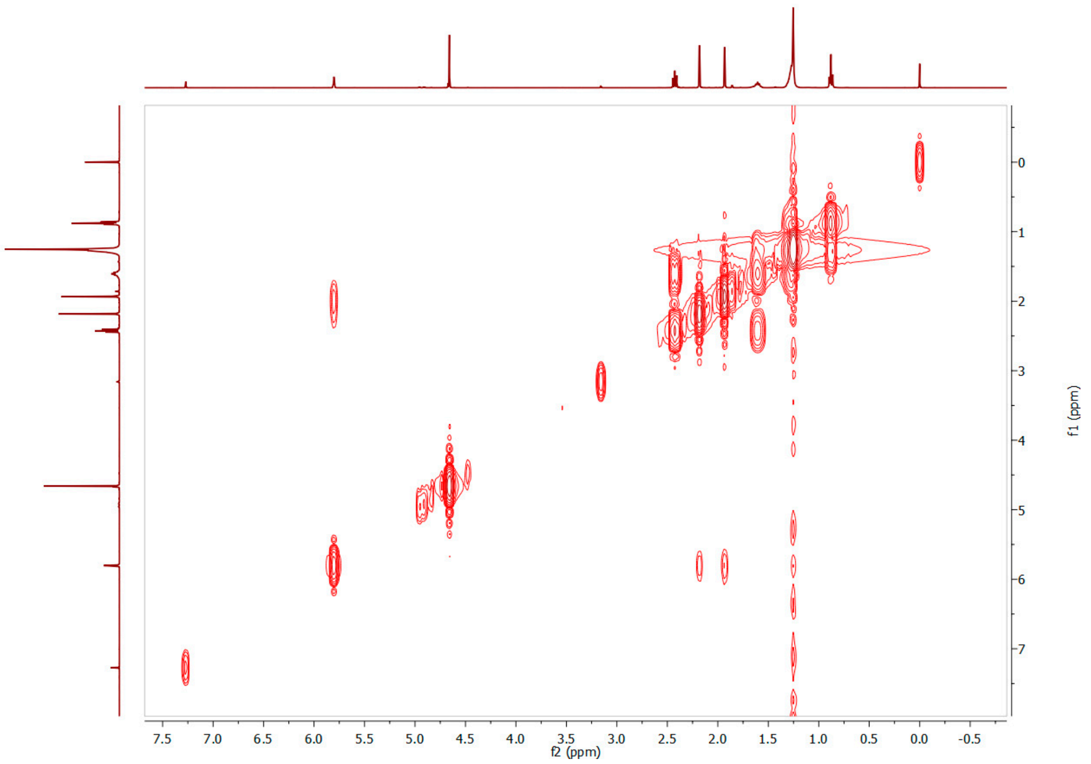

Figure S38. ^{1}H – ^{1}H COSY spectrum of 2-oxotridecyl senecioate (**3f**)

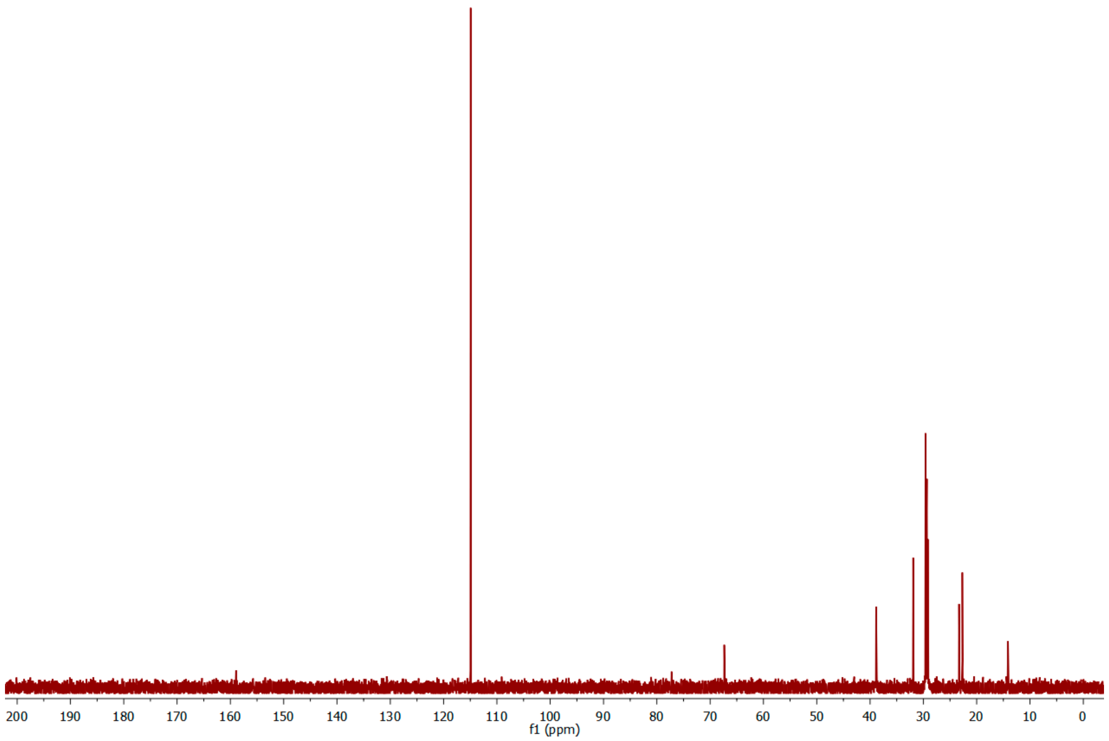

Figure S39. DEPT 90 spectrum of 2-oxotridecyl senecioate (**3f**)

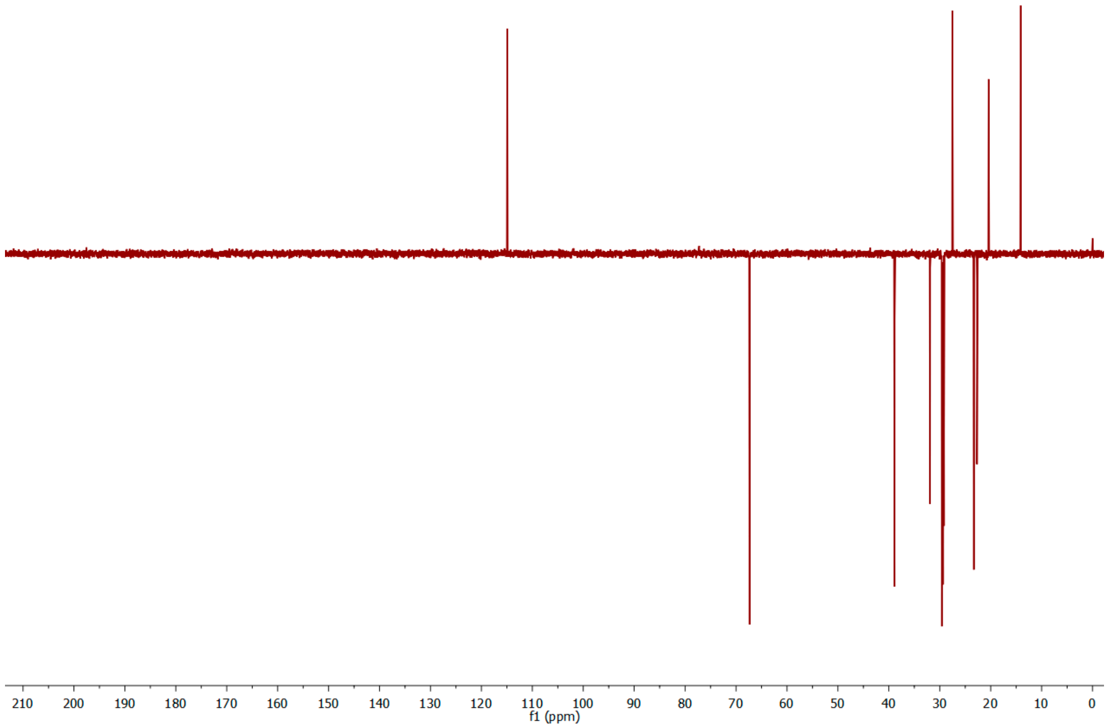

Figure S40. DEPT 135 spectrum of 2-oxotridecyl senecioate (**3f**)

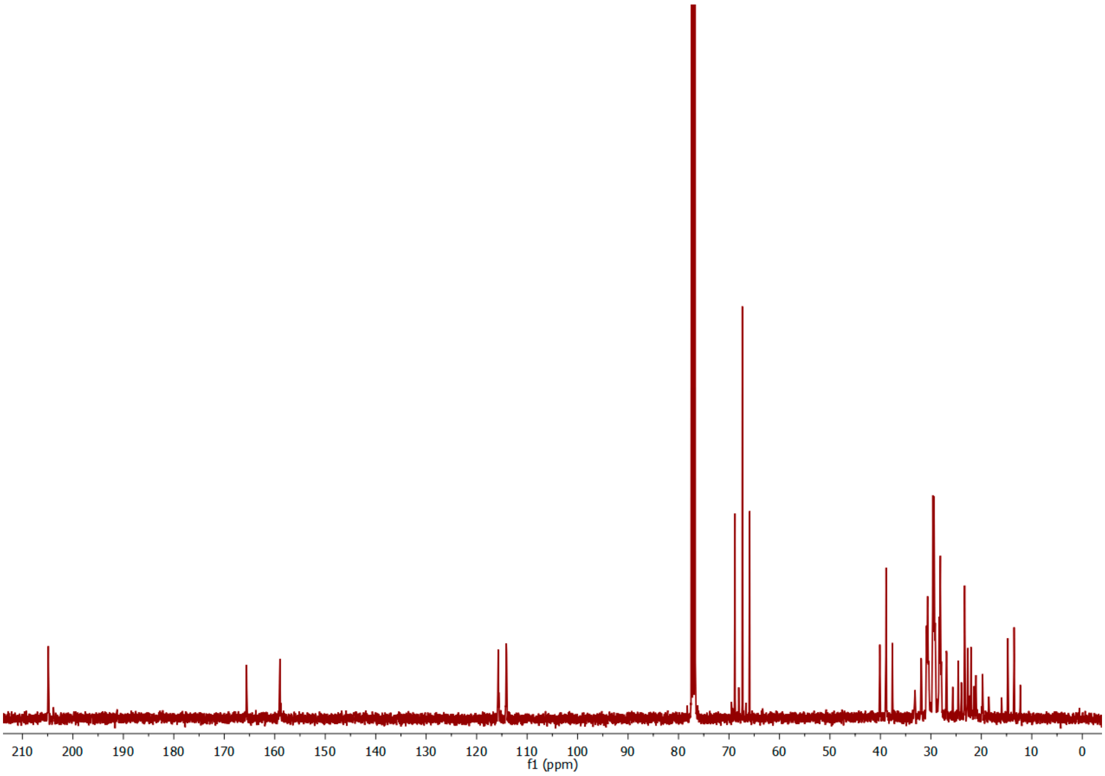

Figure S41. Proton-coupled ^{13}C NMR spectrum of 2-oxotridecyl senecioate (**3f**)

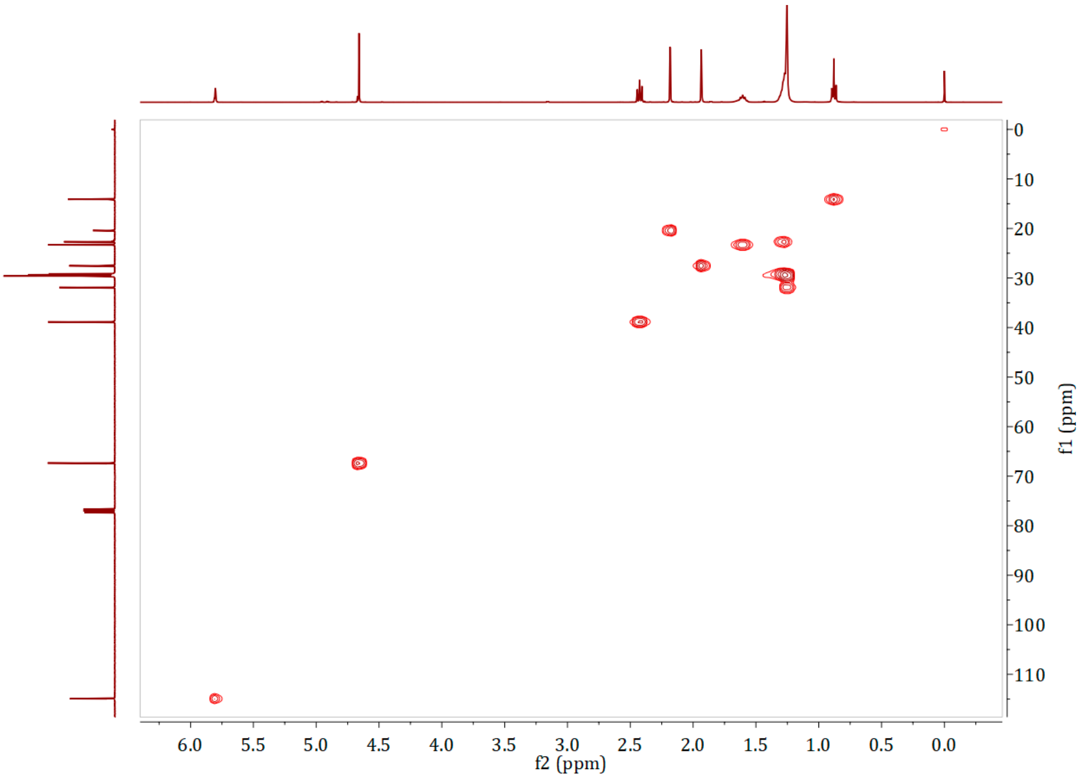

Figure S42. grHSQC spectrum of 2-oxotridecyl senecioate (**3f**)

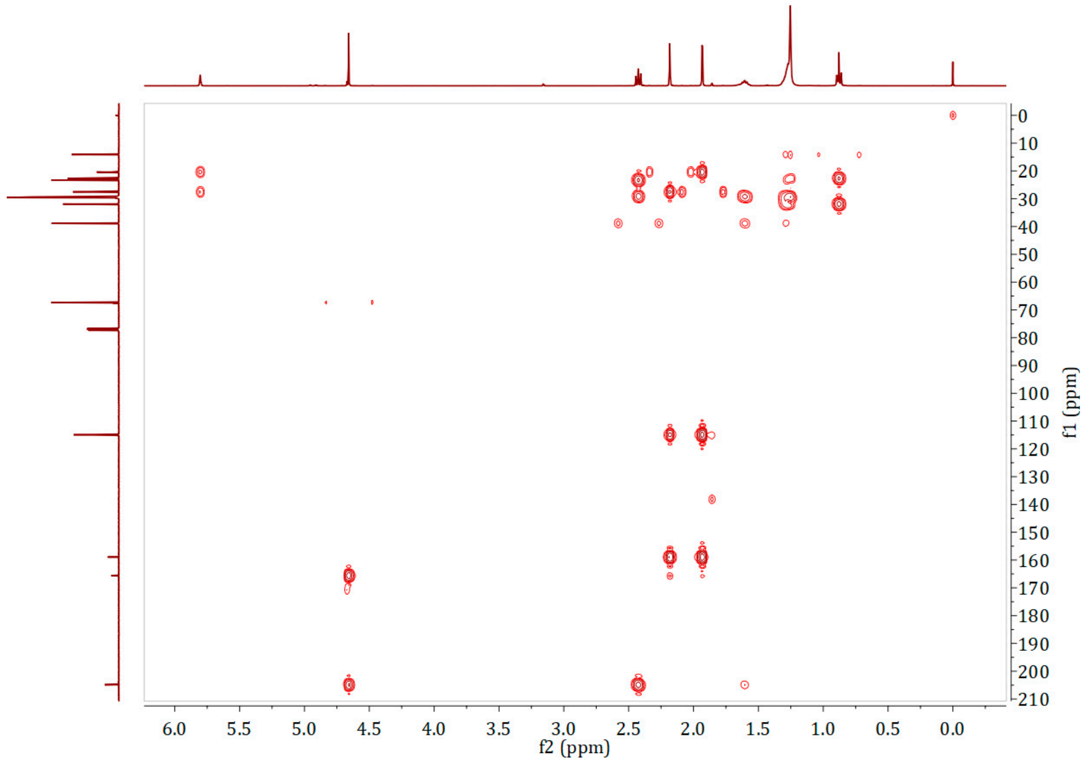

Figure S43. grHMBC spectrum of 2-oxotridecyl senecioate (**3f**)

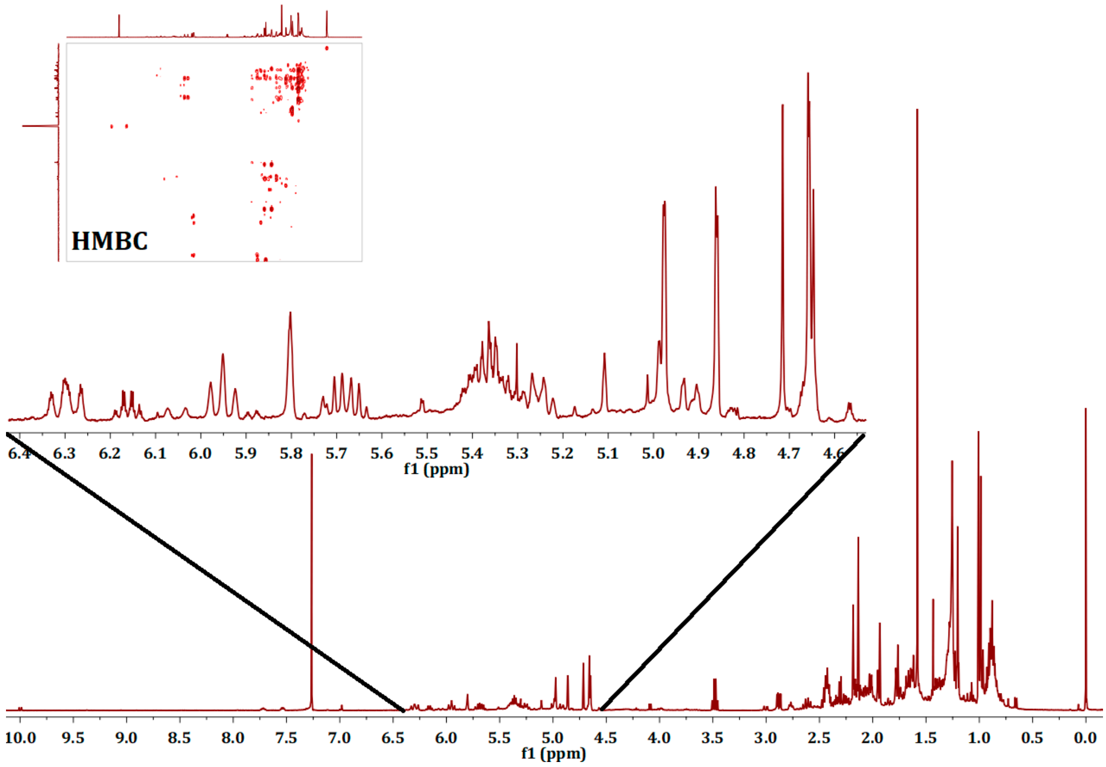

Figure S44. ^{1}H NMR spectrum of the essential oil fraction F5

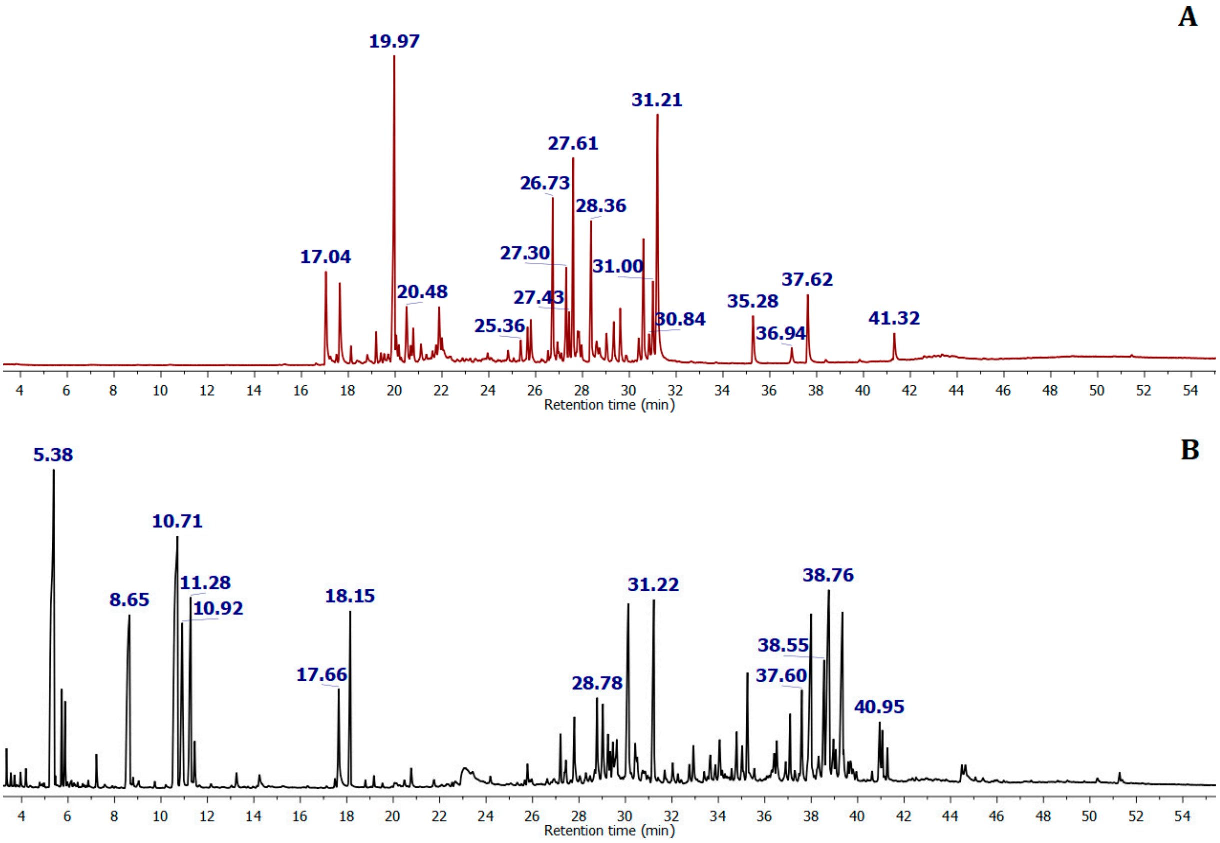

Figure S45. GC chromatogram before (A) and after (B) derivatization of the essential oil fraction F5 with dimethyl disulfide

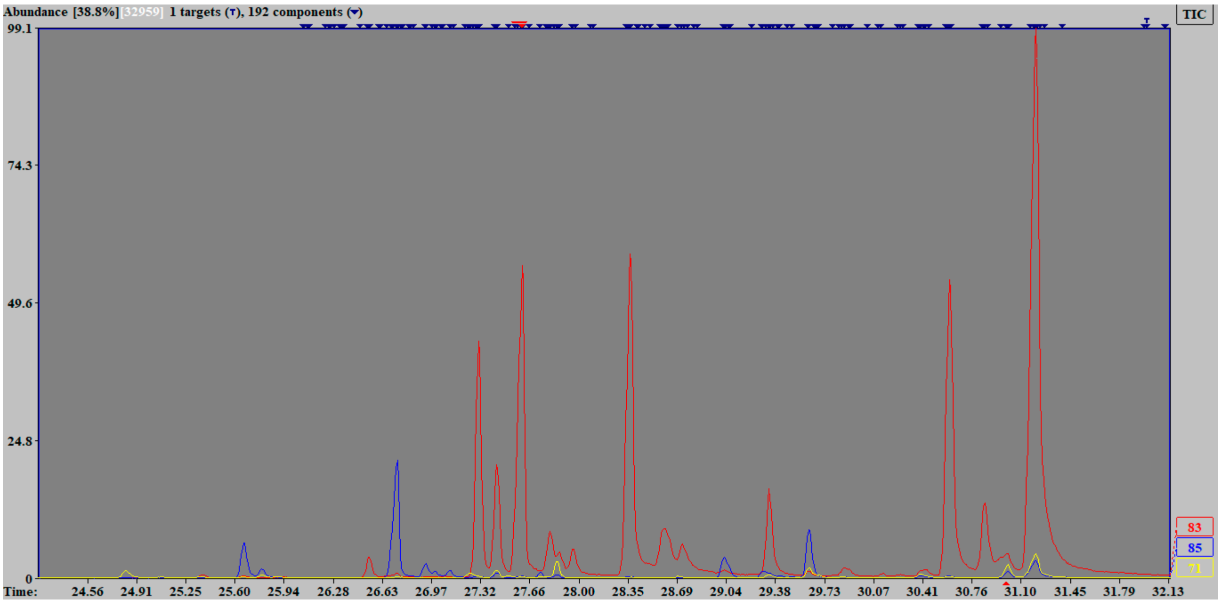

Figure S46. Part of the patrial ion current chromatogram (ca. 24 – 32 min) (PIC, ions at *m/z* 71 (marked yellow), *m/z* 83 (red), and *m/z* 85 (blue) of the chromatographic fraction F5 of *A. oleracea* essential oil

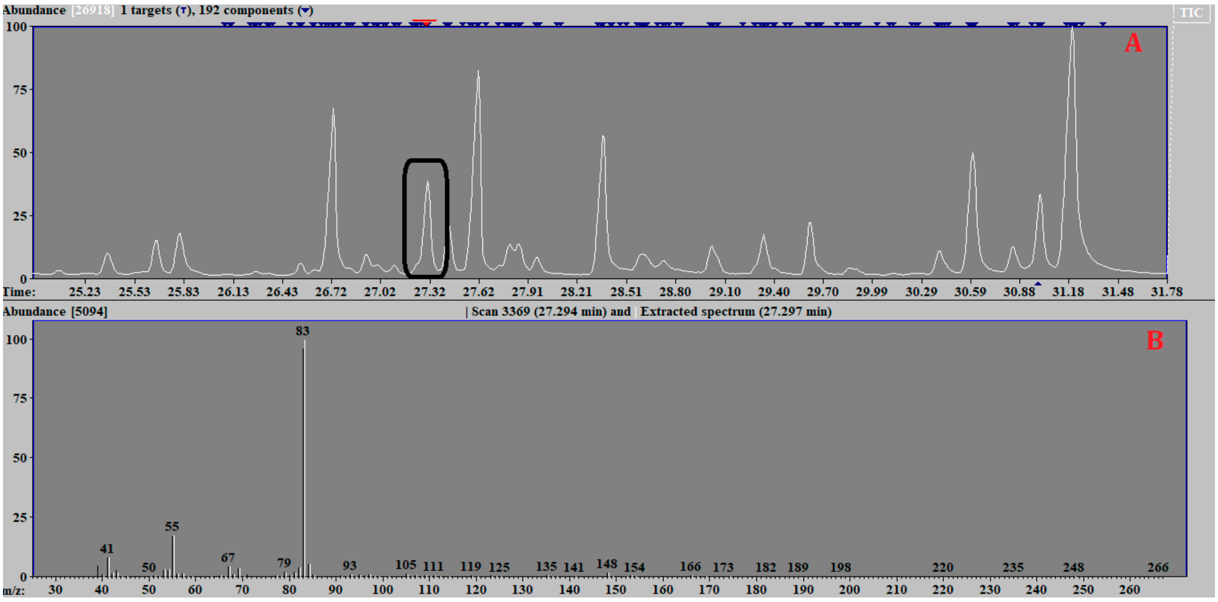

Figure S47. Part of the chromatogram (A) and mass spectrum of the detected 2-oxoundec-7-en-1-yl senecioate (B)

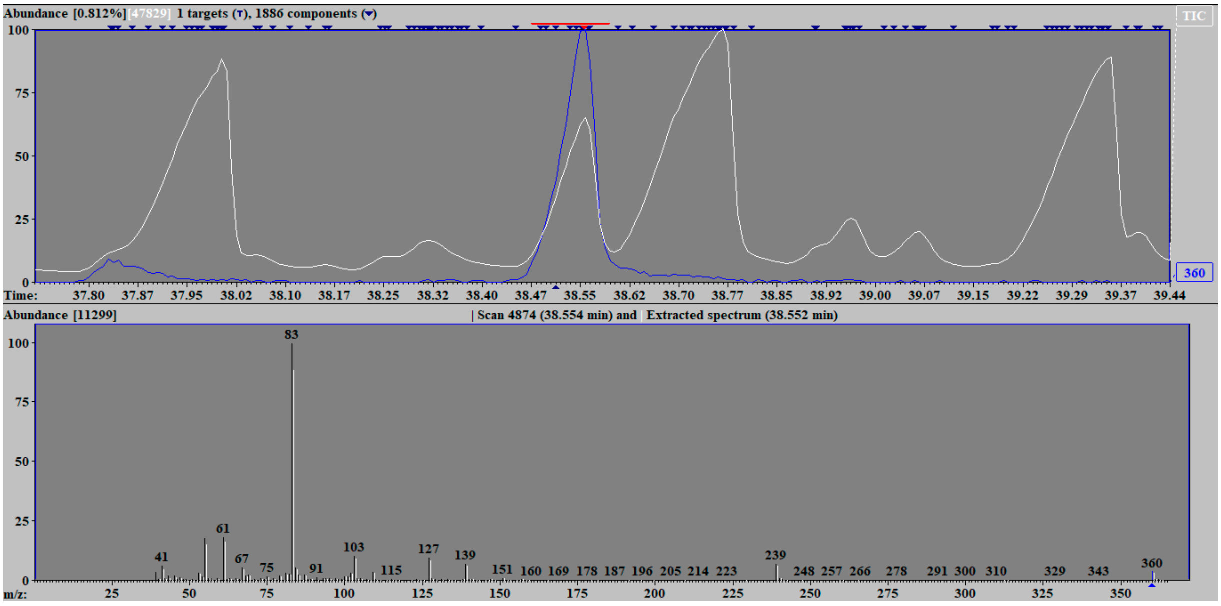

Figure S48. Part of the GC chromatogram after derivatization of the essential oil fraction F5 with dimethyl disulfide and mass spectrum of the dimethyl disulfide adduct of the 2-oxoundec-7-en-1-yl senecioate

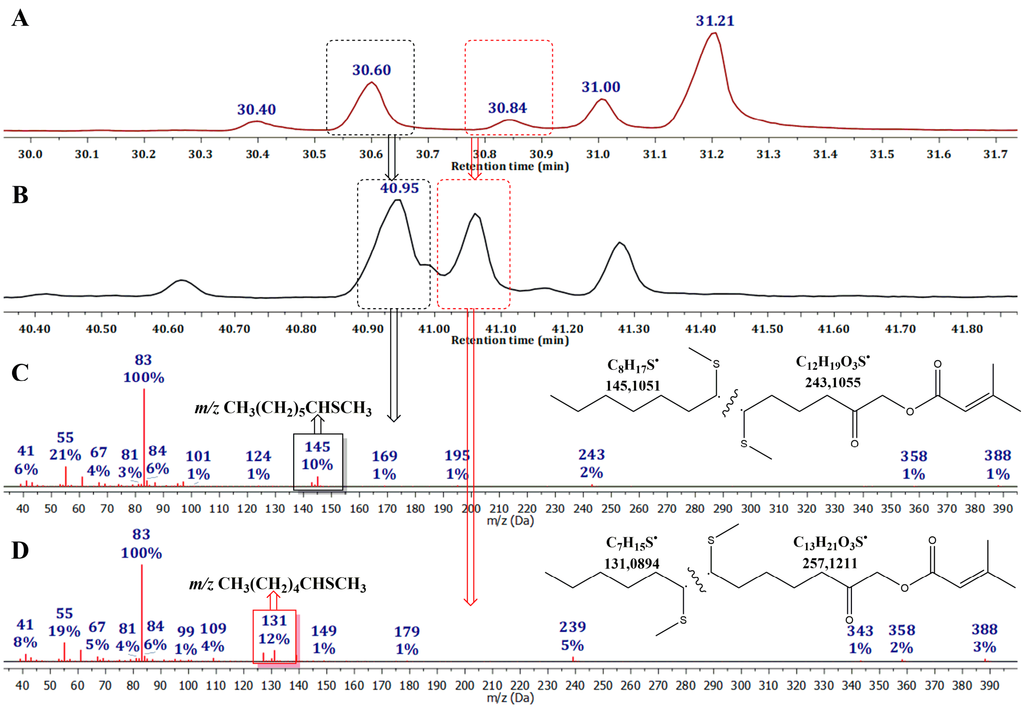

Figure S49. Part of the GC chromatogram before (**A**) and after derivatization of the essential oil fraction F5 with dimethyl disulfide (**B**) and mass spectrum of the dimethyl disulfide adduct of the 2-oxotridec-6-en-1-yl senecioate (**C**) and 2-oxotridec-7-en-1-yl senecioate (**D**)

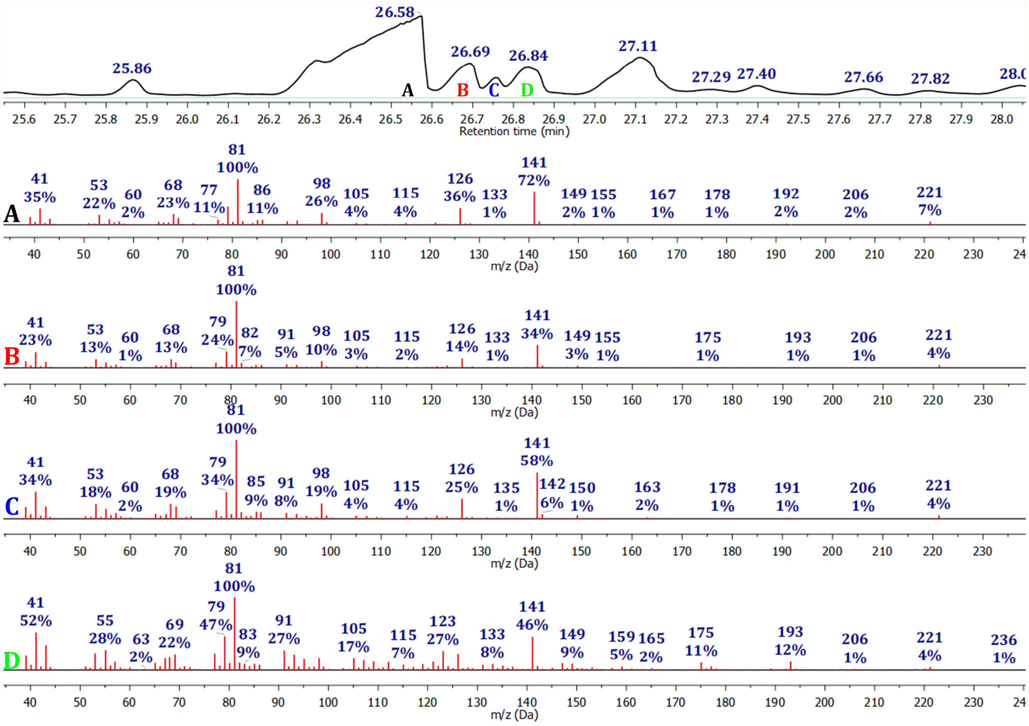

Figure S50. Part of the GC chromatogram of the essential oil fraction F7, mass spectrum of the spilanthol (**A**), and MS of the spilanthol diastereoisomers (**B**, **C**, and **D**)

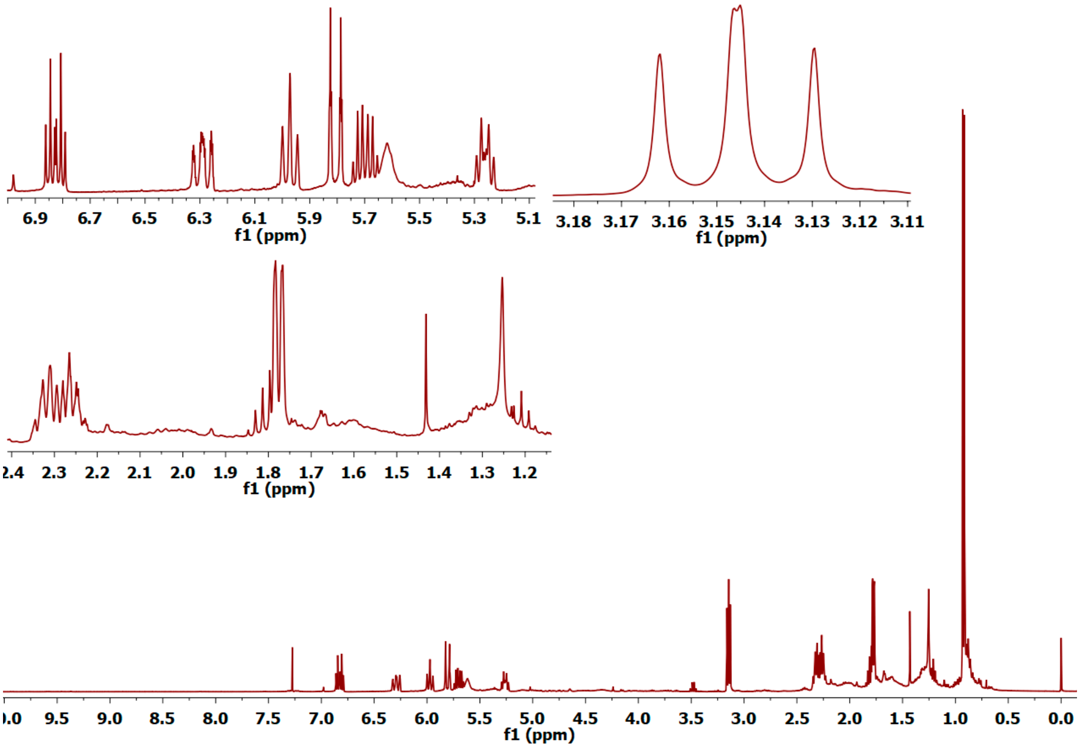

Figure S51. ^{1}H NMR spectrum of the essential oil fraction F7

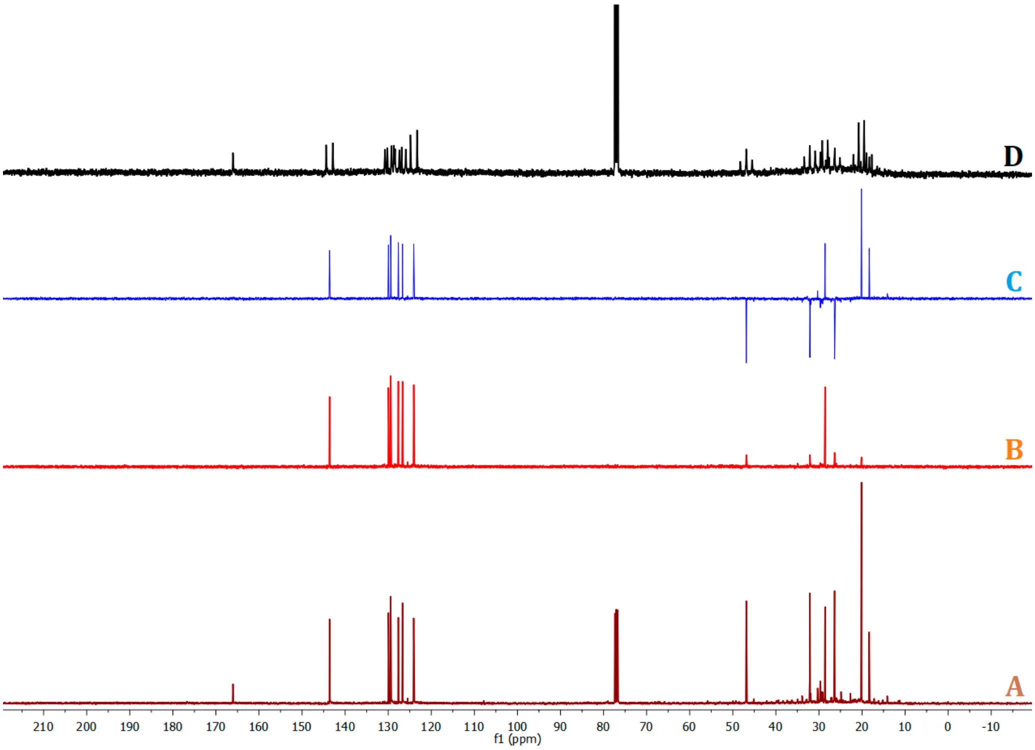

Figure S52. ^{13}C NMR spectrum of the essential oil fraction F7 (**A**), DEPT-90 (**B**), DEPT-135 (**C**), and ^{13}C proton coupled NMR spectrum (**D**)

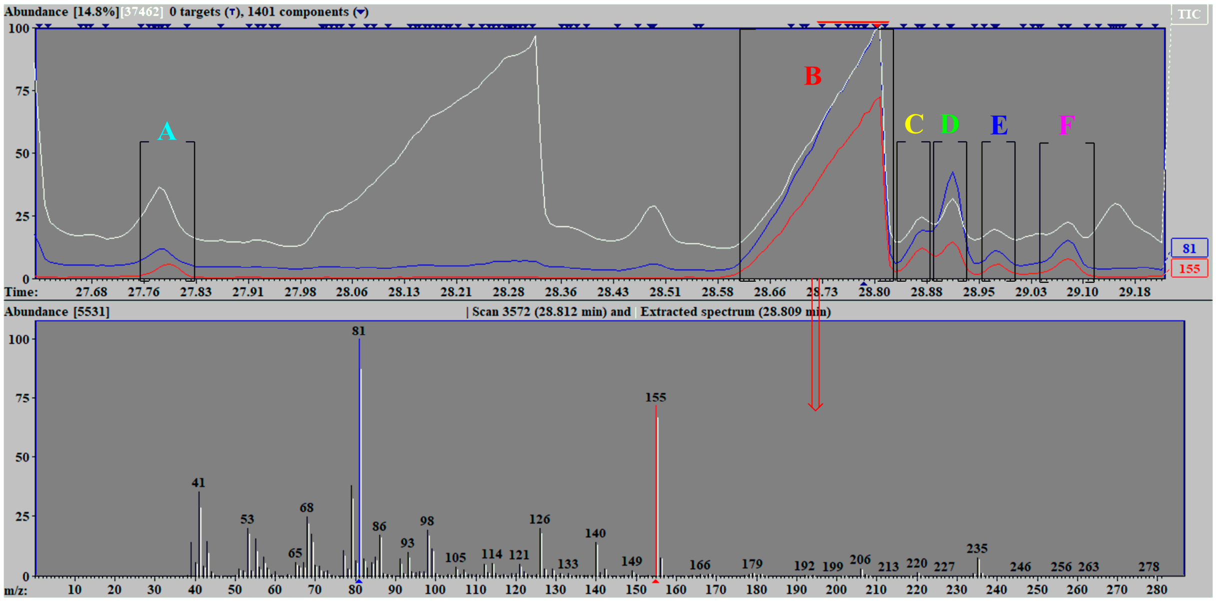

Figure S53. Part of the patrial ion current chromatogram (ca. 27 – 29 min) (PIC, ions at *m*/*z* 81 (marked blue) and 155 (red) of the chromatographic fraction F7 of *A. oleracea* essential oil and mass spectrum of one of the detected isomeric *N*-(2-methylbutyl)decatrienamide
